# Structure-reactivity analysis of novel hypervalent iodine reagents in *S*-vinylation of thiols

**DOI:** 10.3389/fchem.2024.1376948

**Published:** 2024-02-29

**Authors:** Sayad Doobary, Ester Maria Di Tommaso, Alexandru Postole, A. Ken Inge, Berit Olofsson

**Affiliations:** ^1^ Department of Organic Chemistry, Stockholm University, Stockholm, Sweden; ^2^ Department of Materials and Environmental Chemistry, Stockholm University, Stockholm, Sweden

**Keywords:** alkenes, benziodoxolones, hypervalency, reduction potential, X-ray crystallography, structure-reactivity analysis, VBX

## Abstract

The transition-metal free *S*-vinylation of thiophenols by vinylbenziodoxolones (VBX) constituted an important step forward in hypervalent iodine-mediated vinylations, highlighting the difference to vinyliodonium salts and that the reaction outcome was influenced by the substitution pattern of the benziodoxolone core. In this study, we report several new classes of hypervalent iodine vinylation reagents; vinylbenziodazolones, vinylbenziodoxolonimine and vinyliodoxathiole dioxides. Their synthesis, structural and electronic properties are described and correlated to the *S*-vinylation outcome, shedding light on some interesting facets of these reagents.

## Graphical Abstract

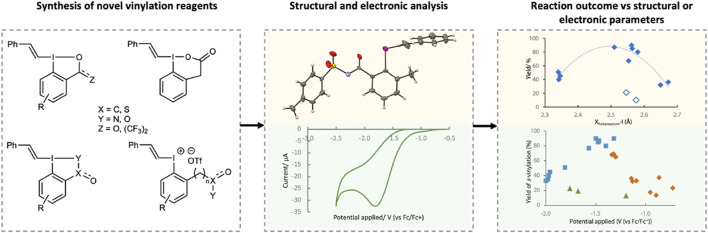

## 1 Introduction

Hypervalent iodine reagents have been shown to be powerful reagents for chemoselective transformations under both transition metal-catalyzed and metal-free conditions. (Wirth, 2016; Yoshimura and Zhdankin, 2016; Flores et al., 2019; Olofsson et al., 2019). The use of iodonium salts has enabled transfer of aryl, alkynyl and vinyl groups to a variety of nucleophiles. (Merritt and Olofsson, 2009; Charpentier et al., 2015; Li et al., 2016; Rajkiewicz and Kalek, 2018; Villo et al., 2019; Declas et al., 2020; Dahiya et al., 2022; Le Du and Waser, 2023; Mironova et al., 2023; Yoshimura et al., 2023). New classes of alkenes have been accessed through the combination of vinyliodonium salts with metal catalysts, (Skucas and MacMillan, 2012; Holt and Gaunt, 2015; Sheng et al., 2017; Yuan et al., 2019), whereas metal-free applications with those reagents remain scarce due to difficulties in controlling the reaction outcome. (Ochiai et al., 2001; Hara et al., 2006; Kepski et al., 2019). Recent developments in the field have shown that benziodoxolones (BX), which are iodine (III) compounds with a cyclic core, possess improved stability and often have more easily controlled reactivity. (Yoshimura et al., 2023). Indeed, the utility of trifluoromethyl-BX (Togni’s reagent) and ethynyl-BX (EBX) have been efficiently demonstrated in the last decades. (Charpentier et al., 2015; Hari et al., 2019; Le Du and Waser, 2023).

**FIGURE 1:**
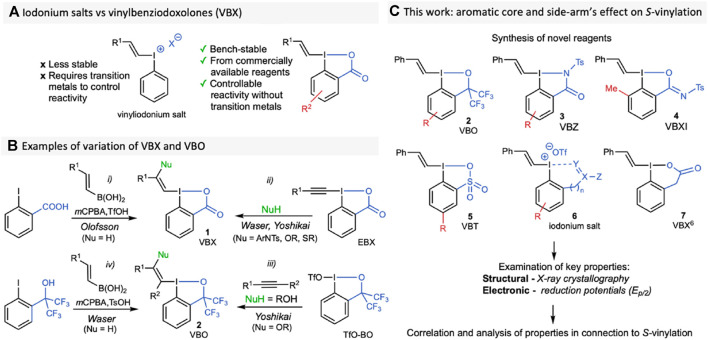
**(A)** The use of iodonium salts compared to VBX; **(B)** Selected synthetic routes to VBX and VBO; **(C)** Workflow of this investigation.

In 2016, our group reported the synthesis and first applications of vinyl-BX (VBX, **1**) (Stridfeldt et al., 2016), which showed enhanced reactivity and selectivity compared to vinyliodonium salts. (Figure 1A), (Stridfeldt et al., 2016; Declas et al., 2020). Transition metal-free applications include *S-* and *P-*vinylation methodologies, (Castoldi et al., 2020a; Castoldi et al., 2020b; Di Tommaso et al., 2022), as well as photocatalytic *C*-vinylations with redox active compounds (Pal et al., 2023) and others. (Davies et al., 2017; Le Vaillant et al., 2018; Liu et al., 2018; Jiang and Studer, 2019; Li et al., 2019; Liu et al., 2020). Our one-pot synthesis of VBX is shown in Figure 1B *i*, and the scope was later expanded to include β-heteroatom-functionalized VBX through addition of a nucleophile and a proton over EBX (Figure 1B *ii*). (Frei et al., 2014; Caramenti et al., 2019; Shimbo et al., 2019; Tessier et al., 2019; Wu et al., 2019; Liu et al., 2020; Declas et al., 2022) In parallel, the corresponding vinylbenziodoxoles with a bis(CF_3_)alkoxy moiety (VBO) were introduced by Yoshikai and coworkers, and have proved superior in some applications. (Wu et al., 2016; Wu J. et al., 2017; Shimbo et al., 2019; Wu et al., 2019; Chai et al., 2021; Wang et al., 2022). VBO can be synthesized from TfO-BO and mono- or di-substituted alkynes (Figure 1B *iii*). (Wu B. et al., 2017; Ding et al., 2020; Ura et al., 2020; Chai et al., 2021; Declas et al., 2022; Kikuchi et al., 2022; Wang et al., 2022) Recently, Waser presented a one-pot synthesis of ethynyl bis(trifluoromethyl)iodoxole (EBO) directly from the corresponding iodoarene. (Milzarek et al., 2023). This method also included the synthesis of a VBO reagent, simplifying the access to such targets (Figure 1B *iv*).

The reactivity of hypervalent alkynylating reagents with substituted aromatic ring cores, as well as variations of the “side-arm”, which binds to the iodine centre, have been explored. (Fernández González et al., 2013; Shimbo et al., 2019). While we evaluated the influence of core-substituents on VBX in the *S*-vinylation of thiols, (Castoldi et al., 2020b), there are no broad studies investigating the structural and electronic effects of varying these groups on vinylating reagents. (Mironova et al., 2023). Herein, we report the synthesis of several new VBO **2**, as well as the synthesis of novel compound classes vinylbenziodazolones (VBZ, **3**), vinylbenziodoxolonimine (VBXI, **4**), and vinyliodoxathiole dioxides (VBT, **5**) (Figure 1C). It should be noted that the benziodazolone (Le Du et al., 2021) and benziodoxathiole (Koser et al., 1993; Koposov et al., 2006) cores have been reported in other hypervalent iodine reagents, whereas the benziodoxolonimine is a novel side-arm. Additionally, we have synthesised several novel *ortho*-functionalized iodonium salts **6**, which serve as good comparisons in the studies. Finally, a vinylbenziodoxolone-type reagent with a six-membered side-arm (VBX^6^ **7**) was synthesized to evaluate the effect of the side-arm length and conjugation with the core. We have determined their crystal structures, as well as their reduction potentials, and correlated these parameters with the reagents’ reactivity under the reported *S*-vinylation conditions.

## 2 Results and discussion

### 2.1 Synthesis of novel vinylation reagents

**SCHEME 1:**
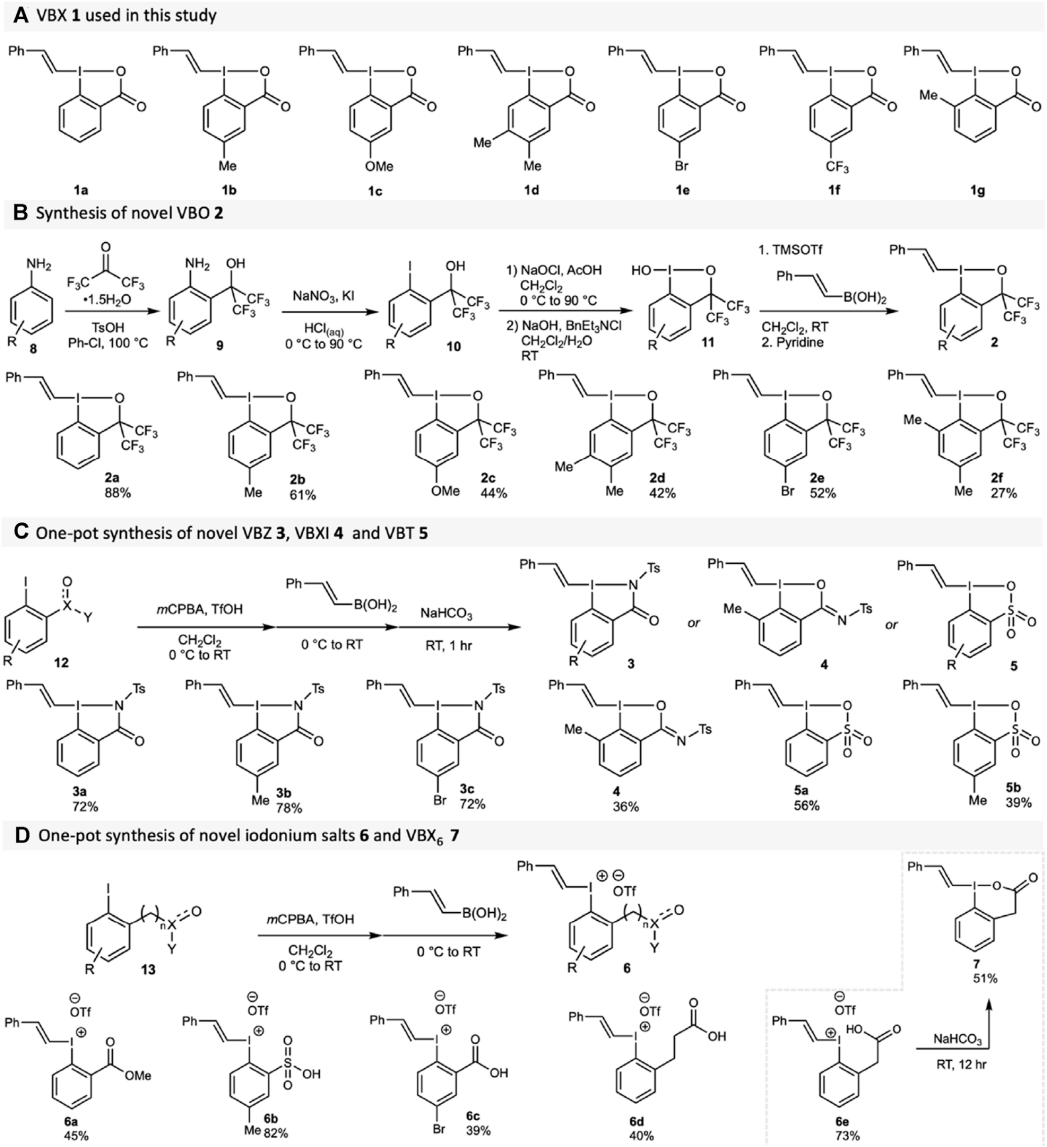
Synthesis of hypervalent vinylation reagents. **(A)** VBX reagents **1**; **(B)** VBO reagents **2** (**2a** already reported), yields from **10** given; **(C)** VBZ reagents **3**, VBXI reagent **4** and VBT reagents **5**; **(D)** Acyclic iodonium salts **6** and VBX^6^ **7**.

Several core-substituted VBX reagents **1** were synthesised according to literature methods (Stridfeldt et al., 2016; Boelke et al., 2017), Scheme 1A). The synthesis of novel VBO reagents **2** started from anilines **8**, which underwent a Friedel-Crafts reaction to access the amino benzyl alcohols **9** (Scheme 1B). A subsequent Sandmeyer reaction produced the required iodoarenes **10** in good yields. (Amey and Martin, 1979). The benziodoxole core was formed through oxidative chlorination and hydrolysis, without isolation in between the steps, to form hydroxy-BO **11**. The vinyl moiety was introduced from the corresponding boronic acid using TMSOTf and pyridine (Boelke et al., 2017) to form VBO **2** (yields from **10** given in Scheme 1B). It should be noted that *ortho*-substituted compound **2f** was incredibly unstable, making isolation and analysis difficult (See the Supplementary Material for details).

Variations of the side-arm were next investigated to obtain novel compound classes for vinylation. The synthesis of VBZ **3** proceeded in good yields from 2-iodophenyl *N*-tosylbenzamides **12a-c**, using our one-pot method developed for VBX (Stridfeldt et al., 2016) with *m*CPBA/triflic acid and (*E*)-styrylboronic acid (Scheme 1C). To our surprise, reactions with the *ortho*-methyl-substituted substrate **12d** behaved differently, and resulted in the formation of the novel compound class VBXI **4**, which has an I-O hypervalent bond instead of the expected I-N bond. It appears that the sterical congestion caused by the *ortho*-methyl group promotes formation of the BXI core as opposed to the BZ core. Products **5** were obtained from 2-iodophenyl sulfonic acids **12d-e** in moderate yields due to incomplete conversion of the starting material.

For the sake of comparison to their cyclic counterparts, a series of vinyliodonium salts **6** with *ortho* functionalities were also synthesised from the iodoarenes **13** (Scheme 1D). Our one-step method (Stridfeldt et al., 2016) without the basic workup was used to obtain these compounds in good to high yields. Interestingly, when the one-pot synthesis of VBX^6^ **7** was attempted, product formation alongside an inseparable impurity was observed. (See the Supplementary Material for details) However, when isolated compound **6e** was treated with an aqueous basic solution, **7** could be isolated in good yields with high purity. This strategy was also attempted for the synthesis of VBX^7^ from **6d**, but was unsuccessful (See the Supplementary Material for details).

### 2.2 Reactivity investigation in *S*-vinylation of thiols

**SCHEME 2:**
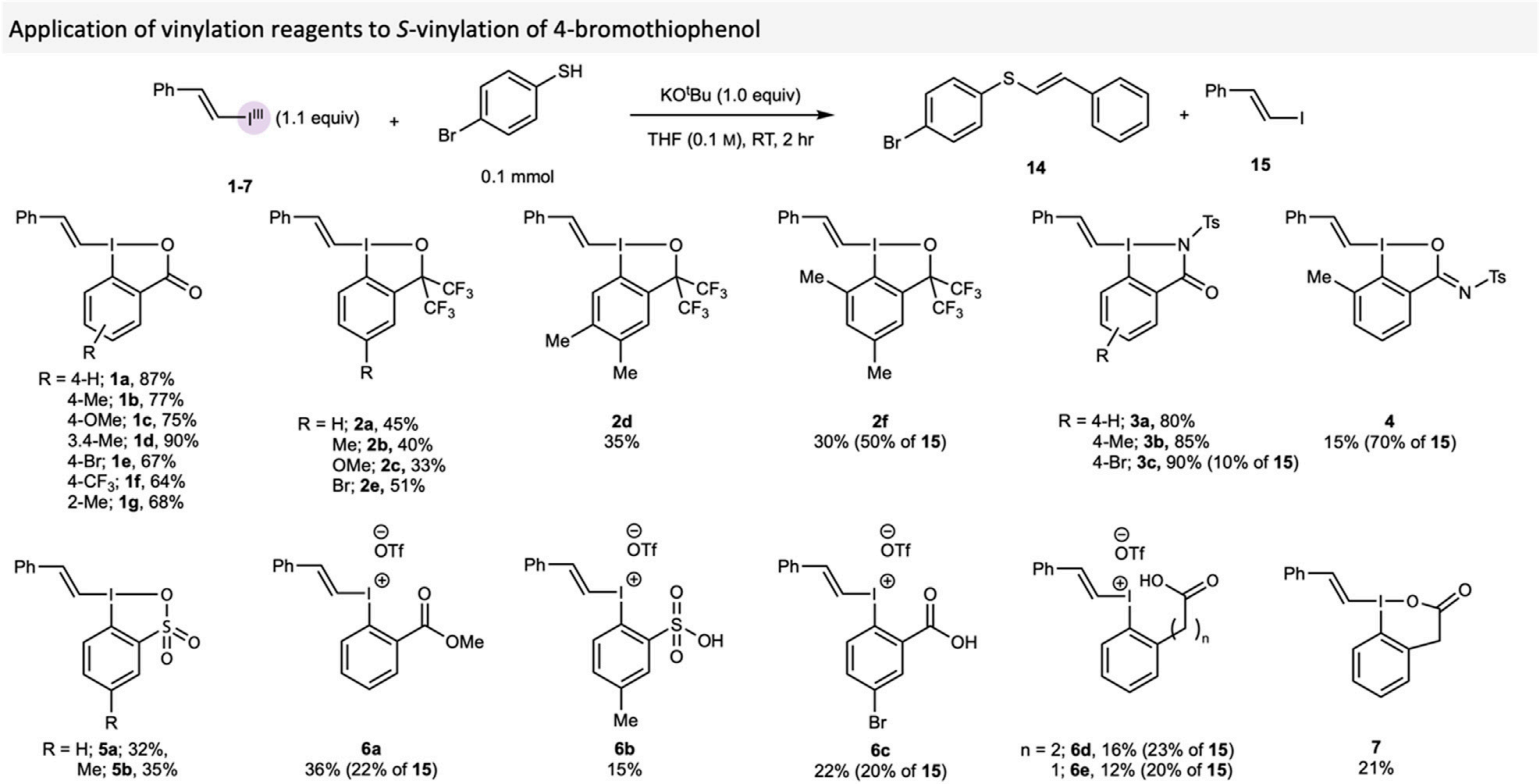
Comparison of vinylating reagents **1-7** in the *S*-vinylation of 4-bromothiophenol. Yields are NMR yields, which were calculated by using 1,3,5 trimethoxybenzene as internal standard.

The *S*-vinylation protocol developed by our group was used to evaluate the vinylating reagents, as this reaction had already proved sensitive to the VBX core structure. (Castoldi et al., 2020b). 4-Bromothiophenol was thus vinylated with reagents **1-7** to provide thioether **14** with vinyl iodide **15** sometimes formed as side-product (Scheme 2). The result obtained with the novel VBX reagent **1f** followed the trend in the original work, (Castoldi et al., 2020b), in which reagents with electron-donating groups (EDG) gave higher yields than those with electron-withdrawing groups (EWG). VBO reagents **2** performed markedly worse, with yields of **14** ranging from 25% to 51%. VBZ **3** behaved similarly to VBX, with **3c** providing the highest yield of 90%, whereas VBXI **4** and VBT **5** gave significantly lower yields, which could be due to solubility problems. *E/Z* ratios were recorded in of each of these reactions, but there was no observed trend with regards to the reagent used (See the Supplementary Material for details).

Additionally, the compounds with *ortho*-substituents performed worse than their counterparts (**1a** vs **1g**, **2a** vs **2f**). Due to the rapid decomposition of those reagents during isolation and analysis, we hypothesise that the *ortho*-substituents increase the reactivity of these compounds, especially since the majority of product in these reactions were the vinyl iodide **15**. Indeed, *ortho*-substitution in hypervalent iodine compounds has earlier been reported to cause considerable reactivity changes. (Guilbault and Legault, 2012; Malmgren et al., 2013; Abazid and Nachtsheim, 2020). Additionally, VBXI **4** produced a very poor yield of **14** in comparison to its analogues VBZ **3**, possibly due to its BXI core. Similar to what was reported with acyclic vinyliodonium salts in the original *S*-vinylation paper, (Castoldi et al., 2020b), reagents **6** provided little product, with **15** again being the major product in these reactions. VBX^6^ provided a much lower yield than VBX, indicating the importance of the 5-membered ring for the application of this reagent.

### 2.3 X-ray crystallography analysis

**FIGURE 2:**
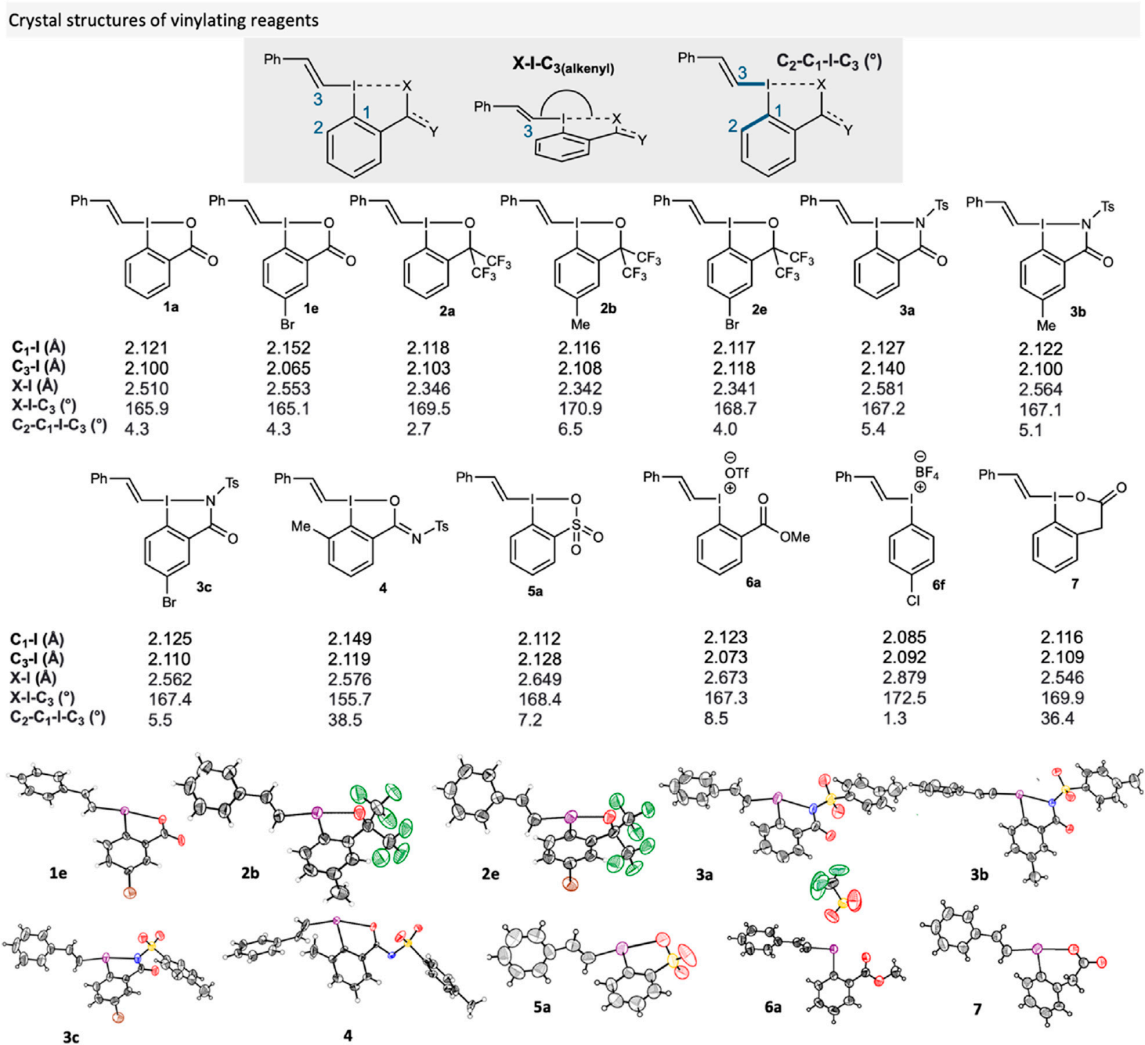
Novel crystal structures of reagents **1**-**7** compared to structures in the literature (**1a** (Stridfeldt et al., 2016), **2a** (Pisella et al., 2020) and **6f** (Clegg and Harrington, 1999)).

To evaluate how the core substituents and side-arms influenced the structure, we collected single crystal X-ray diffraction data on selected compounds (Figure 2). Much of the crystal structure data in the literature focusses on vinyliodonium salts, (Hinkle and McDonald, 2002; Ochiai et al., 2007; Yoshimura et al., 2021), and varied substitution patterns on the vinyl groups of VBO (Wu et al., 2016; Wu J. et al., 2017; Wu et al., 2019; Ding et al., 2020; Pisella et al., 2020; Chai et al., 2021; Laskar et al., 2021; Wu et al., 2023) and VBX. (Stridfeldt et al., 2016; Caramenti et al., 2019; Tessier et al., 2019). On the other hand, there appears to be no crystal structure investigations on the effect of cyclic vinylation reagents with different side-arms and core-substituents, as well as non-covalent interactions in *ortho*-substituted iodonium salts, on reaction outcome.

We evaluated the effect of the side-arm by comparison of core-unsubstituted VBX **1a**, VBO **2a**, VBZ **3a** and VBT **5a**, which showed very similar C_1_-I bond lengths, 2.127, 2.118, 2.127 and 2.112 Å respectively. The same is also true for C_3_-I bond lengths, as they measured at 2.100, 2.103, 2.140 and 2.128 Å respectively. However, X-I bond lengths were drastically different. **1a** and **3a** were somewhat similar with bond lengths of 2.510 and 2.581 Å respectively, whilst **2a** had a shorter bond length (2.346 Å) and **5a** has a longer bond length (2.649 Å). This is perhaps indicative of the increased *trans effect* caused by this functional group. (Ochiai et al., 2006). Additionally, measured X-I-C_3_ bond angles showed that all compounds expressed a T-shaped conformation with **1a** having the smallest angle, 165.9°, and VBO **2b** the largest, 170.9°. The X-ray crystal structure of VBX^6^ **7** showed a strained 6-membered ring in the side-arm, with similar bond lengths and hypervalent bond angle with VBX **1a**. However, **7** has a C_2_-C_1_-I-C_3_ bond angle of 36.4°, which is far higher than that of **1a** (4.3°).

To ascertain whether core-substituents made a measurable difference on any structural properties, several analogues of each class of vinylating reagent were also crystallised. Generally, the same trends followed within each class of compounds. Interestingly, the crystallographic data of *ortho*-substituted VBXI **4** showed a markedly lower X-I-C_3_ bond angle of 155.7°, which is the lowest angle of any hypervalent iodine vinylating reagent in the literature. Additionally, **4** has a C_2_-C_1_-I-C_3_ bond angle of 38.5°, which is far from the more idealised angle of 1°–8° for the other compounds. These two measurements show that the *ortho*-methyl substituent induces sufficient steric strain to disrupt the hypervalent bond and ultimately leads to the formation of an I-O bond, as opposed to the I-N bond found in VBZ **3**. We hypothesise that this key difference contributes to the reagent’s poor reactivity under the *S*-vinylation conditions. Furthermore, this characteristic likely contributes to their unstable and over-reactive nature as seen in Scheme 2. Interestingly, iodonium salt **6a** had similar C_1_-I (2.123 Å) and C_3_-I (2.073 Å) bond lengths to other compounds. Though, it has a much longer X-I bond length (2.673 Å), which is unsurprising considering the methyl ester ligand is not covalently bound, but it is markedly smaller than the X-I bond length of **6f** (The crystal structure data of 6f is included as comparison to 6a; we did not use 6f in other parts of the study) (2.879 Å). This shows that whilst not having a covalently bound group will affect the structural properties, a much more significant effect will be observed when non-ligating substituents are used in the *ortho* position to the iodine.

**FIGURE 3:**
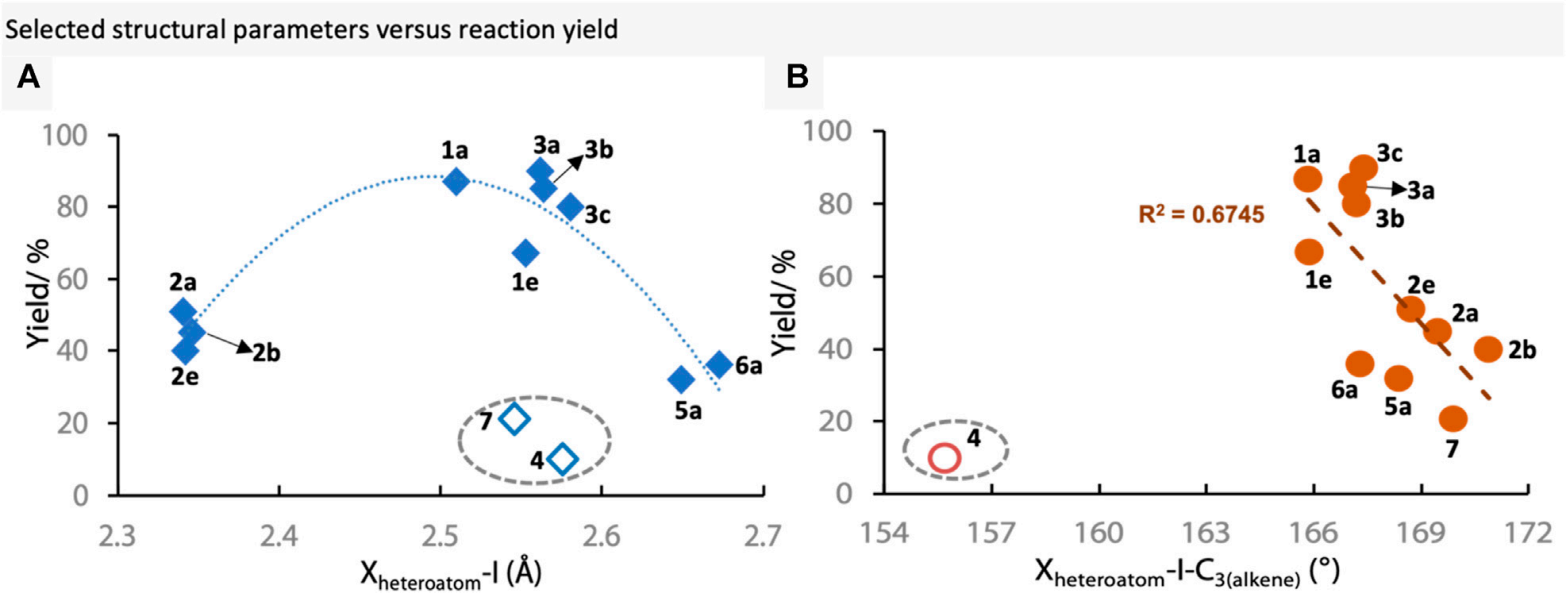
Selected structural parameters vs reaction outcome. Circled points are outliers. **(A)** X-I bond lengths vs reaction outcome **(B)** X-I-C_3_ bond angles vs reaction yield.

Next, the possible correlation between structural parameters and reaction outcome was investigated (Figure 3). Firstly, the X-I bond lengths were plotted against the yield of *S*-vinylation, showing an upward slope from 2.341 Å (**2e**) to 2.581 Å (**3a**), which was proceeded by a downward slope towards 2.673 Å of **6a** (ester-bound iodonium) (Figure 3A). Interestingly**, 4** and **7** were outliers to this trend (circled, hollow diamond). Thus, reagents with X-I bond lengths of ∼2.55 Å represent a “sweet-spot” under these reaction conditions. Then the hypervalent bond angles (X-I-C_3_) were plotted against reaction yields (Figure 3B). Within this parameter, it was observed that the higher the angle, and thus closer to the idealised 180°, the worse the reagent performed, with a peak of ∼165°. Again, reagent **4** was an outlier in this trend. These results show that there is indeed a link between these two structural parameters and the reaction yield, and that both electronic and steric factors influence the reaction outcome.

### 2.4 Reduction potential analysis

**FIGURE 4:**
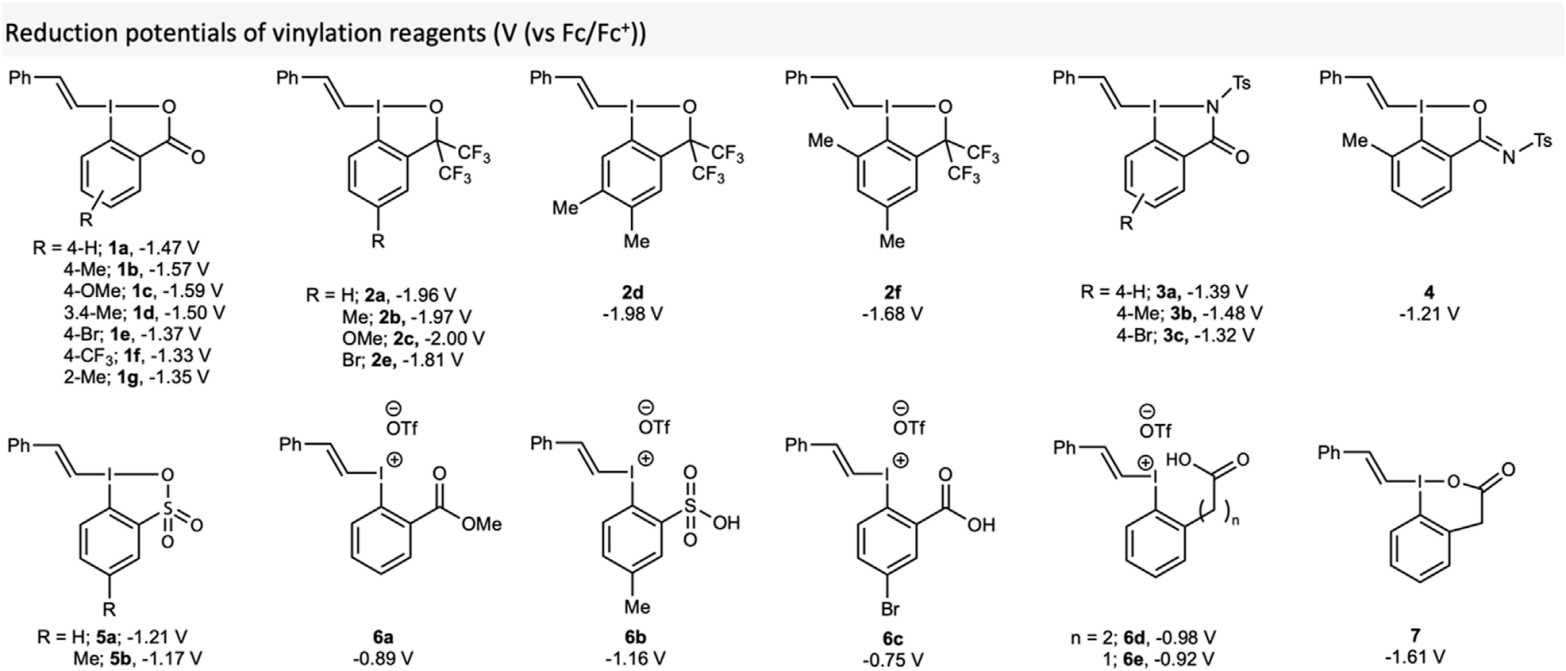
Recorded reduction potentials (V (vs Fc/Fc^+^)) of reagents used in this study.

We also wanted to investigate how the reduction potentials of the reagents were affected by substituents of the aromatic ring core and the side-arm (Figure 4). Whilst there have been some reports of redox potentials of hypervalent iodine reagents in the literature, (Kokkinidis et al., 1989; Kokkinidis et al., 1991; Choi et al., 2015; Le Vaillant and Waser, 2017; De et al., 2019; Ramkumar et al., 2023), and even a computational study, (Radzhabov et al., 2020), there is currently no data on vinylation reagents and certainly no quantitative studies linking this parameter to reaction outcome.

To begin, we measured the potentials of each of the reagent used in this study. The higher the reduction potential (closer the value to 0 V (vs Fc/Fc^+^)), the more easily the reagent can be reduced. It was found that there was a tight range of reduction potentials within each class of reagent. VBX ranged from −1.33 V (**1f**) to −1.59 V (**1c**), which matches that EWG should make the reagent easier to reduce. Considering its strong likeness to **1a**, VBX^6^ **7** had a very different potential of −1.61 V. Whilst VBZ **3a-c** had slightly higher potentials between −1.32 and −1.48 V, VBXI **4** (−1.21 V) and VBT **5** (−1.21 and −1.17 V) had lower potentials. The lowest reduction potentials were measured for VBO **2**, ranging from **2c** (−1.98 V) to **2f** (−1.68 V). As controls, the potentials of some vinyliodonium salts **6** were measured. Direct comparison of the uncyclized and cyclised analogues (**6c** vs **1e** and **6e** vs **6**) showed that the iodonium salts were indeed much easier to reduce. This was, however, not the case for **6b** (−1.16 V), which was very similar to its cyclised VBT counterpart **5b** (−1.17 V).

**FIGURE 5:**
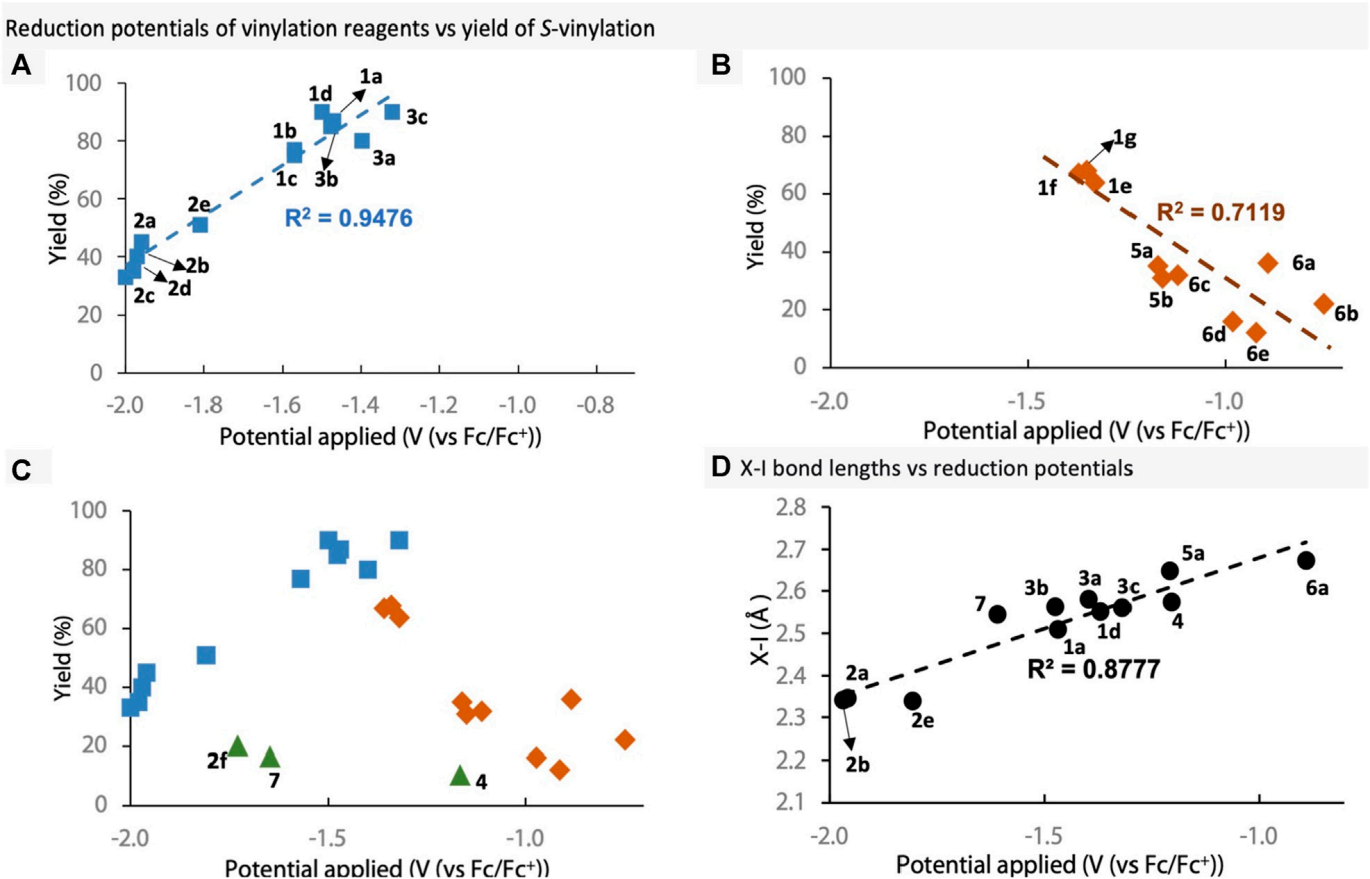
Reduction potentials of selected reagents plotted against *S*-vinylation. **(A)** Plot of **1a–1d**, **2a–2e** and **3**. **(B)** Plot of **1e–1g, 5** and **6**. **(C)** Overlay of plots **(A, B)**, including the outlier compounds **2f, 4** and **7**. **(D)** Plot of X-I bond lengths vs reduction potentials.

Next, to ascertain whether there is a relationship between reduction potentials of the vinylation reagents and their yield in *S*-vinylation, the two were plotted against each of other (Figure 5). Firstly, we plotted the VBO, VBZ and only the EDG-substituted VBX reagents, as EWG-substituted VBX performed poorly in *S*-vinylation (Figure 5A). Reagents with lower reduction potentials were found to have a positive effect on the reaction yield. Secondly, we plotted the EWG-substituted VBX reagents, VBT reagents and iodonium salts (Figure 5B). In this case, lower reduction potential resulted in lowered reaction yield together with increased levels of vinyl iodide (see data in Scheme 2). This is interesting because the less reactive reagents (e.g., VBO) provided lower reaction yields, whereas the more reactive reagents (e.g., iodonium salts) gave low yields and more vinyl iodide. Furthermore, the results suggest that a reagent with a reduction potential between −1.3 and −1.5 V, represents the peak of this reaction, with potentials on either side being ultimately detrimental for the reagents. However, this could be due to the reaction itself being optimised on VBX **1a**. Curiously however, if both plots are overlayed with the previously excluded reagents, it becomes clear that there are some outliers to this trend (green triangles, Figure 5C). These are VBX^6^ **7** and *ortho*-substituted VBO **2f** and VBXI **4**, and the results might reflect the lack of idealised T-shaped conformation or conjugation in those structures. Clearly the reduction potential does not account for the change in T-shaped conformation and steric factors very well. Overall, it appears that the reduction potentials can be a good signifier for the efficiency of the vinylation reagent in the *S*-vinylation under these reaction conditions, but further reaction optimisation could potentially alter the outcome. Finally, a fine correlation was observed between X-I bond lengths and reduction potentials (Figure 5D), signifying that X-I bond lengths could affect the reduction potentials greatly.

## 3 Materials and methods

For general experimental and instrumental methods, synthetic procedures, and full compound characterization, see the Supplementary Material.

## 4 Conclusion

In conclusion, the synthesis of novel hypervalent iodine-based vinylation reagents has been reported, including the new compound classes VBZ, VBXI, VBT and VBX^6^. These reagents were evaluated in the *S*-vinylation of 4-bromothiophenol and VBZ performed similarly to VBX, whilst VBO, VBXI, VBT and iodonium salts proved inferior. Crystal structures of selected reagents were measured, as well as electronic potentials of all the reagents. Crystal structure data showed that there was a correlation between certain parameters and reaction outcome, and *ortho*-substituents were found to perturb the reagent’s structure and hence destabilise it. Additionally, reduction potentials were plotted against reaction outcome, which showed a sweet spot of about −1.4 V, when ignoring certain outliers in the study. Additionally, there was a correlation between reduction potentials and X-I bond length. We believe that further investigations of properties vs reaction outcome could result in a method for predicting reaction outcome with hypervalent iodine reagents.

## Data Availability

The original contributions presented in the study are included in the article/Supplementary Material, further inquiries can be directed to the corresponding author.
