# Supplementary material for: Structure-reactivity analysis of novel hypervalent iodine reagents in S-vinylation of thiols
Source: Front Chem. 2024 Feb 29;12:1376948. doi: 10.3389/fchem.2024.1376948 (PMC10937425; doi:10.3389/fchem.2024.1376948)
Supplement: Supplementary file 1 [file DataSheet2.PDF]

Structure-reactivity analysis of novel hypervalent iodine reagents in S-vinylation of thiols

Sayad Doobary[1], Ester Maria Di Tommaso[1], Alexandru Postole[1], A. Ken Inge[2], Berit Olofsson[1*]

[1] Dept. of Organic Chemistry, Stockholm University, 106 91 Stockholm, Sweden
[2] Dept of Materials and Environmental Chemistry, Stockholm University, 106 91 Stockholm, Sweden

Table of contents

1 General experimental ___ ***2***

2 Novel compounds contents table ___ ***3***

3 Synthesis of starting materials ___ ***6***

3.1 General procedure 1 (GP 1): Synthesis of hydroxybenziodoxoles 9 ___ **6**

3.2 General procedure 2 (GP 2): Sandmeyer reaction to form iodo alcohols 10 ___ **7**

3.3 General procedure 3 (GP 3): Oxidative chlorination and hydrolysis to form 11 ___ **9**

3.4 General procedure 4 (GP 4): Synthesis of 2-iodo benzamides 12a-d ___ **11**

3.5 General procedure 5 (GP 5): Synthesis of 2-iodo sulfonic acids 12e, f ___ **13**

4 Synthesis of products 1-7 ___ ***15***

4.1 Synthesis of VBX reagents 1 ___ **15**

4.2 General procedure 6 (GP 6): Synthesis of VBO reagents 2 ___ **16**

4.3 General procedure 7 (GP 7): One-pot synthesis of VBZ derivatives 3a–c ___ **18**

4.4 (*E*)-4-methyl-*N*-((*Z*)-7-methyl-1-((*E*)-styryl)-1λ^3 -benzo[*d*][1,2]iodaoxol-3(1*H*)-ylidene)benzenesulfonamide (4) ___ **20**

4.5 General procedure 8 (GP 8): one-pot synthesis of VBS derivatives 5a,b ___ **20**

4.6 General procedure 9 (GP 9): one-pot synthesis of iodonium salts 6 ___ **21**

5 Failed reactions ___ ***25***

6 S-vinylation of bromothiophenol ___ ***27***

6.1 Vinylation to yield (*E*)-(4-bromophenyl)(styryl)sulfane (14) ___ **27**

7 Crystal structure determination ___ ***29***

8 Electrochemical analysis ___ ***40***

8.1 Electrode potential setup ___ **40**

8.2 Cyclic voltametric plots ___ **41**

8.3 Measured reduction potentials ___ **53**

9 NMR of substrates ___ ***54***

10 NMR of products ___ ***74***

11 References ___ ***104***

1 General experimental

The used reagents were bought from commercial suppliers and used as received, unless noted otherwise. Moisture and air sensitive reactions were carried out under argon or nitrogen environment using standard Schlenk techniques. Reactions performed above the boiling point of the solvent(s) were performed in pressure-stable microwave vials or using a standard condenser. Solvents were obtained as P.A. grade and dried using a VAC solvent purification system. *m*-CPBA (Aldrich, 77 % active oxidant) was dried under vacuum for 4 hours, after which the amount of active oxidant was determined through an iodometric titration.[1] Thin layer chromatography (TLC) was performed using TLC Silica gel 60 F254 plates (Merck) and visualized using UV-light and all TLC plates were stained with potassium permanganate stain. Purification of the products was conducted by flash column chromatography on SiO_2 purchased from Aldrich (technical grade, 60 Å pore size, 230-400 mes). Melting points were measured using a STUART SMP3 and are reported uncorrected. NMR measurements were conducted using a 400 MHz Bruker AVANCE II with a BBO probe at 298 K unless otherwise stated. Chemical shifts (δ) are reported in parts per million (ppm) and referenced $CDCl_3$ (^{1}H: 7.26 ppm; ^{13}C: 77.0 ppm), DMSO-d_6 (^{1}H: 2.50 ppm; ^{13}C: 39.5 ppm) or MeOD-d_6 (^{1}H: 3.31 ppm; ^{13}C: 49.0 ppm). Coupling constants (*J)* are given in Hertz (Hz) and refer to apparent multiplicities (s = singlet, d = doublet, t = triplet, q = quartet, pent = pentet, m = multiplet, br = broad signal, and combinations thereof for example, dd = doublet of doublets.) HRMS spectra were measured on a Bruker microTOF with electron spray ionization (ESI). Values of $E_{p/2}$ were measured using the program EchoLab.

2 Novel compounds contents table

9b

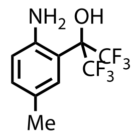

Characterization

9c

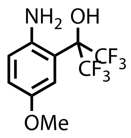

Characterization

9d

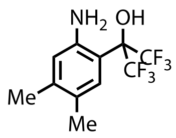

Characterization
Spectra

9e

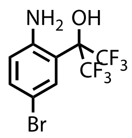

Characterization
Spectra

9f

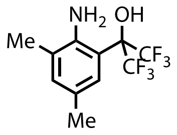

Characterization
Spectra

10b

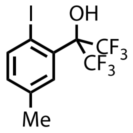

Characterization

10c

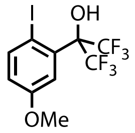

Characterization

10d

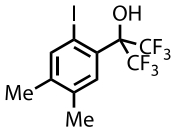

Characterization
Spectra

10e

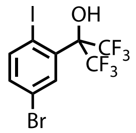

Characterization
Spectra

10f

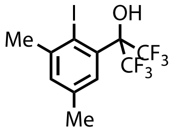

Characterization
Spectra

11a

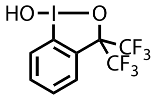

Characterization

11b

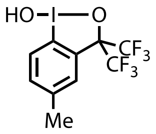

Characterization

11c

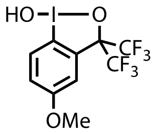

Characterization
Spectra

11d

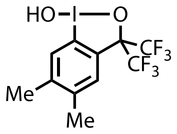

Characterization
Spectra

11e

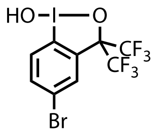

Characterization
Spectra

11f

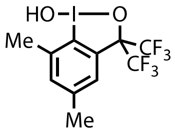

Characterization
Spectra

12a

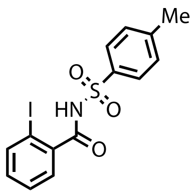

Characterization

12b

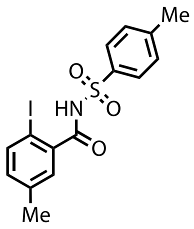

Characterization

12c

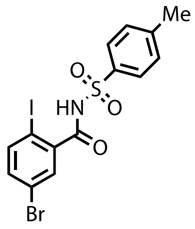

Characterization

12d

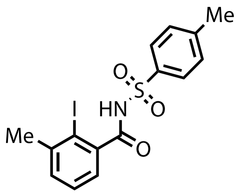

Characterization

12e

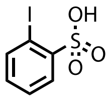

[Characterization]

12f

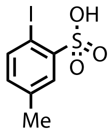

[Characterization]

1a

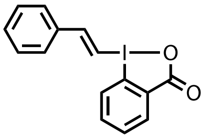

[Characterization]

1b

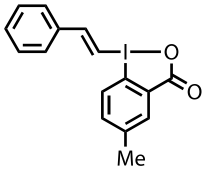

[Characterization]

1c

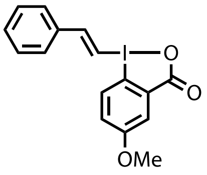

[Characterization]

1d

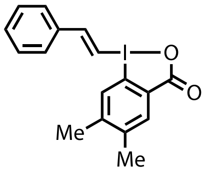

[Characterization]

1e

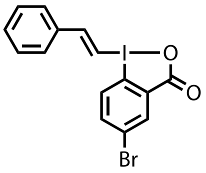

[Characterization]

1f

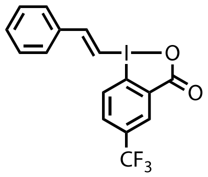

[Characterization]
[Spectra]

1g

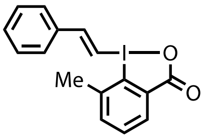

[Characterization]

2a

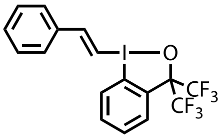

[Characterization]

2b

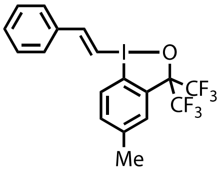

[Characterization]
[Spectra]

2c

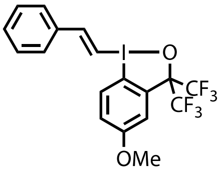

[Characterization]
[Spectra]

2d

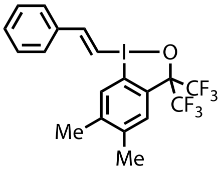

[Characterization]
[Spectra]

2e

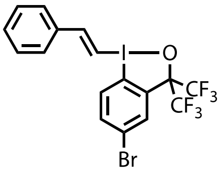

[Characterization]
[Spectra]

2f

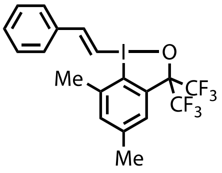

[Characterization]
[Spectra]

3a

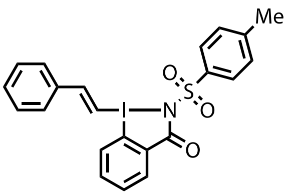

[Characterization]
[Spectra]

3b

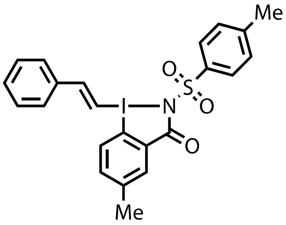

Characterization
Spectra

3c

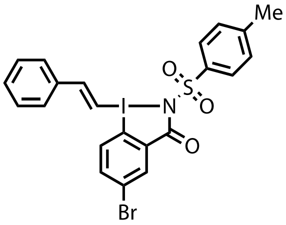

Characterization
Spectra

4

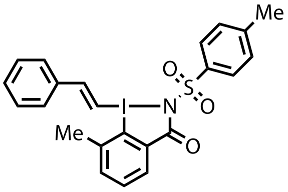

Characterization
Spectra

5a

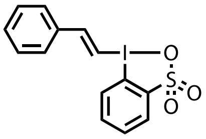

Characterization
Spectra

5b

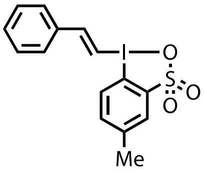

Characterization
Spectra

6a

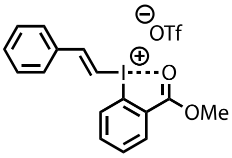

Characterization
Spectra

6b

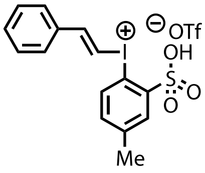

Characterization
Spectra

6c

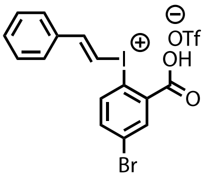

Characterization
Spectra

6d

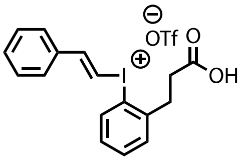

Characterization
Spectra

6e

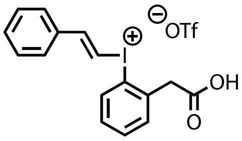

Characterization
Spectra

7

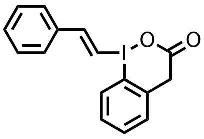

Characterization
Spectra

14

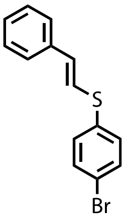

Characterization

3 Synthesis of starting materials

3.1 General procedure 1 (GP 1): Synthesis of hydroxybenziodoxoles 9

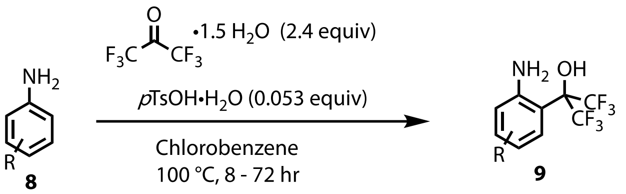

Following a previously published procedure,[2] to an oven-dried pressure-stable microwave vial, under inert atmosphere, was added the respective aniline derivative (4.0 – 15.0 mmol, 1.0 equiv), *p*TsOH·H_2O (0.053 equiv) followed by the addition of chlorobenzene (5.2 M). Then, the vial was sealed and heated at 100 °C followed by dropwise addition of hexafluoroacetone sesquihydrate (2.4 equiv) over 5 minutes. The reaction mixture was subsequently stirred at the indicated temperature for the notified time. The residue was dissolved in minimal amount of chloroform and left in the freezer overnight. The remaining solvent was then carefully removed with a syringe leaving the precipitated product behind, which was lastly dried under vacuum to produce **8.**

3.1.1 2-(2-Amino-5-methylphenyl)-1,1,1,3,3,3-hexafluoropropan-2-ol (9b)

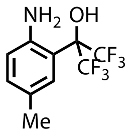

Synthesized using **GP 1** from 4-methyllaniline (1.07 g, 10.0 mmol) using 24 h reaction time. After reaction completion, the aqueous phase was extracted with ethyl acetate (3 x 100 mL). The combined organic layers were subsequently dried over anhydrous $MgSO_4$ followed by evaporation. Finally, the residue was washed with *n*-pentane (5 x 10 mL) to afford **9b** as a slightly rose solid (1.322 g, 4.84 mmol, 48 %);
δ_H (400 MHz, $CDCl_3$) 7.37 (1H, s), 7.16 (1H, dd, J = 8.0, 1.2 Hz), 6.96 (1H, d, J = 8.0 Hz) 3.50 (1H, s), 2.35 (3H, s);
δ_F (377 MHz, $CDCl_3$) -75.13;
δ_C (101 MHz, $CDCl_3$) 138.4, 135.5, 131.3, 129.2, 127.4, 125.4 (q, J = 288.6 Hz), 123.0, 80.0 (pent, J = 30.4 Hz), 21.2.
This data is consistent with literature precedent.[3]

3.1.2 2-(2-Amino-5-methoxyphenyl)-1,1,1,3,3,3-hexafluoropropan-2-ol (9c)

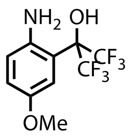

Synthesized using **GP 1** from 4-methoxyaniline (0.616 mg, 5.00 mmol) using 18 h reaction time and hexafluoroacetone sesquihydrate (1.5 equiv) to produce **9c** as a slightly purple solid (1.235 g, 4.27 mmol, 85 %);
δ_H (400 MHz, $CDCl_3$) 7.14 (1H, s), 7.03 (1H, d, J = 8.7 Hz), 6.88 (1H, dd, J = 8.7, 2.7 Hz), 5.79 (3H, s), 3.76 (3H, s);
δ_F (377 MHz, $CDCl_3$) -75.30;
δ_C (101 MHz, $CDCl_3$) 157.1, 133.5, 129.7, 125.1, 123.3 (q, J = 289.1 Hz), 115.5, 144.4, 80.1 (pent, J = 30.1 Hz), 55.7.
This data is consistent with literature precedent.[4]

3.1.3 2-(2-Amino-4,5-dimethylphenyl)-1,1,1,3,3,3-hexafluoropropan-2-ol (9d)

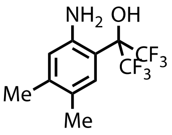

Synthesized using **GP 1** from 3,4-dimethylaniline (0.490 g, 4.00 mmol) using 8 h reaction time to produce **9d** as a slightly purple solid (1.030 g, 3.60 mmol, 90 %);
δ_H (400 MHz, $CDCl_3$) 7.31 (1H, s), 6.85 (1H, s), 2.25 (3H, s), 2.23 (3H, s);
δ_F (377 MHz, $CDCl_3$) -75.30;
δ_C (101 MHz, $CDCl_3$) 139.6, 138.9, 134.0, 129.4 (pent, *J* = 29.5 Hz), 128.6, 123.5 (q, *J* = 288.0 Hz), 120.4, 79.9 (pent, *J* = 30.0 Hz), 19.6, 19.5;
HRMS (ESI) calculated for $C_{11}H_{12}F_6NO^+$ ($M+H^+$): 288.0818; found: 288.0825;
MP 115.0-118.0 °C

3.1.4 2-(2-Amino-5-bromophenyl)-1,1,1,3,3,3-hexafluoropropan-2-ol (9e)

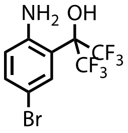

Synthesized using **GP 1** from 4-bromoaniline (2.303 g, 10.71 mmol) using 3 days reaction time to produce **9e** as a slightly purple solid (2.99 g, 8.87 mmol, 83 %);
δ_H (400 MHz, $CDCl_3$) 7.66 (1H, s), 7.45 (1H, dd, *J* = 8.5, 2.1 Hz), 6.89 (1H, d, *J* = 8.5 Hz), 5.34 (3H, s);
δ_F (377 MHz, $CDCl_3$) -75.02;
δ_C (101 MHz, $CDCl_3$) 141.6, 133.8, 131.6 (pent, *J* = 3.1 Hz), 127.2, 123.2 (q, *J* = 289.1 Hz), 122.6, 117.0, 79.7 (pent, *J* = 30.0 Hz).
HRMS (ESI) calculated for $C_9H_7BrF_6NO^+$ ($M+H^+$): 337.9610; found: 337.9615.
MP 129.0 - 118.1 °C.

3.1.5 2-(2-Amino-3,5-dimethylphenyl)-1,1,1,3,3,3-hexafluoropropan-2-ol (9f)

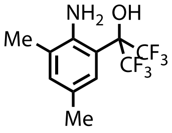

Synthesized using **GP 1** from 2,4-dimethylaniline (1.818 g, 15.00 mmol) using 48 h reaction time to produce **9f** as a slightly purple solid (3.231 g, 11.25 mmol, 75 %);
δ_H (400 MHz, $CDCl_3$) 7.26 (1H, s), 7.12 (1H, s), 2.32 (3H, s), 2.29 (3H, s);
δ_F (377 MHz, $CDCl_3$) -75.10;
δ_C (101 MHz, $CDCl_3$); 137.2, 135.0, 134.3, 132.8, 127.1 (pent, *J* = 2.69 Hz), 123.7, 123.5 (q, *J* = 287.6 Hz), 80.3 (pent, *J* = 29.7 Hz), 77.5, 77.2, 76.8, 21.2, 18.2;
HRMS (ESI) calculated for $C_{11}H_{11}F_6ONNa$ ($M+Na^+$): 310.0637; found: 310.0639;
MP 114.1 – 117.2 °C.

3.2 General procedure 2 (GP 2): Sandmeyer reaction to form iodo alcohols 10

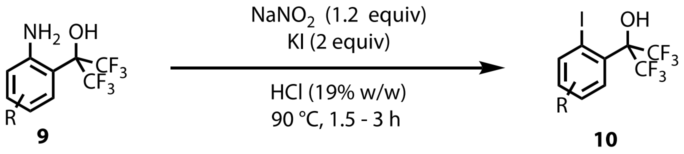

Following a previously published procedure,[5] the *o*-aminobenzyl alcohol **9** (3.90 – 13.95 mmol, 1.0 equiv) and 6 M HCl (0.5 M) were added to a round bottom flask. After cooling down

the suspension to 0 °C, a solution of 2.4 M $NaNO_2$ in water (1.2 equiv), was added dropwise while stirring vigorously. Then, the reaction mixture was stirred for 30 min at 0 °C. Afterwards, a solution of potassium iodide in water (3.9 M, 2.0 equiv), was added dropwise normally resulting in a brown solution. The reaction was subsequently heated at 90 °C for 1.5 h. After cooling down to room temperature, the reaction mixture was quenched with saturated sodium thiosulfate solution. The resulting bright yellow mixture was transferred into a separating funnel with 100 mL EtOAc. Dilution of both layers helped to avoid emulsions in the separation. Afterwards, the aqueous phase was extracted with EtOAc (3 x 100 mL) and the combined organic layers were dried over anhydrous $MgSO_4$, filtered and concentrated. Thereupon, no further purification of the product **10** was necessary.

3.2.1 1,1,1,3,3,3-Hexafluoro-2-(2-iodo-5-methylphenyl)propan-2-ol (10b)

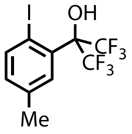

Synthesized using **GP 2** from **9b** (1.323 g, 4.84 mmol) using 1.5 h reaction time to produce **10b** as a green solid (1.506 g, 3.90 mmol, 81 %);
δ_H (400 MHz, $CDCl_3$) 7.97 (1H, d, J = 8.0 Hz), 7.42 (1H, s), 6.93 (1H, dd, J = 8.0, 1.5 Hz), 4.27 (1H, s), 2.35 (3H, s);
δ_F (377 MHz, $CDCl_3$) -73.40;
δ_C (101 MHz, $CDCl_3$) 144.5, 138.4, 132.7, 130.9, 129.6, 122.7 (q, J = 290.5 Hz), 86.6, 78.9 (pent, J = 30 Hz), 21.3;

This data is consistent with literature precedent.[6]

3.2.2 1,1,1,3,3,3-Hexafluoro-2-(2-iodo-5-methoxyphenyl)propan-2-ol (10c)

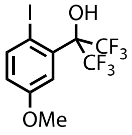

Synthesized using **GP 2** from **9c** (1.300 g, 4.50 mmol) using 2 h reaction time to produce **10c** as a brown solid (1.259 g, 2.07 mmol, 46 %);
δ_H (400 MHz, $CDCl_3$) 7.97 (1H, d, J = 8.8 Hz), 7.20 (1H, d, J = 2.8 Hz), 6.71 (1H, dd, J = 8.8, 2.8 Hz), 4.36 (1H, s), 3.81 (3H, s, 3H);
δ_F (377 MHz, $CDCl_3$) -73.49;
δ_C (101 MHz, $CDCl_3$) 159.3, 145.2, 130.7, 122.6 (q, J = 290.4 Hz), 117.5 (pent, J = 3.2 Hz), 117.1, 117.1, 78.1, 55.5.

This data is consistent with literature precedent.[7]

3.2.3 1,1,1,3,3,3-Hexafluoro-2-(2-iodo-4,5-dimethylphenyl)propan-2-ol (10d)

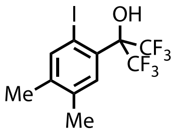

Synthesized using **GP 2** from **9d** (0.908 g, 3.162 mmol) using 1.5 hr reaction time to produce **10d** as a brown solid (1.050 g, 6.324 mmol, 87 %).;
δ_H (400 MHz, $CDCl_3$) 7.87 (1H, s), 7.35 (1H, s), 4.28 (1H, s), 2.25 (3H, s), 2.22 (3H, s);
δ_F (377 MHz, $CDCl_3$) -73.52;
δ_C (101 MHz, $CDCl_3$) 145.2, 141.1, 137.2, 131.0 (pent, J = 2.6 Hz), 127.0, 122.6 (q, J = 288.2 Hz), 87.0, 78.8 (pent, J = 30.3 Hz), 19.9, 19.0;
HRMS (ESI) $C_{11}H_9F_6IONa^+$ ($M+Na^+$): 420.9494; found: 420.9490;
MP 112.1 – 115.1 °C.

3.2.4 2-(5-Bromo-2-iodophenyl)-1,1,1,3,3,3-hexafluoropropan-2-ol (10e)

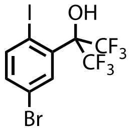

Synthesized using **GP 2** from **9e** (2.943 g, 8.71 mmol) using 3 h reaction time to produce **10e** as a dark green solid (3.742 g, 8.32 mmol, 96 %);

δ_H (400 MHz, CDCl$_3$) 7.98 (1H, d, *J* = 8.5 Hz), 7.72 (1H, s), 7.25 (1H, dd, *J* = 8.5, 2.1 Hz), 4.12 (1H, s);

δ_F (377 MHz, CDCl$_3$) -73.45;

δ_C (101 MHz, CDCl$_3$) 146.0, 134.8, 133.0 (pent, *J* = 2.3 Hz), 131.7, 122.7, 122.4 (q, *J* = 290.3 Hz), 88.7, 78.4 (pent, *J* = 30.3 Hz);

HRMS (ESI) calculated for $C_9H_3{}^{79}BrF_6IO$ (M-H$^+$): 446.8311; found: 446.8322;

MP 115.0 – 118 °C.

3.2.5 2-(5-Bromo-2-iodophenyl)-1,1,1,3,3,3-hexafluoropropan-2-ol (10f)

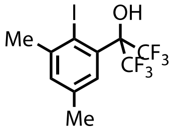

Synthesized using **GP 2** from **9f** (3.200 g, 11.14 mmol) using 2 h reaction time to produce **10f** as a yellow solid (4.906 g, 8.133 mmol, 73 %).

δ_H (400 MHz, CDCl$_3$) 7.26 (1H, s), 7.24 (1H, s), 4.95 (1H, s), 2.53 (3H, s), 2.33 (3H, s);

δ_F (377 MHz, CDCl$_3$) -72.74;

δ_C (101 MHz, CDCl$_3$) 144.7, 138.0, 132.9, 130.1, 128.8 (pent, *J* = 2.3 Hz), 122.9 (q, *J* = 292.4 Hz), 95.2, 80.0 (t, *J* = 29.6 Hz), 32.0, 21.0;

HRMS (ESI) calculated for $C_{11}H_9F_6IONa$ (M+Na$^+$): 420.9494; found: 420.9440;

MP 113.5 – 115.4 °C.

3.3 General procedure 3 (GP 3): Oxidative chlorination and hydrolysis to form 11

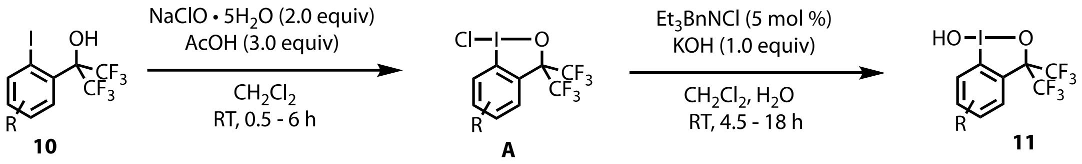

Following a previously published procedure,[8] the 2-iodobis(trifluoromethyl)benzyl alcohol **10** (2.06 – 7.12 mmol, 1.0 equiv) was dissolved in CH_2Cl_2 (0.8 M) and then cooled to 0 °C. To this solution was added, AcOH (3.0 equiv), followed by NaClO · 5 H_2O (2.0 equiv). A colour change from orange to yellow is typically observed. Then, the reaction mixture was stirred for 30 min at room temperature. After additional dilution with CH_2Cl_2 (10 - 50 mL), the solution was filtered over celite eluting with CH_2Cl_2 (50 mL). Finally, the solvent was evaporated to receive **A** as a normally yellow/orange solid. No further purification was necessary.

Following a previously published procedure,[9] crude product **A** (1.91 – 6.97 mmol, 1.0 equiv) was dissolved in CH_2Cl_2 (0.14 M). Afterwards, a solution of potassium hydroxide in water (0.12 M, 1.0 equiv) and then Et_3BnNCl (0.05 equiv) were added to the reaction mixture. The suspension was stirred for 4.5 h at room temperature. Afterwards, the aqueous phase was extracted with EtOAc (3 x 50 mL) and the combined organic layers were dried over anhydrous

$MgSO_4$, filtered and concentrated. After purification over a silica plug with EtOAc, the desired product **11** was obtained.

3.3.1 3,3-Bis(trifluoromethyl)- 1λ³-benzo[*d*][1,2]iodaoxol-1(3*H*)-ol (11a)

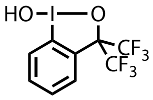

Prepared according to literature precedent.[10]

3.3.2 5-Methyl-3,3-bis(trifluoromethyl)- 1λ³-benzo[*d*][1,2]iodaoxol-1(3*H*)-ol (11b)

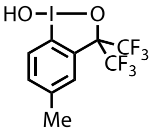

Synthesized using **GP 3** from **10b** (1.478 g, 3.82 mmol) using 0.5 h reaction time to produce a yellow solid (1.467 g, 3.51 mmol, 92 %). This was used directly in the next step.

Then, the aforementioned yellow solid (1.431 g, 3.42 mmol) was used in the hydrolysis step using 4.5 h reaction time to produce **11b** as an orange solid (1.249 g, 3.12 mmol, 92%, 85% over two steps);
δ_H (400 MHz, $CDCl_3$) 7.68 (1H, d, *J* = 8.6 Hz), 7.61 (1H, dd, *J* = 8.6, 1.1 Hz), 7.54 (1H, s), 2.55 (3H, s);
δ_F (377 MHz, $CDCl_3$) -75.98;
δ_C (101 MHz, $CDCl_3$) 142.0, 134.3, 131.7, 130.8, 126.3, 123.3 (q, *J* = 290.0 Hz), 112.3, 84.0 (pent, *J* = 30.0 Hz), 21.1.

This data is consistent with literature precedent.[11]

3.3.3 5-Methoxy-3,3-bis(trifluoromethyl)- 1λ³-benzo[*d*][1,2]iodaoxol-1(3)-ol (11c)

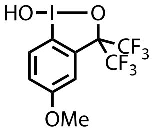

Synthesized using **GP 3** from **10c** (1.200 g, 3.00 mmol) using 2 h reaction time to produce an orange solid (0.895 g, 2.06 mmol, 69 %). This was used directly in the next step.

Then, the aforementioned orange solid (0.691 g, 1.66 mmol) was used in the hydrolysis step using 5 h reaction time to produce **11c** as an orange solid (0.595 g, 1.43 mmol, 90%, 62% over two steps);
δ_H (400 MHz, $CDCl_3$) 7.74 (1H, d, *J* = 9.0 Hz), 7.24 (1H, d, *J* = 2.7 Hz), 7.22 (1H, s), 3.88 (3H, s);
δ_F (377 MHz, $CDCl_3$) -76.01;
δ_C (101 MHz, $CDCl_3$) 162.4, 133.2, 127.3, 123.2 (q, *J* = 236.2 Hz), 119.1, 116.1, 104.2, 83.9 (pent, *J* = 29.3 Hz), 56.2;
HRMS (ESI) calculated for $C_{10}H_7F_6IO_2Na$ ($M+Na^+$): 438.9349; found: 438.9346;
MP 173.5 – 176.1 °C.

3.3.4 5,6-Dimethyl-3,3-bis(trifluoromethyl)- 1λ³-benzo[*d*][1,2]iodaoxol-1(3*H*)-ol (11d)

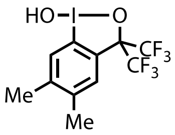

Synthesized using **GP 3** from **10d** (1.092 g, 2.74 mmol) using 0.5 h reaction time to produce an orange solid (0.967 g, 2.21 mmol, 81 %). This was used directly in the next step.

Then, the aforementioned orange solid (0.899 g, 2.08 mmol) was used in the hydrolysis step using 4.5 h reaction time to produce **11d** as an orange solid (0.752 g, 1.82 mmol, 87%, 74% over two steps);
δ_H (400 MHz, $CDCl_3$) 7.51 (1H, s), 7.48 (1H, s), 2.43 (3H, s), 2.39 (3H, s);
δ_F (377 MHz, $CDCl_3$) -75.98
δ_C (101 MHz, $CDCl_3$) 143.3, 140.6, 130.9, 129.1, 126.8, 123.5 (q, J = 290.8 Hz), 112.4, 83.9 (pent, J = 30.2 Hz), 20.6, 19.9;
HRMS (ESI) calculated for $C_{11}H_9F_6IO_2Na$ ($M+Na^+$): 436.9444; found: 436.9458;
MP 149.0 – 152.0 °C.

3.3.5 5-Bromo-3,3-bis(trifluoromethyl)- 1λ^3-benzo[*d*][1,2]iodaoxol-1(3*H*)-ol (11e)

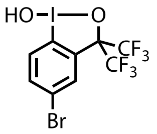

Synthesized using **GP 3** from **10e** (3.674 g, 8.18 mmol) using 6 h reaction time to produce an orange solid (3.44 g, 7.12 mmol, 87 %). This was used directly in the next step.
Then, the aforementioned orange solid (3.369 g, 6.97 mmol) was used in the hydrolysis step using 6 h reaction time to produce **11e** as an orange solid (2.03 g, 4.37 mmol, 63 %, 55% over two steps);
δ_H (400 MHz, $CDCl_3$) 8.15 (1H, dd, J = 8.8, 1.7 Hz), 7.88 (1H, d, J = 8.8 Hz), 7.73 (1H, s);
δ_F (377 MHz, $CDCl_3$) -75.18;
δ_C (101 MHz, $CDCl_3$) 136.2. 133.5, 130.9, 130.3, 124.6, 123.1 (q, J = 287.7 Hz), 117.0, 83.4 (t, J = 29.0 Hz);
HRMS (ESI) calculated for $C_9H_3BrF_6IO_2$ ($M-H^+$): 462.8260; found: 462.8248;
MP 242.0 – 245.0 °C.

3.3.6 5,7-Dimethyl-3,3-bis(trifluoromethyl)- 1λ^3-benzo[*d*][1,2]iodaoxol-1(3*H*)-ol (11f)

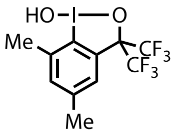

Synthesized using **GP 3** from **10f** (3.100 g, 7.69 mmol) using 0.5 h reaction time to produce an orange solid (2.860 g, 6.61 mmol, 85 %). This was used directly in the next step.
Then, the aforementioned orange solid (2.700 g, 6.24 mmol) was used in the hydrolysis step using 18 h reaction time to produce **11f** as an orange solid (2.467 g, 5.96 mmol, 95%, 81% over two steps);
δ_H (400 MHz, $CDCl_3$) 7.33 (1H, s), 7.29 (1H, s), 2.62 (3H, s), 2.46 (3H, s);
δ_F (377 MHz, $CDCl_3$) -74.96;
δ_C (101 MHz, $CDCl_3$) 141.7, 140.5, 138.1, 134.5, 128.2, 123.6 (q, J = 291.7 Hz), 113.8, 84.4 (t, J = 23.8 Hz), 23.2, 20.8;
HRMS (ESI) calculated for $C_{12}H_{11}F_6IO_2Na$ ($M-OH+MeO+Na^+$): 450.9600; found: 450.9599; NOTE: HRMS sample was prepared in MeOH;
MP 148.5 – 151.2 °C.

3.4 General procedure 4 (GP 4): Synthesis of 2-iodo benzamides 12a-d

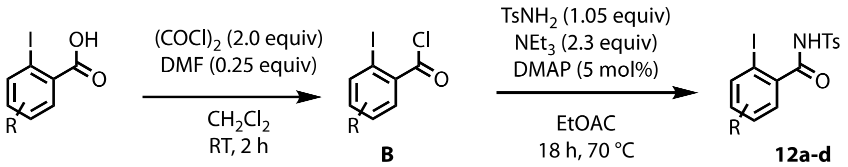

To a solution of 2-iodobenzoic acid (1.85 – 5 mmol, 1.0 equiv) in CH_2Cl_2 (0.25 M) was added oxalyl chloride (2.0 equiv) under an inert atmosphere. Then DMF (0.25 equiv) was added and the reaction mixture was allowed to stir for 2 h. The solvent was removed *in vacuo* and the crude compound **B** was used in the next step.

To the crude **B** was added EtOAc (0.16 M), *N*-tosylamide (1.05 equiv), DMAP (0.05 equiv). Whilst the mixture was stirring, triethylamine (2.3 equiv) was added and the reaction was heated to 70 °C for 18 h. After cooling down, 1 M HCl (50 mL) was then added and left to stir for 30 mins at room temperature. Then the aqueous phase was extracted with EtOAc (3 x 50 mL). After washing with Na_2SO_4, the mixture was filtered and then solvent was removed *in vacuo*. The substrate **12a-d** was isolated via column chromatography (Pentane:EtOAc).

3.4.1 2-Iodo-*N*-tosylbenzamide (12a)

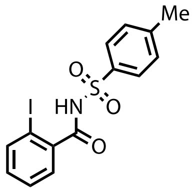

Synthesized using **GP 4** from 2-iodobenzoic acid (0.740 g, 3.00 mmol) to produce **11a** as a white, sticky solid (0.750 g, 1.87 mmol, 62 %);
δ_H (400 MHz, $CDCl_3$) 8.86 (1H, s), 8.01 (2H, d, *J* = 8.3 Hz), 7.80 (1H, d, *J* = 8.3 Hz), 7.40 – 7.30 (4H, m), 7.08 (1H, td, *J* = 7.6, 1.9 Hz), 2.44 (3H, s);
δ_C (101 MHz, $CDCl_3$) 166.0, 145.3, 140.4, 138.6, 135.1, 132.7, 129.7, 129.0, 128.8, 128.3, 91.7, 21.8.

This data is consistent with literature precedent.[12]

3.4.2 2-Iodo-5-methyl-*N*-tosylbenzamide (12b)

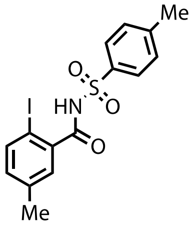

Synthesized using **GP 4** from 2-iodo-5-methylbenzoic acid (1.310 g, 5.00 mmol) to produce **11b** as a white, sticky solid (1.700 g, 3.54 mmol, 82 %);
δ_H (400 MHz, $CDCl_3$) 8.31 (1H, s), 8.06 (2H, d, *J* = 8.2 Hz), 7.70 (1H, d, *J* = 8.1 Hz), 7.37 (2H, d, *J* = 8.2 Hz), 7.22 (1H, d, *J* = 1.6 Hz), 6.94 (1H, dd, *J* = 8.1, 1.6 Hz), 2.46 (3H, s), 2.30 (3H, s);
δ_C (101 MHz, $CDCl_3$) 165.7, 145.3, 140.2, 138.9, 138.4, 135.1, 133.6, 129.7, 129.6, 128.9, 87.2, 21.8, 20.8.

This data is consistent with literature precedent.[12]

3.4.3 2-Iodo-5-bromo-*N*-tosylbenzamide (12c)

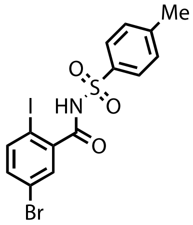

Synthesized using **GP 4** from 2-iodo-5-bromobenzoic acid (1.635 g, 5 mmol) using 5 hr reaction time to produce **11c** as a white, sticky solid (1.340 g, 2.791 mmol, 56 %);
δ_H (400 MHz, $CDCl_3$) 8.41 (1H, s), 8.04 (2H, d, *J* = 8.3 Hz), 7.67 (1H, d, *J* = 8.3 Hz), 7.45 (1H, d, *J* = 2.3 Hz), 7.35 (2H, d, *J* = 8.3 Hz), 7.21 (1H, dd, *J* = 8.4, 2.3 Hz), 2.46 (3H, s);

δC (101 MHz, $CDCl_3$) 164.5, 145.7, 141.6, 140.4, 135.6, 135.1, 131.8, 129.9, 129.0, 122.8, 89.8, 21.9.

This data is consistent with literature precedent.[13]

3.4.4 2-Iodo-3-methyl-*N*-tosylbenzamide (12d)

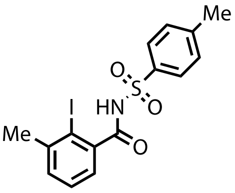

Synthesized using **GP 4** from 2-iodo-3-methylbenzoic acid (0.561 g, 2.00 mmol) to produce **11d** as a white solid (0.501 g, 1.21 mmol, 60 %);
δH (400 MHz, $CDCl_3$) 8.12 (1H, br.s), 8.05 (2H, d, *J* = 8.4 Hz), 7.38 (2H, d, *J* = 8.4 Hz), 7.33 – 7.27 (4H, m), 7.14 – 7.09 (1H, dd, *J* = 7.3, 2.1 Hz), 2.46 (2H, s), 2.44 (2H, s);
δC (101 MHz, $CDCl_3$) 166.8, 145.4, 143.3, 140.5, 135.1, 131.8, 129.7, 129.0, 128.4, 125.6, 98.2, 29.1, 21.9;
HRMS (ESI) calculated for $C_{15}H_{14}INO_3Na$ ($M+Na^+$): 437.9631; found: 437.6623;
MP 117.1 – 119.2 °C.

This data is consistent with literature precedent.[13]

3.5 General procedure 5 (GP 5): Synthesis of 2-iodo sulfonic acids 12e, f

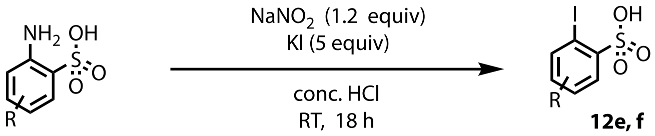

Following a previously published procedure,[14] to a solution of 2-amino sulfonic acid (5.0 – 10.0 mmol, 1.0 equiv) in concentrated HCl (1.34 M), was added a solution of $NaNO_3$ in H_2O (6.9 M, 1.3 equiv) dropwise over 10 minutes at 0 °C. After stirring for 1.5 h at 0 °C, a solution of KI in water (5.3 M, 5.0 equiv) was added dropwise over 20 minutes. The reaction was allowed to reach room temperature and stirred for 24 h. After this time, H_2O (15 mL) was added and the mixture extracted with EtOAc (3 x 20 mL). The combined organic phases were washed with saturated $Na_2S_2O_3$ solution (20 mL), brine (30 mL) and then H_2O (30 mL). The mixture was then dried over Na_2SO_4, filtered and solvent removed *in vacuo* to give the substrates **12e, 11f** without need for further purification.

3.5.1 2-Iodobenzenesulfonic acid (11e)

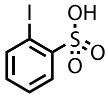

Synthesized using **GP 5** from 2-aminosulfonic acid (2.598 g, 15.00 mmol) to produce **11e** as a yellow solid (2.980 g, 10.49 mmol, 70 %);
δH (400 MHz, D_2O) 8.07 (1H, d, *J* = 7.8 Hz), 7.93 (1H, d, *J* = 7.8 Hz), 7.45 (1H, t, *J* = 7.8 Hz), 7.16 (1H, t, *J* = 7.8 Hz);
δC (101 MHz, D_2O) 144.7, 142.2, 132.6, 128.3, 128.2, 90.7.

This data is consistent with literature precedent.[15]

3.5.2 2-Iodo-5-methylbenzenesulfonic acid (11f)

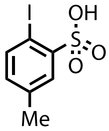

Synthesized using **GP 5** from 2-amino sulfonic acid (1.872 g, 10.00 mmol) to produce **11f** as a yellow solid (1.870 g, 6.28 mmol, 63 %);
δ_H (400 MHz, D_2O) 7.97 (1H, d, *J* = 8.0 Hz), 7.85 (1H, d, *J* = 2.2 Hz), 7.08 (1H, dd, *J* = 8.0, 2.2 Hz), 2.35 (3H, s);
δ_C (101 MHz, D_2O) 144.5, 141.5, 139.2, 133.1, 129.5, 86.6, 20.1.

This data is consistent with literature precedent.[14]

4 Synthesis of products 1-7

4.1 Synthesis of VBX reagents 1

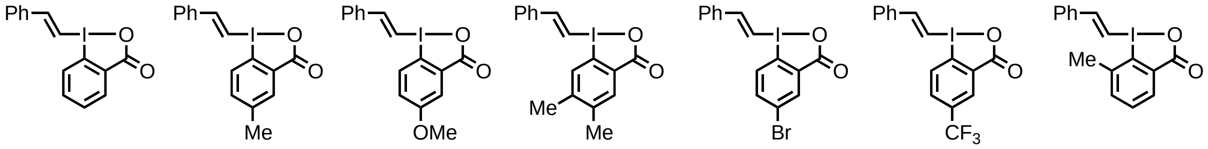

1a **1b** **1c** **1d** **1e** **1f** **1g**

Known VBX reagents were prepared according to a literature method.[16] Data for these compounds were consistent with literature precedent; **1a, c, d, e, g**[17] and **1b**[10].

4.1.1 (*E*)-1-styryl-5-(trifluoromethyl)- 1λ^3-benzo[*d*][1,2]iodaoxol-3(1*H*)-one (1f)

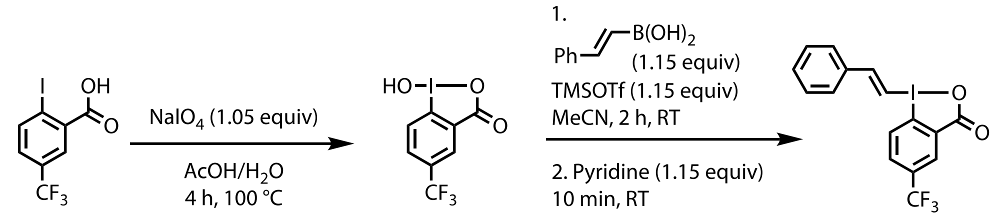

C **1e**

Following a previously published procedure,[18] $NaIO_4$ (0.441 g, 2.06 mmol, 1.05 equiv) and 2-iodo-5-(trifluoromethyl)benzoic acid (0.632 g, 2.00 mmol, 1.0 equiv) were suspended in AcOH (30% aq., 5 mL). The mixture was vigorously stirred and refluxed for 4 h. The reaction mixture was then diluted with cold water (15 mL) and allowed to cool to room temperature, protecting it from light. The crude product was collected by filtration, washed on the filter with ice water (3 x 10 mL) and acetone (3 x 10 mL), and air-dried in the dark to produce crude product **C** as a white powder (0.542 g, 1.63 mmol, 82 %), which was then directly in the next step.

Following a previously published procedure,[16] to a suspension of crude product **C** (0.287 g, 0.86 mmol, 1.0 equiv) in dry MeCN (8 mL), was added TMSOTf (0.18 mL, 0.99 mmol, 1.15 equiv) dropwise over 10 min and stirred for 30 min at room temperature. Afterwards (*E*)-styrylboronic acid (0.147 g, 0.99 mmol, 1.15 equiv) was added over 5 min and the reaction mixture was stirred for 1.5 h at room temperature. Pyridine (0.080 mL, 0.99 mmol, 1.15 equiv) was added and after further 10 min stirring, the solvent was removed under reduced pressure. The residue was dissolved in CH_2Cl_2 (20 mL) and washed with 1 M HCl (20 mL). The aqueous phase was extracted three times with CH_2Cl_2 (3 x 20 mL) and the combined organic phases were washed with a saturated solution of $NaHCO_3$ (20 mL), dried over Na_2SO_4, filtered and concentrated under reduce pressure. The residue was dissolved in a minimum amount of CH_2Cl_2 and precipitated in Et_2O (30 mL) was induced by vigorous stirring for 30 min and storing in the freezer overnight. The precipitate was filtered and washed with Et_2O (20 mL) to afford **1e** as a white solid (0.166 g, 0.397 mmol, 46 %, 36% over two steps).

δ_H (400 MHz, MeOD-*d₄*) δ 8.52 (1H, d, *J* = 1.7 Hz), 8.03 - 7.94 (3H, m), 7.73 - 7.69 (3H, m), 7.52 - 7.49 (3H, m);
δ_F (377 MHz, MeOD-d_4) -64.29;

δC (101 MHz, MeOD-*d*4) 168.5, 156.3, 136.6, 136.0, 134.3 (q, *J* = 33.0 Hz), 134.0, 132.3, 131.3 (q, *J* = 3.8 Hz), 130.5, 130.2, 129.5 (q, *J* = 2.9 Hz), 129.1, 124.9 (q, *J* = 270.3 Hz), 123.5, 119.7, 100.2;
HRMS (ESI) calculated for $C_{16}H_{10}F_3IO_2Na^+$ ($M+Na^+$): 440.9570; found: 440.9585;
MP 159.0 – 161.0 °C.

4.2 General procedure 6 (GP 6): Synthesis of VBO reagents 2

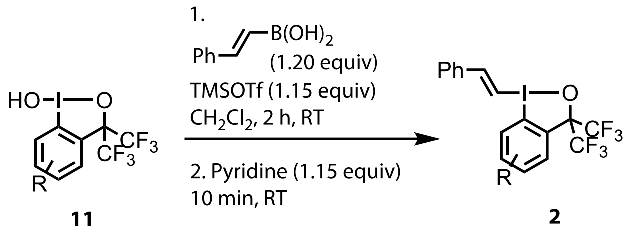

Following a modified version of a previously published procedure,[16] hydroxybenziodoxole **11** (1.08 – 4.05 mmol, 1.0 equiv) was suspended in dry CH_2Cl_2 (0.12 M) followed by the dropwise addition of TMSOTf (1.15 equiv) over 10 min, and the solution was stirred for 30 min at room temperature. Then, (*E*)-styrylboronic acid (1.20 equiv) was slowly added in portions over 5 min and further stirred for 1.5 h at room temperature. Lastly, pyridine (1.15 equiv) was added followed by stirring for 10 min at room temperature. Afterwards, the solvent was evaporated and the residue was dissolved in CH_2Cl_2 (50 mL) and washed with 1M HCl (25 mL). The aqueous phase was extracted with CH_2Cl_2 (2 x 25 mL) and then the combined organic layers were washed with saturated NaHCO3 solution (25 mL), dried over anhydrous MgSO4, filtered and evaporated *in vacuo*. After column chromatography was performed to produce **2b** – **2f.** NOTE: these compounds seem to decompose quite quickly whilst in solution. So, during isolation, it is important to be as quick as possible and to store in the freezer.

4.2.1 (*E*)-1-styryl-3,3-bis(trifluoromethyl)-1,3-dihydro-1λ3-benzo[*d*][1,2]iodaoxole (2a)

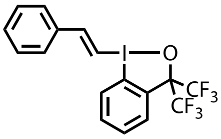

Synthesised from **11a** (0.957 g, 2.48 mmol, 1 equiv) according to a literature protocol[19] to produce **2a** as a white solid (1.030 g, 2.18 mmol, 88%).

4.2.2 (*E*)-5-methyl-1-styryl-3,3-bis(trifluoromethyl)-1,3-dihydro-1λ3-benzo[*d*][1,2]-iodaoxole (2b)

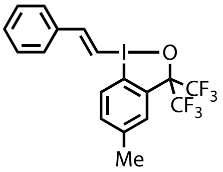

Synthesised following **GP 6** from **11b** (1.214 g, 3.03 mmol). Afterwards, column chromatography (pentane:EtOAc 100:0 →1:1) was performed to obtain **2b** as a white solid (0.895 g, 1.84 mmol, 61 %).
Rf 0.25 (pentane:EtOAc 1:1);
δH (400 MHz, CDCl3) 7.66 (1H, s), 7.61 (2H, d, *J* = 16.0 Hz), 7.52 – 7.42 (1H, m), 7.37 (1H, d, *J* = 8.2 Hz), 7.32 (1H, dd, *J* = 8.2, 2.0 Hz), 7.21 (1H, d, *J* = 16.0 Hz), 2.46 (3H, s);
δF (376 MHz, CDCl3) -76.03;
δC (101 MHz, CDCl3) 151.8, 141.3, 135.6, 133.1, 131.3, 131.1 (pent, *J* = 2.7 Hz), 130.7, 129.3, 127.4, 127.1, 124.2 (q, *J* = 296.4 Hz), 107.1, 104.9, 81.3 (pent, *J* = 29.3 Hz), 21.2;

HRMS calculated for $C_{18}H_{14}F_6IO^+$ (M+H$^+$): 486.9988; found: 486.9964;
MP 174.0 – 177.0 °C.

4.2.3 (*E*)-5-methoxy-1-styryl-3,3-bis(trifluoromethyl)-1,3-dihydro-1λ^3-benzo[*d*][1,2]iodaoxole (2c)

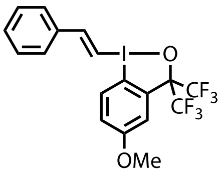

Synthesised following **GP 6** from **11c** (0.473 g, 1.08 mmol) using 18 h reaction time. Afterwards, column chromatography (pentane:EtOAc 100:0 → 1:1) was performed to obtain **2c** as a white solid (0.220 g, 0.44 mmol, 44 %).
R$_f$ 0.25 (1:1 pentane:EtOAc);
δ$_H$ (400 MHz, CDCl$_3$) 7.62 (1H, d, *J* = 16.0 Hz), 7.54 – 7.42 (5H, m), 7.40 – 7.32 (2H, m), 7.21 (1H, d, *J* = 16.0 Hz), 7.05 (1H, dd, *J* = 9.4, 2.4 Hz), 3.86 (3H, s);
δ$_F$ (376 MHz, CDCl$_3$) -76.06;
δ$_C$ (101 MHz, CDCl$_3$) 161.9, 151.9, 135.5, 132.7, 130.8, 129.3, 127.9, 127.4, 124.2 (q, *J* = 294.4 Hz), 118.3, 116.1 (sept, *J* = 2.5 Hz), 104.4, 99.5, 81.1 (pent, *J* = 23.4 Hz), 56.0;
HRMS (ESI) calculated for $C_{18}H_{14}F_6IO_2$ (M+H$^+$): 502.9937; found: 502.9933;
MP 164.0 – 166.5 °C (decomposition).

4.2.4 (*E*)-5,6-dimethyl-1-styryl-3,3-bis(trifluoromethyl)-1,3-dihydro-1λ^3-benzo[*d*][1,2]iodaoxole (2d)

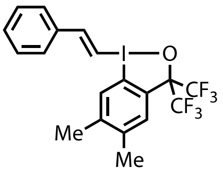

Synthesised following **GP 6** from **11d** (0.715 g, 1.73 mmol). Afterwards, column chromatography (pentane:EtOAc 100:0 → 1:1) was performed to obtain **2d** as a white solid (0.358 g, 0.716 mmol, 42 %).
R$_f$ 0.25 (1:1 pentane:EtOAc);
δ$_H$ (400 MHz, CDCl$_3$) 7.66 – 7.60 (2H, m), 7.55 – 7.45 (5H, m), 7.25 (1H, d, *J* = 16.3 Hz), 7.20 (1H, s), 2.37 (3H, s), 2.31 (3H, s);
δ$_F$ (376 MHz, CDCl$_3$) -76.11;
δ$_C$ (101 MHz, CDCl$_3$) 151.6, 141.7, 139.9, 135.7, 131.3 (pent, *J* = 2.4 Hz), 130.7, 129.3, 128.5, 127.7, 127.4, 124.4 (q, *J* = 292.2 Hz), 107.8, 104.9, 81.2 (pent, *J* = 29.6 Hz), 20.3, 19.9;
HRMS (ESI) calculated for $C_{19}H_{16}F_6IO$ (M+H$^+$): 501.0145; found: 501.0161;
MP 174.0 – 177.0 °C.

4.2.5 (*E*)-5-bromo-1-styryl-3,3-bis(trifluoromethyl)-1,3-dihydro-1λ^3-benzo[*d*][1,2]iodaoxole (2e)

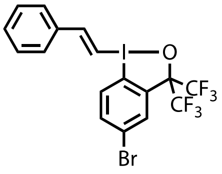

Synthesised following **GP 6** from **11e** (1.891 g, 4.05 mmol) and was suspended in dry CH_3CN (32 mL) instead of CH_2Cl_2. Afterwards, column chromatography (pentane:EtOAc 100:0 → 2:1) was performed and additional flushing of the column with EtOAc afforded **2e** as white solid white solid (1.162 g, 2.11 mmol, 52 %);
R$_f$ 0.3 (pentane:EtOAc 2:1);
δ$_H$ (400 MHz, CDCl$_3$) 7.96 (1H, s), 7.68 – 7.60 (2H, m), 7.55 – 7.44 (5H, m), 7.38 (1H, d, *J* = 8.7 Hz), 7.20 (1H, d, *J* = 16.2 Hz);
δ$_F$ (376 MHz, CDCl$_3$) –76.01;

δ_C (101 MHz, CDCl$_3$) 152.8, 135.3, 135.2, 133.5 (pent, J = 2.0 Hz), 133.4 131.0, 129.4, 128.7, 127.5, 125.7, 124.0 (q, J = 291.7 Hz), 109.9, 104.2, 81.2 (pent, J = 29.4 Hz);
HRMS (ESI): calculated for $C_{17}H_{11}{}^{79}BrF_6IO$ (M+H$^+$): 550.8937; found: 550.8971;
MP 173.0 – 175.0 °C.

4.2.6 (*E*)-5,7-dimethyl-1-styryl-3,3-bis(trifluoromethyl)-1,3-dihydro-1λ^3-benzo[*d*][1,2]iodaoxole (2f)

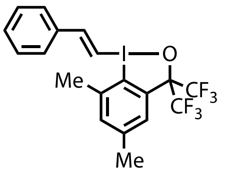

Synthesised following **GP 6** from **11f** (0.828 g, 2.00 mmol) using 18 h reaction time. Afterwards, column chromatography (pentane:EtOAc 100:0 →1:1) was performed to obtain **2f** as a white solid (0.356 g, 0.712 mmol, 27 %). NOTE: this compound seems to decompose quite quickly whilst in solution. So, during isolation, it is important to be as quick as possible and to store in the freezer;
R$_f$ 0.3 (2:8 pentane:EtOAc);
δ_H (500 MHz, CDCl$_3$) 7.54 (1H, d, J = 15.5 Hz), 7.49 – 7.43 (2H, m), 7.42 – 7.34 (5H, m), 7.14 (1H, s), 2.46 (3H, s), 2.39 (3H, s);
δ_F (376 MHz, CDCl$_3$) -74.94;
δ_C (125 MHz, CDCl$_3$) 147.1, 140.8, 139.5, 136.1, 135.5, 135.1, 130.4, 129.2, 128.8, 127.3, 124.4 (q, J = 294.5 Hz), 112.8, 109.4, 81.6 (t, J = 29.3 Hz), 25.9, 20.8;
HRMS (ESI) calculated for $C_{19}H_{16}F_6IO$ (M+H$^+$): 501.0145; found: 501.0167;
MP 157.8 – 159.2 °C (decomposition).

4.3 General procedure 7 (GP 7): One-pot synthesis of VBZ derivatives 3a–c

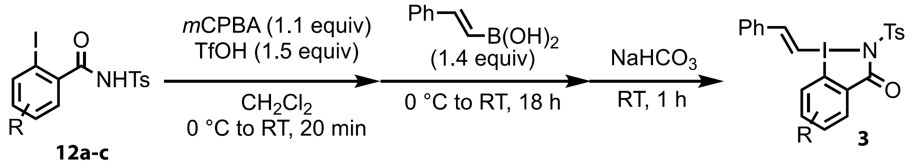

Following a modified version of a previously published procedure,[20] 2-iodobenzamide **12a-c** (0.54 – 2.62 mmol, 1.0 equiv) was added to a round bottom flask followed by CH_2Cl_2 (0.5 M). *m*CPBA (1.1 equiv) was added, and the mixture was cooled to 0 °C followed by the addition of TfOH (1.5 equiv). The mixture was stirred at room temperature for 15 minutes and then cooled to 0 °C for 5 minutes. The corresponding boronic acid (1.4 equiv) was added. The mixture was stirred at room temperature for 18 h. Saturated $NaHCO_3$ (30 mL) was added and the mixture was stirred vigorously at room temperature for 1 h. The reaction mixture was transferred to a separation funnel, diluted with CH_2Cl_2 (20 mL) and H_2O (20 mL). Note: dilution helped to avoid emulsions in the separation. The layers were separated and the aqueous phase was extracted three times with CH_2Cl_2 (20 mL). The combined organic phases were washed with H_2O (20 mL) and brine (20 mL) and then dried over Na_2SO_4. The drying agent was filtered off and the solvent was removed *in vacuo*. Often a sticky, brown oil was observed at this point. Et_2O (50 mL) was added and the crude mixture was stirred for 30 min, and then stored in the freezer for 18 h. After filtration, the solid was then washed with EtOAc (20 mL), pentane (20 mL) and dried *in vacuo*. This usually resulted in clean product **3** as an off-white solid.

4.3.1 (*E*)-1-styryl-2-tosyl-1,2-dihydro-3H-1λ^3-benzo[*d*][1,2]iodazol-3-one (3a)

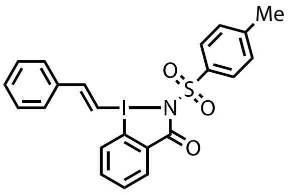

Synthesised following **GP 7** from **12a** (1.050 g, 2.62 mmol) to produce **3a** as a white solid (0.941 g, 1.87 mmol, 72 %);
δ_H (400 MHz, DMSO-*d_6*) 8.15 (1H, d, *J* = 8.1 Hz), 7.99 (1H, d, *J* = 15.3 Hz), 7.87 – 7.79 (3H, m), 7.74 – 7.61 (5H, m), 7.52 – 7.45 (3H, m), 7.29 (2H, d, *J* = 8.1 Hz), 2.36 (3H, s);
δ_C (101 MHz, DMSO-*d_6*) 164.3, 152.3, 141.2, 140.8, 135.4, 134.7, 134.3, 131.2, 130.6, 130.5, 129.1, 128.9, 128.7, 127.8, 127.0, 114.1, 104.1, 20.9;
HRMS (ESI) calculated for $C_{22}H_{18}INNaO_3S$ (M+Na$^+$): 525.9944; found: 525.9925;
MP 177.2 - 180 °C (decomposition).

4.3.2 (*E*)-5-methyl-1-styryl-2-tosyl-1,2-dihydro-3H-1λ^3-benzo[*d*][1,2]iodazol-3-one (3b)

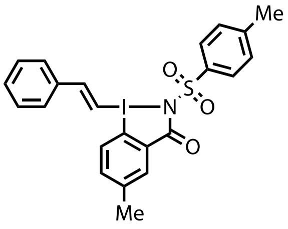

Synthesised following **GP 7** from **12b** (0.950 g, 2.33 mmol) to produce **3b** as a white solid (0.921 g, 1.78 mmol, 78 %);
δ_H (400 MHz, DMSO-*d_6*) 8.02 – 7.94 (2H, m), 7.86 – 7.77 (3H, m), 7.72 – 7.67 (2H, m), 7.57 – 7.43 (5H, m), 7.30 (2H, d, *J* = 8.0 Hz), 2.36 (s, 3H), 2.35 (s, 3H);
δ_C (101 MHz, DMSO-*d_6*) 164.5, 152.1, 141.1, 140.9, 140.5, 135.4, 134.9, 134.5, 131.7, 130.6, 129.1, 128.6, 128.6, 127.8, 127.0, 110.3, 103.7, 20.9, 20.2;
HRMS (ESI) calculated for $C_{23}H_{20}INNaO_3S$ (M+Na$^+$): 540.0101; found: 540.0092;
MP 189.7 – 192.1 °C.

4.3.3 (*E*)-5-bromo-1-styryl-2-tosyl-1,2-dihydro-3H-1λ^3-benzo[*d*][1,2]iodazol-3-one (3c)

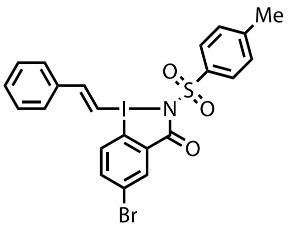

Synthesised following **GP 7** from **12c** (1.200 g, 2.57 mmol) to produce **3c** as a white solid (1.051 g, 1.80 mmol, 73 %).
δ_H (400 MHz, DMSO-*d_6*) 8.20 (1H, d, *J* = 2.5 Hz), 8.01 (1H, d, *J* = 15.4 Hz), 7.91 – 7.77 (4H, m), 7.69 (2H, d, *J* = 6.4 Hz), 7.61 (1H, d, *J* = 8.6 Hz), 7.54 – 7.44 (3H, m), 7.30 (2H, d, *J* = 8.6 Hz), 2.36 (3H, s);
δ_C (101 MHz, DMSO-*d_6*) 163.0, 152.6, 141.4, 140.5, 136.7, 136.6, 135.3, 133.2, 131.1, 130.7, 129.1, 128.7, 127.8, 127.1, 124.3, 113.0, 103.9, 20.9;
HRMS (ESI) calculated for $C_{22}H_{17}{}^{79}BrINO_3SNa$ (M+Na$^+$): 603.9049; found: 603.9081;
MP 183.7 – 186.3 °C.

4.4 (*E*)-4-methyl-*N*-((*Z*)-7-methyl-1-((*E*)-styryl)-1λ^3 -benzo[*d*][1,2]iodaoxol-3(1*H*)-ylidene)benzenesulfonamide (4)

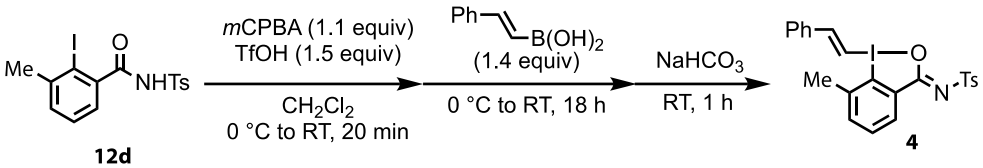

Following a modified version of a previously published procedure,[20] 2-iodobenzamide **12d** (0.233 g, 0.54 mmol, 1.0 equiv) was added to a round bottom flask followed by CH_2Cl_2 (3.8 mL). *m*CPBA (0.107 g, 0.59 mmol, 1.1 equiv) was added, and the mixture was cooled to 0 °C followed by the addition of TfOH (0.050 mL, 0.56 mmol, 1.05 equiv). The mixture was stirred at room temperature for 15 minutes and then cooled to 0 °C for 5 minutes. Then (*E*)-styrylboronic acid (0.134 g, 0.75 mmol, 1.4 equiv) was added. The mixture was stirred at room temperature for 18 h. Saturated $NaHCO_3$ (15 mL) was added and the mixture was stirred vigorously at room temperature for 1 h. The reaction mixture was transferred to a separation funnel, diluted with CH_2Cl_2 (10 mL) and H_2O (10 mL). Note: dilution helped to avoid emulsions in the separation. The layers were separated and the aqueous phase was extracted three times with CH_2Cl_2 (10 mL). The combined organic phases were washed with H_2O (10 mL) and brine (10 mL) and then dried over Na_2SO_4. The drying agent was filtered off and the solvent was removed *in vacuo*. Often a sticky, brown oil was observed at this point. Et_2O (30 mL) was added and the crude mixture was stirred for 30 min, and then stored in the freezer for 18 h. After filtration column chromatography (2% MeOH:EtOAc) was performed to produce **4** as a white solid (100 mg, 0.19 mmol, 36 %). NOTE: this compound seems to decompose quite quickly whilst in solution. So, during isolation, it is important to be as quick as possible and to store in the freezer.

R_f 0.4 (EtOAc:MeOH 98:2);
δ_H (400 MHz, $CDCl_3$) 8.13 (1H, dd, *J* = 7.5, 2.0 Hz), 8.01 (2H, d, *J* = 8.3 Hz), 7.64 (1H, d, *J* = 15.4 Hz), 7.54 (1H, d, *J* = 15.4 Hz), 7.44 – 7.31 (8H, m), 7.23 (1H, d, *J* = 8.0 Hz), 2.50 (3H, s), 2.36 (3H, s);
δ_C (125 MHz, DMSO-d_6) 168.1, 148.5, 142.1, 141.3, 141.2, 141.1, 135.7, 135.3, 131.2, 130.9, 129.5, 129.1, 129.0, 128.0, 127.6, 118.9, 105.9, 26.3, 21.4;
HRMS (ESI) calculated for $C_{23}H_{20}INO_3SNa$ ($M+Na^+$): 540.0101; found: 540.0100;
MP 97.4 – 101.3 °C (decomposition).

4.5 General procedure 8 (GP 8): one-pot synthesis of VBS derivatives 5a,b

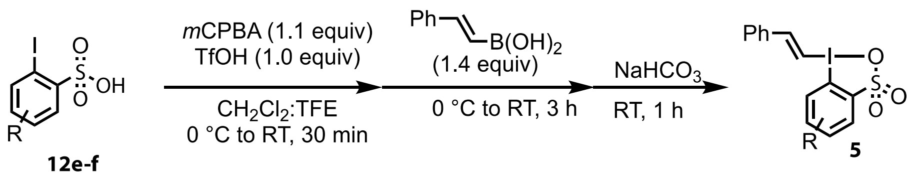

Following a modified version of a previously published procedure,[20] 2-iodobenzenesulfonic acid **12e-f** (1.03 – 2.06 mmol, 1.0 equiv) was added to a round bottom flask followed by CH_2Cl_2:TFE (3:1, 0.1 M). *m*CPBA (1.1 equiv) was added, and the mixture was cooled to 0 °C followed by the addition of TfOH (1.0 equiv). The mixture was stirred at room temperature for 30 minutes and then cooled to 0 °C for 5 minutes. The corresponding boronic acid (1.4 equiv)

was added. The mixture was stirred at room temperature for 3 h. Saturated $NaHCO_3$ was added and the mixture was stirred vigorously at room temperature for 1 h. The reaction mixture was transferred to a separation funnel, diluted with CH_2Cl_2 (20 mL) and H_2O (20 mL). Note: dilution helped to avoid emulsions in the separation. The layers were separated and the aqueous phase was extracted with CH_2Cl_2 (3 x 20 mL). The combined organic phases were washed with H_2O (20 mL) and brine (20 mL) and then dried over Na_2SO_4. The drying agent was filtered off and the solvent was removed in vacuo. Et_2O (50 mL) was added and the crude mixture was stirred for 30 min, and then stored in the freezer for 18 h. This usually resulted in clean product **5** after filtering the solid.

4.5.1 (*E*)-1-styryl-1H-1λ^3-benzo[*d*][1,2,3]iodaoxathiole 3,3-dioxide (5a)

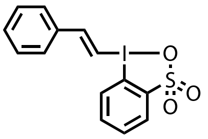

Synthesised following **GP 8** from **12e** (0.852 g, 3.00 mmol, 1 equiv) to produce **5a** as a pale-yellow solid (0.792 g, 2.05 mmol, 68 %).
δ_H (400 MHz, DMSO-*d*$_6$) 8.16 (1H, d, *J* = 15.6 Hz), 8.00 (1H, d, *J* = 15.6 Hz), 7.92 (1H, dd, *J* = 7.5, 1.7 Hz), 7.75 – 7.69 (3H, m), 7.66 (1H, t, *J* = 7.5 Hz), 7.60 (1H, t, *J* = 6.8 Hz), 7.56 – 7.48 (3H, m).
δ_C (101 MHz, DMSO-*d*$_6$) 154.0, 145.3, 134.9, 132.7, 131.2, 131.1, 129.9, 129.2, 128.7, 128.0, 109.8, 99.1;
HRMS (ESI) calculated for $C_{14}H_{11}INaO_3S$ ($M+Na^+$): 408.9366; found: 408.9362;
MP 191.2 – 193.1 °C (decomposition).

4.5.2 (*E*)-5-methyl-1-styryl-1H-1λ^3-benzo[*d*][1,2,3]iodaoxathiole 3,3-dioxide (5b)

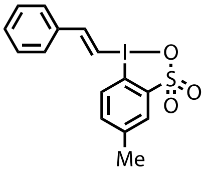

Synthesised following **GP 8** from **12f** (0.596 g, 2.06 mmol) to produce **5b** as a pale-yellow solid (0.310 g, 0.78 mmol, 39 %);
δ_H (400 MHz, DMSO-*d*$_6$) 8.13 (1H, d, *J* = 14.1 Hz), 7.99 (1H, d, *J* = 14.1 Hz), 7.99 – 7.67 (3H, m), 7.62 – 7.47 (4H, m), 7.41 (1H, d, *J* = 7.6 Hz), 2.38 (3H, s);
δ_C (101 MHz, DMSO-*d*$_6$) 153.7, 145.1, 145.1, 141.4, 134.9, 133.2, 131.1, 129.7, 129.1, 128.0, 106.0, 99.0, 20.2;
HRMS (ESI) calculated for $C_{15}H_{13}INaO_3S$ ($M+Na^+$): 422.9522; found: 422.9517;
MP 196.8 – 197.6 °C (decomposition).

4.6 General procedure 9 (GP 9): one-pot synthesis of iodonium salts 6

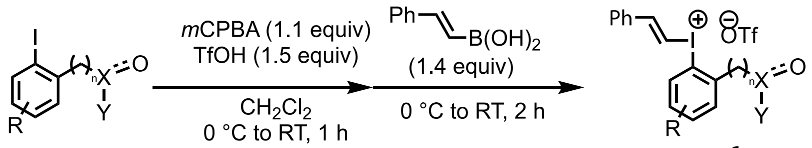

6

Following a modified version of a previously published procedure,[20] iodoarene (1.00 – 2.00 mmol, 1.0 equiv) was added to a round bottom flask followed by CH_2Cl_2 (7.7 – 14.4 mL, 0.13 M). *m*CPBA (1.1 equiv) was added, and the mixture was cooled to 0 °C followed by the addition of TfOH (1.5 equiv). The mixture was stirred at room temperature for 1 h and then cooled to 0 °C for 5 minutes. The corresponding boronic acid (1.4 equiv) was added in one portion and rinsed down with CH_2Cl_2 (5 – 10 mL). The mixture was stirred at room temperature for 2 h. The solvent was removed *in vacuo* and Et_2O (20 mL) was added. If a white precipitate was not

formed immediately, the mixture was allowed stir vigorously at room temperature for 30 minutes, then left in the freezer overnight. The solid was filtered off (glass filter funnel, porosity 3) and washed with Et_2O (3 x 25 mL) to obtain iodonium salts **6**.

4.6.1 (*E*)-(2-(methoxycarbonyl)phenyl)(styryl)iodonium trifluoromethanesulfonate (6a)

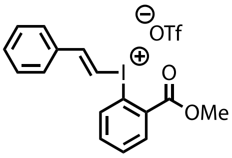

Synthesised following **GP 9** from 2-iodo methyl benzoate (0.524 g, 2.00 mmol) to produce **6a** as an off-white solid (0.462 g, 0.91 mmol, 45 %);
δH (400 MHz, MeOD-*d4*) 8.39 (1H, dd, *J* = 7.5, 1.8 Hz), 8.15 (1H, d, *J* = 14.9 Hz), 7.93 (1H, dd, *J* = 8.2, 1.3 Hz), 7.87 (1H, td, *J* = 7.7, 1.8 Hz), 7.83 – 7.71 (4H, m), 7.55 – 7.49 (3H, m), 4.13 (3H, s);
δF (376 MHz, MeOD-*d4*) -79.95;
δC (101 MHz, MeOD-*d4*) 169.8, 158.3, 138.1, 135.9, 134.0, 132.9, 132.6, 131.4, 130.3, 129.4, 128.1, 121.6 (q, *J* = 317.6 Hz), 114.8, 95.7, 55.1;
HRMS (ESI) calculated for $C_{16}H_{14}IO_2$ (M-[OTf⁻]⁺): 365.0033; found: 365.0039
MP 139.7 – 143.6 °C (decomposition).

4.6.2 (*E*)-(4-methyl-2-sulfophenyl)(styryl)iodonium trifluoromethanesulfonate (6b)

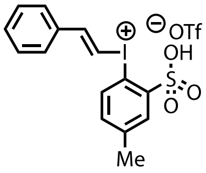

Synthesised following **GP 9** from **3b** (0.380 g, 1.28 mmol) to produce **6b** as an off-white solid (0.412 g, 0.823 mmol, 82 %);
δH (400 MHz, DMSO-*d6*) 8.13 (1H, d, *J* = 14.8 Hz), 7.99 (1H, d, *J* = 14.8 Hz), 7.77 – 7.66 (3H, m), 7.61 – 7.48 (4H, m), 7.41 (1H, d, *J* = 8.3 Hz), 2.38 (3H, s);
δF (376 MHz, MeOD-*d4*) -79.98;
δC (101 MHz, DMSO-*d6*) 153.7, 145.2, 141.4, 134.9, 133.2, 131.1, 129.7, 129.2, 128.1 (q, *J* = 289.9 Hz), 128.0, 123.3, 106.0, 99.0, 20.2;
HRMS (ESI) calculated for $C_{15}H_{13}INaO_3S$ (M-[OTf⁻]+Na⁺): 422.9522; found: 422.9509;
MP 187.6 – 188.9 °C (decomposition).

4.6.3 (*E*)-(4-bromo-2-carboxyphenyl)(styryl)iodonium trifluoromethanesulfonate (6c)

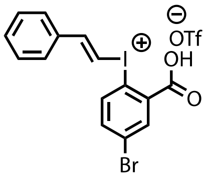

Synthesised following **GP 9** from 2-iodo-5-bromobenzoic acid (0.654 g, 2.00 mmol) to produce **6c** as an off-white solid (0.456 g, 0.79 mmol, 39 %);
δH (400 MHz, MeOD-*d4*) 8.48 (1H, d, *J* = 2.4 Hz), 8.12 (1H, d, *J* = 14.9 Hz), 8.00 (1H, dd, *J* = 8.7, 2.4 Hz), 7.80 – 7.70 (4H, m), 7.56 – 7.48 (3H, m, 3H);
δF (376 MHz, MeOD-*d4*) -80.03;
δC (101 MHz, MeOD-*d4*) 169.7, 158.2, 140.2, 136.6, 136.0, 132.9, 132.5, 130.9, 130.3, 129.4, 126.8, 121.6 (q, *J* = 317.6 Hz), 113.6, 96.1, 15.4;
HRMS (ESI) calculated for $C_{15}H_{10}{}^{79}BrINaO_2$ (M-[OTf⁻]-H⁺+Na⁺): 450.8801; found: 450.8802;
MP 160.1 – 160.7 °C (decomposition).

4.6.4 (*E*)-3-(2-(styryl)iodaneyl)phenyl)propanoic acid trifluoromethanesulfonate (6d)

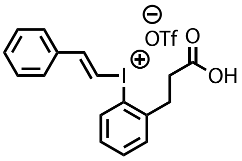

Synthesised following **GP 9** from 2-iodo-5-bromobenzoic acid (0.276 g, 1.00 mmol) to produce **6d** as an off-white solid (0.213 g, 0.403 mmol, 40 %);

δ_H (400 MHz, MeOD-*d_4*) 8.20 (1H, d, *J* = 8.7 Hz), 7.95 (1H, d, *J* = 14.5 Hz), 7.80 (1H, d, *J* = 14.5 Hz), 7.70 – 7.62 (2H, m), 7.54 (2H, dd, *J* = 6.7, 3.0 Hz), 7.46 – 7.40 (3H, m), 7.37 (1H, ddd, *J* = 8.7, 6.7, 2.4 Hz), 3.24 (2H, t, *J* = 6.7 Hz), 2.82 (2H, t, J = 6.7 Hz);

δ_F (376 MHz, MeOD-*d_4*) -80.03;

δ_C (101 MHz, MeOD-*d_4*) 176.4, 151.9, 145.1, 138.2, 135.9, 134.4, 132.3, 132.2, 131.3, 130.2, 128.9, 121.6 (q, *J* = 317.6 Hz), 119.9, 99.7, 35.4, 34.6;

HRMS (ESI) calculated for $C_{17}H_{16}IO2$ (M-[OTf$^-$]$^+$): 379.0189; found: 379.0201;

MP 147.3 – 149.7 °C (decomposition).

4.6.5 (*E*)-(2-(carboxymethyl)phenyl)(styryl)iodonium trifluoromethanesulfonate (6e)

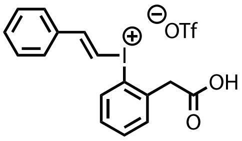

Synthesised following **GP 9** from 2-(2-iodophenyl)acetic acid (0.262 g, 1.00 mmol) to produce **6e** as an off-white solid (0.376 g, 0.73 mmol, 73 %);

δ_H (400 MHz, DMSO-*d_6*) 8.30 (1H, d, *J* = 8.0 Hz), 7.89 (1H, d, *J* = 14.5 Hz), 7.83 (1H, d, *J* = 14.5 Hz), 7.68 – 7.63 (2H, m), 7.53 – 7.50 (2H, m), 7.47 – 7.42 (4H, m), 4.03 (2H, s);

δ_F (376 MHz, DMSO-*d_6*) -72.99;

δ_C (101 MHz, DMSO-*d_6*) 172.1, 148.5, 138.1, 137.3, 134.6, 132.6, 132.5, 130.7, 130.4, 129.1, 128.8 (q, *J* = 289.9 Hz), 127.6, 119.4, 102.1, 43.1, 40.12;

HRMS (ESI) calculated for $C_{16}H_{14}IO_2$ (M-[OTf$^-$]$^+$): 365.0033; found: 365.0016;

MP 183.6 – 185.1 °C (decomposition).

4.6.6 (*E*)-1-styryl-1,4-dihydro-3H-1λ^3-benzo[*d*][1,2]iodaoxin-3-one (7)

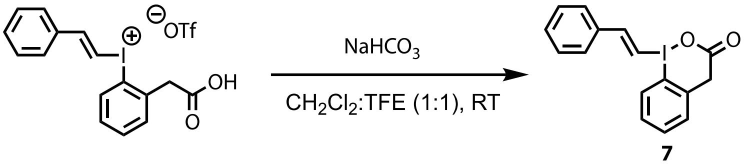

Iodonium salt **5e** (0.200 g, 0.42 mmol, 1.0 equiv) was added to a round bottom flask followed by CH_2Cl_2:TFE (1:1, 5 mL). Saturated $NaHCO_3$ (20 mL) was added and the mixture was stirred vigorously at room temperature for 16 h. The reaction mixture was transferred to a separation funnel, diluted with CH_2Cl_2 (20 mL) and H_2O (20 mL). Note: dilution helped to avoid emulsions in the separation. The layers were separated and the aqueous phase was extracted with CH_2Cl_2 (3 x 20 mL). The combined organic phases were washed with H_2O (20 mL) and brine (20 mL) and then dried over Na_2SO_4. The drying agent was filtered off and the solvent was removed in vacuo. Et_2O (50 mL) was added and the crude mixture was stirred for 30 min, and then stored in the freezer for 18 h. This resulted in **7** as an off-white solid (0.087 g, 0.24 mmol, 57 %).

δ_H (400 MHz, MeOD-*d*$_4$) 8.03 – 7.92 (2H, m), 7.75 (1H, d, *J* = 15.1 Hz), 7.63 – 7.47 (4H, m), 7.47 – 7.40 (3H, m), 7.34 (1H, ddd, *J* = 8.8, 6.5, 2.5 Hz), 3.77 (2H, s);
δ_C (101 MHz, MeOD-*d*$_4$ 177.8, 152.5, 141.6, 136.5, 134.6, 132.8, 132.7, 131.9, 130.7, 130.2, 128.8, 119.6, 102.4, 47.2;
HRMS (ESI) calculated for $C_{16}H_{14}IO_2$ ($M+H^+$): 365.0033; found: 365.0015;
MP 381.7 – 383.7 °C (decomposition).

5 Failed reactions

5.1.1 Use of tosic acid in the synthesis of VBZ

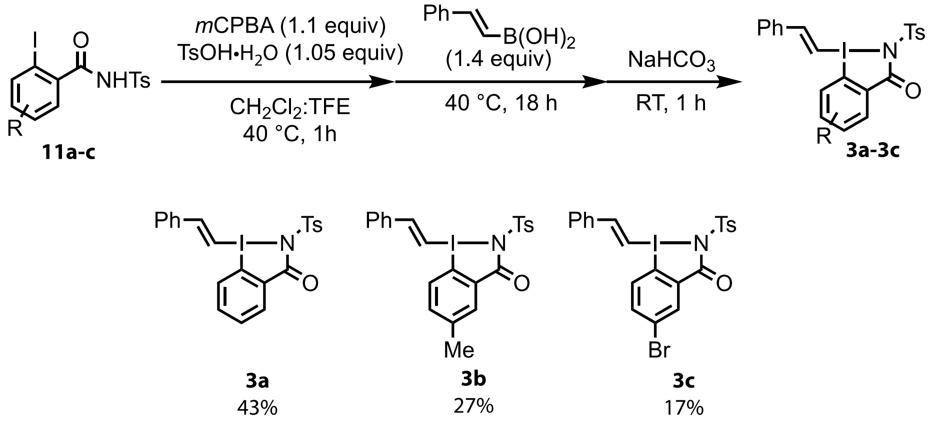

Following a previously published procedure,[21] 2-iodobenzamide **12a-c** (1.0 equiv) was added to a round bottom flask followed by CH_2Cl_2:TFE (1:1) (0.5 M). *m*CPBA (1.1 equiv) was added and tosic acid monohydrate (1.5 equiv) was added. The mixture was stirred at 40 °C for 1 h. (*E*)-Styrylboronic acid (1.4 equiv) was added. The mixture was stirred at 40 °C for 18 h. After cooling to room temperature, saturated $NaHCO_3$ (30 mL) was added and the mixture was stirred vigorously at room temperature for 1 h. The reaction mixture was transferred to a separation funnel, diluted with CH_2Cl_2 (20 mL) and H_2O (20 mL). Note: dilution helped to avoid emulsions in the separation. The layers were separated and the aqueous phase was extracted three times with CH_2Cl_2 (20 mL). The combined organic phases were washed with H_2O (20 mL) and brine (20 mL) and then dried over Na_2SO_4. The drying agent was filtered off and the solvent was removed *in vacuo*. Often a sticky, brown oil was observed at this point. Et_2O (50 mL) was added and the crude mixture was stirred for 30 min, and then stored in the freezer for 18 h. This mixture was then purified via column chromatography (MeOH:CH_2Cl_2) to obtain **4a-c** as off-white powders.

5.1.2 Synthesis of (*E*)-1-styryl-1,4-dihydro-3H-1λ^3-benzo[*d*][1,2]iodaoxin-3-one (7)

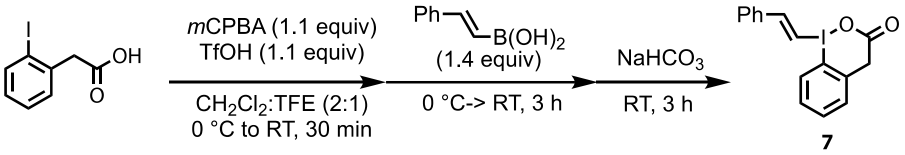

Following a previously published procedure,[20] 2-(2-iodophenyl)acetic acid (0.655 g, 2.50 mmol, 1.0 equiv) was added to a round bottom flask followed by CH_2Cl_2:TFE (2:1, 15 mL). *m*CPBA (0.593, 2.75 mmol, 1.1 equiv) was added, and the mixture was cooled to 0 °C followed by the addition of TfOH (0.243 mL, 2.75 mmol, 1.1 equiv). The mixture was stirred at room temperature for 30 minutes and then cooled to 0 °C and stirred for 5 minutes. (*E*)-styrylboronic acid (0.624 g, 3.5 mmol, 1.4 equiv) was added at 0 °C. The mixture was stirred at room temperature for 3 h. Saturated $NaHCO_3$ was added and the mixture was stirred vigorously at room temperature for 1 h. The reaction mixture was transferred to a separation funnel, diluted with CH_2Cl_2 (20 mL) and H_2O (20 mL). Note: dilution helped to avoid emulsions in the separation. The layers were separated and the aqueous phase was extracted with CH_2Cl_2 (3 x 20 mL). The combined organic phases were washed with H_2O (20 mL) and brine (20 mL) and

then dried over Na_2SO_4. The drying agent was filtered off and the solvent was removed in vacuo. Et_2O (50 mL) was added and the crude mixture was stirred for 30 min, and then stored in the freezer for 18 h. This resulted in impure **7,** that was unable to be purified.

5.1.3 Failed synthesis of (*E*)-1-styryl-1,5-dihydro-3H-1λ³-benzo[*d*][1,2]iodaoxin-4-one

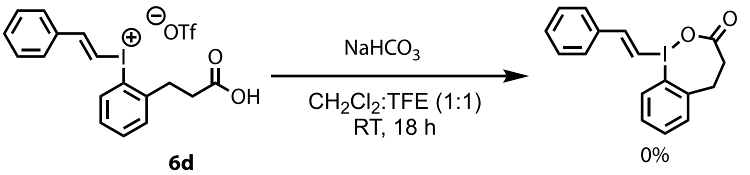

Iodonium salt **6d** (0.200 g, 0.35 mmol, 1.0 equiv) was added to a round bottom flask followed by CH_2Cl_2:TFE (1:1, 5 mL). Saturated $NaHCO_3$ was added and the mixture was stirred vigorously at room temperature for 16 h. The reaction mixture was transferred to a separation funnel, diluted with CH_2Cl_2 (20 mL) and H_2O (20 mL). Note: dilution helped to avoid emulsions in the separation. The layers were separated and the aqueous phase was extracted with CH_2Cl_2 (3 x 20 mL). The combined organic phases were washed with H_2O (20 mL) and brine (20 mL) and then dried over Na_2SO_4. The drying agent was filtered off and the solvent was removed in vacuo. Et_2O (50 mL) was added and the crude mixture was stirred for 30 min, and then stored in the freezer for 18 h. This resulted in no desired product whatsoever.

6 *S*-vinylation of bromothiophenol

6.1 Vinylation to yield (*E*)-(4-bromophenyl)(styryl)sulfane (14)

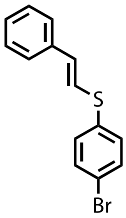

Following a modified version of a previously published procedure,[17] 4-bromothiophenol (18.9 mg, 0.10 mmol, 1.0 equiv) was placed in an oven-dried microwave vial with a magnetic stirring bar under inert atmosphere. To this was added anhydrous and degassed THF (0.66 mL). Then, vinylating reagent **1-7** (0.11 mmol, 1.1 equiv) and *t*BuOK (11.2 mg, 0.10 mmol, 1.0 equiv) were sequentially added and the vial was rinsed with THF (0.33 mL). The mixture rapidly turns yellow and it was stirred at room temperature for 2 h. The reaction was quenched with water (2.0 mL) and the aqueous phase was extracted with CH_2Cl_2 (2×10 mL) and the combined organic phases were dried over Na_2SO_4, filtered and concentrated under reduce pressure. 1,3,5-Trimethoxybenzene (16.8 mg, 0.1 mmol, 1.0 equiv) was added as internal standard for the purposes of NMR yield measurements. E/Z ratios varied for each reagent, but there was no trend.

R_f (2% EtOAc/pentane) 0.40;
δ_H (400 MHz, $CDCl_3$) 7.52 – 7.21 (9H, m), 6.80 (1H, d, *J* = 16.3 Hz), 6.75 (1H, d, *J* = 16.3 Hz);
δ_C (101 MHz, $CDCl_3$) 136.4, 134.8, 133.2, 132.4, 131.3, 128.9, 128.0, 126.3, 122.4, 121.0.
This data is consistent with literature precedent. [17]

Yields in main article and below are all NMR yields. Unless specified, all E/Z ratios were >19:1.

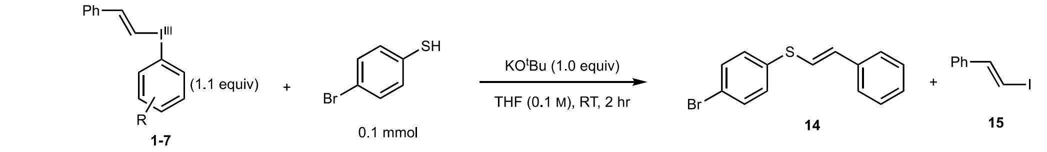

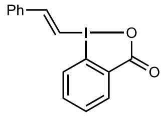

1a
87%

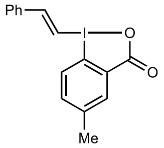

1b
77% (E:Z 11:1)

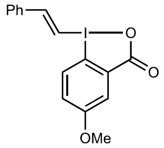

1c
75%

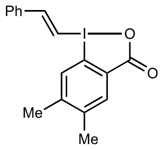

1d
90%

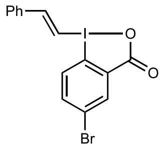

1e
67%

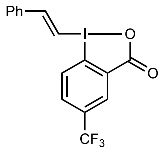

1f
64%

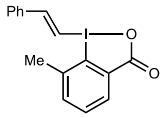

1g
68%

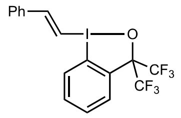

2a
45%

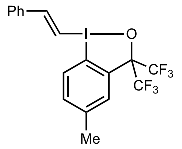

2b
40%

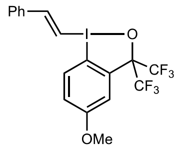

2c
33%

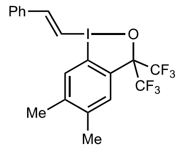

2d
35%

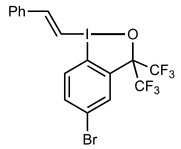

2e
51%

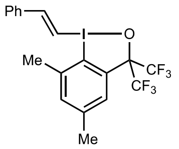

2f
30%
(50% of **15**)

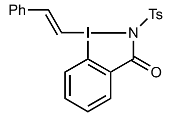

3a
80%

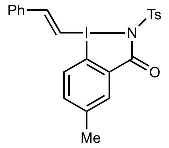

3b
85%

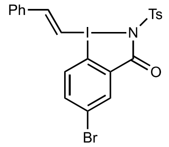

3c
90% (E:Z 3:1)
(10% of **15**)

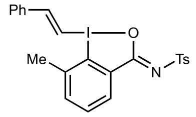

4
15%
(70% of **15**)

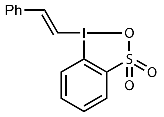

5a
32%

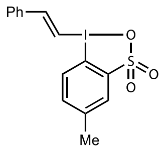

5b
35%

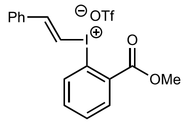

6a
36% (E:Z 9:1)
(22% of **15**)

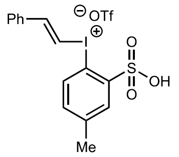

6b
15%

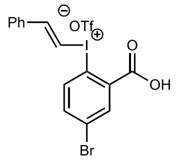

6c
22% (20% of **15**)

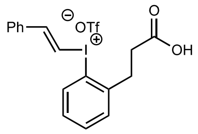

6d
16% (E:Z 1.5:1)
(23% of **15**)

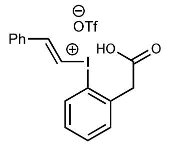

6e
12% (E:Z 1.5:1)
(20% of **15**)

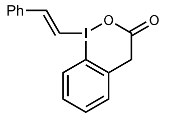

7
21% (E:Z 2.5:1)

7 Crystal structure determination

Single crystal X-ray diffraction data on suitable crystals of compounds **1d, 2b, 2e, 3a, 3b, 3c, 3d** and **5a** were collected using Cu Kα radiation on a Bruker D8 VENTURE diffractometer equipped with a PHOTON 100 detector. The datasets were reduced and absorption correction was applied by the APEX3 suite. The crystal structures were solved and refined by SHELXT and SHELXL respectively.[23] Crystal structures were refined using full-matrix least-squares based on F^2 with all non-hydrogen atoms anisotropically defined. All hydrogen atoms were placed using a riding model. Crystals of **2e** contain solvent accessible voids, and the data were treated using the SQUEEZE procedure by PLATON.[24]

CCDC 2142664, 2142665, 2323460-2323464, 2326666, 2323461, and 2324988 contain the supplementary crystallographic data for this paper. These data can be obtained free of charge from The Cambridge Crystallographic Data Center via http://www.ccdc.cam.ac.uk/structures.

7.1.1 (*E*)-5-Bromo-1-styryl-1λ^3-benzo[*d*][1,2]iodaoxol-3(1*H*)-one (1e)

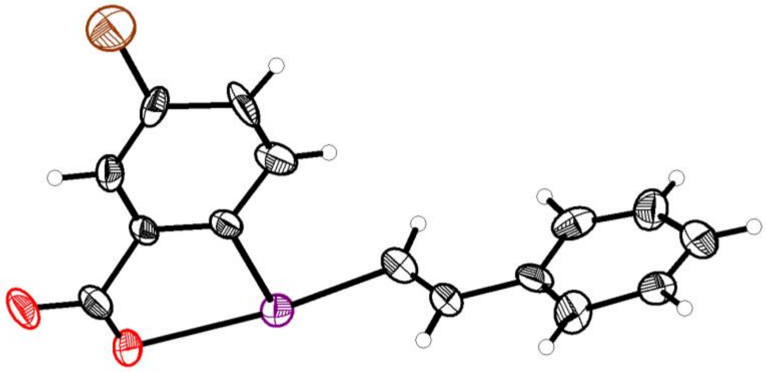

Figure S1. Structure of **1e** as determined by single crystal X-ray diffraction. Thermal ellipsoids are displayed with 50% probability. Atoms are coloured as the following: carbon (black), oxygen (red), iodine (violet), bromine (brown), hydrogen (white spheres). Single crystals were grown in a solution of DMSO/Et_2O.

Table S1: Crystallographic data and refinement details for compound **1e**.

deposition number	CCDC 2323464
empirical formula	C68 H60 Br4 I4 O10
formula weight	1864.40
temperature	296 K
wavelength	1.54178 Å
crystal system	monoclinic
space group	$P2_1$ (No. 4)
unit cell dimensions	*a* = 8.7132 (4) Å
	b = 20.9793 (9) Å
	c = 18.0746 (7) Å
	β = 98.547 (2) °
volume	3267.3 (2) Å^3
Z	2
density (calculated)	1.895 g/cm^3
absorption coefficient	4.411 mm^{-1}
F(000)	1800
θ range for data collection	2.251° to 30.594°
index ranges	$-12 \leq h \leq 12$, $-29 \leq k \leq 30$, $-25 \leq l \leq 25$
reflections collected	35410
independent reflections	20007 [*R*(int) = 0.0799]
absorption correction	multi-scan
data / restraints / parameters	20007 / 1 / 779
goodness-of-fit on F^2	0.974
final R indices [$I>2\sigma(I)$]	*R*1 = 0.0520, w*R*2 = 0.0746
largest diff. peak and hole	0.603 and –1.186 e/Å^3
Flack parameter	0.081 (14)

7.1.2 (E)-5-methyl-1-styryl-3,3-bis(trifluoromethyl)-1,3-dihydro-1λ3-benzo[*d*][1,2]-iodaoxole (2b)

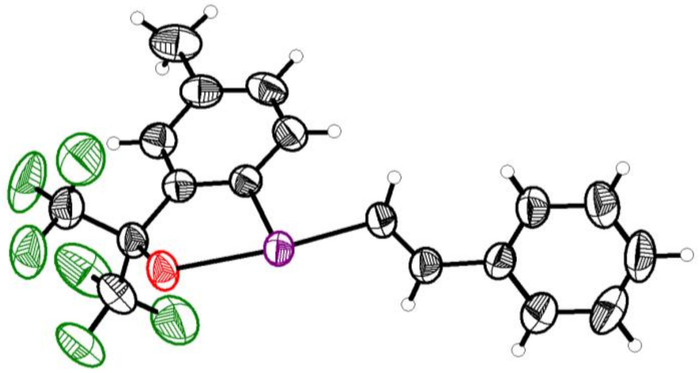

Figure S2. Structure of **2b** as determined by single crystal X-ray diffraction. Thermal ellipsoids are displayed with 50% probability. Atoms are coloured as the following: carbon (black), oxygen (red), iodine (violet), fluorine (green), hydrogen (white spheres). Single crystals were grown in a solution of DCM/Pentane.

Table S2: Crystallographic data and refinement details for compound **2b**.

deposition number	CCDC 2142664
empirical formula	C18 H13 F6 I O
formula weight	486.18
temperature	296 K
wavelength	1.54178 Å
crystal system	monoclinic
space group	$P2_1/c$ (No. 14)
unit cell dimensions	a = 11.7460 (5) Å
	b = 15.4664 (6) Å
	c = 9.9360 (4) Å
	β = 98.693 (2) °
volume	1784.32(13) Å^3
Z	4
density (calculated)	1.810 g/cm^3
absorption coefficient	1.858 mm^{-1}
F(000)	944
θ range for data collection	2.193° to 26.438°
index ranges	$-14\leq h\leq 14$, $-19\leq k\leq 19$, $-12\leq l\leq 12$
reflections collected	35410
independent reflections	3674 [R(int) = 0.0473]
absorption correction	multi-scan
data / restraints / parameters	3674 / 0 / 236
goodness-of-fit on F^2	1.037
final R indices [I>2σ(I)]	$R1$ = 0.0245, w$R2$ = 0.0503
largest diff. peak and hole	0.422 and –0.375 e/Å^3

(E)-5-bromo-1-styryl-3,3-bis(trifluoromethyl)-1,3-dihydro-1λ^3-benzo[*d*][1,2]iodaoxole (2e)

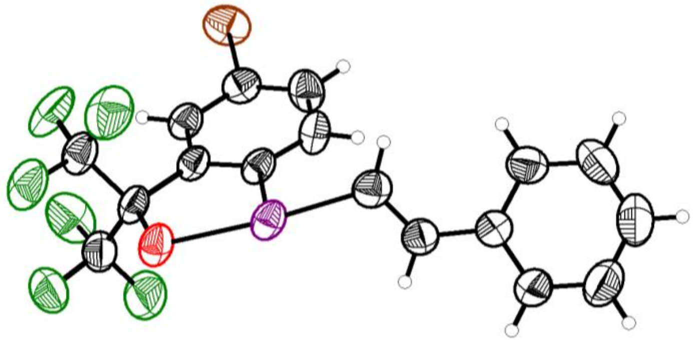

Figure S3. Structure of **2e** as determined by single crystal X-ray diffraction. Thermal ellipsoids are displayed with 50% probability. Atoms are coloured as the following: carbon (black), oxygen (red), iodine (violet), fluorine (green), bromine (brown), hydrogen (white spheres). Single crystals were grown in a solution of DCM/Pentane.

Table S3: Crystallographic data and refinement details for compound **2e**.

deposition number	CCDC 2142665
empirical formula	C17 H10 Br F6 I O
formula weight	551.06
temperature	296 K
wavelength	1.54178 Å
crystal system	triclinic
space group	*P*-1 (No. 2)
unit cell dimensions	*a* = 9.8631 (4) Å
	b = 10.0243 (6) Å
	c = 21.6891 (10) Å
	α = 77.871 (2) °
	β = 77.122 (1) °
	γ = 70.568 (1) °
volume	1949.72 (17) Å^3
Z	4
density (calculated)	1.877 g/cm^3
absorption coefficient	3.749 mm^{-1}
F(000)	1048
θ range for data collection	2.220° to 25.350°
index ranges	$-11 \leq h \leq 11$, $-12 \leq k \leq 12$, $-26 \leq l \leq 26$
reflections collected	60315
independent reflections	7121 [*R*(int) = 0.0420]
absorption correction	multi-scan
data / restraints / parameters	7121 / 0 / 469
goodness-of-fit on F^2	1.030
final R indices [$I>2\sigma(I)$]	*R*1 = 0.0272, w*R*2 = 0.0654
largest diff. peak and hole	1.229 and –0.715e/Å^3

7.1.3 (E)-1-styryl-2-tosyl-1,2-dihydro-3H-1λ^3-benzo[*d*][1,2]iodazol-3-one (3a)

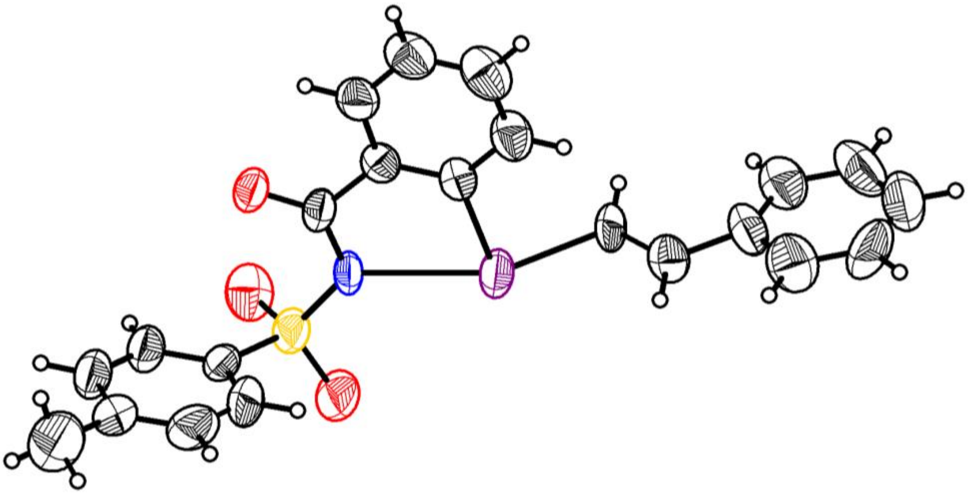

Figure S4. Structure of **3a** as determined by single crystal X-ray diffraction. Thermal ellipsoids are displayed with 50% probability. Atoms are coloured as the following: carbon (black), oxygen (red), nitrogen (blue), iodine (violet), sulfur (yellow), hydrogen (white spheres). Single crystals were grown in a solution of boiling DCM.

Table S4: Crystallographic data and refinement details for compound **3a**.

deposition number	CCDC 2323460
empirical formula	C22 H18 I N O3 S
formula weight	503.33
temperature	296 K
wavelength	1.54178 Å
crystal system	orthorhombic
space group	*Pbca* (No. 61)
unit cell dimensions	a = 14.6515 (8) Å
	b = 12.7501 (7) Å
	c = 22.0773 (11) Å
volume	4124.2 (4) Å^3
Z	8
density (calculated)	1.621 g/cm^3
absorption coefficient	1.677 mm^{-1}
F(000)	2000
θ range for data collection	2.310° to 28.347°
index ranges	$-19 \le h \le 19$, $-15 \le k \le 16$, $-29 \le l \le 29$
reflections collected	29954
independent reflections	5127 [R(int) = 0.0859]
absorption correction	multi-scan
data / restraints / parameters	5127 / 0 / 272
goodness-of-fit on F^2	1.112
final R indices [I>2σ(I)]	R1 = 0.0892, wR2 = 0.1354
largest diff. peak and hole	0.747and –0.997 e/Å^3

7.1.4 (*E*)-5-methyl-1-styryl-2-tosyl-1,2-dihydro-3H-1λ^3-benzo[*d*][1,2]iodazol-3-one (3b)

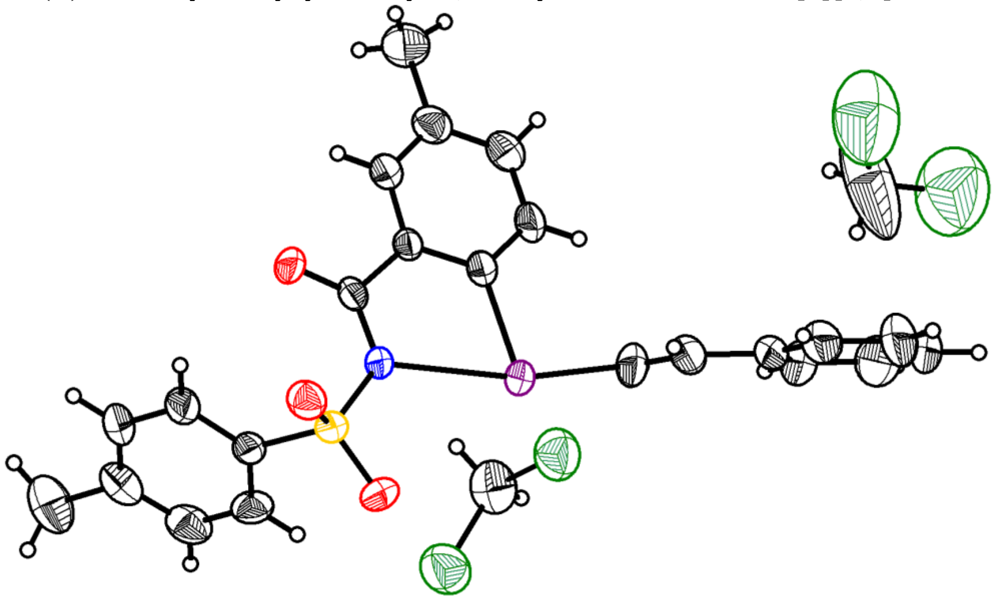

Figure S5. Structure of **3b** as determined by single crystal X-ray diffraction. Thermal ellipsoids are displayed with 50% probability. Atoms are coloured as the following: carbon (black), oxygen (red), nitrogen (blue), iodine (violet), sulfur (yellow), chlorine (green), hydrogen (white spheres). Single crystals were grown in a solution of boiling DCM.

Table S5: Crystallographic data and refinement details for compound **3b**.

deposition number	CCDC 2323463
empirical formula	C25 H24 Cl4 I N O3 S
formula weight	687.21
temperature	296 K
wavelength	1.54178 Å
crystal system	monoclinic
space group	$P2_1/c$ (No. 14)
unit cell dimensions	a = 13.5899 (15) Å
	b = 18.285 (2) Å
	c = 12.6827 (14) Å
	β = 115.502 (3) °
volume	2844.5 (5) Å^3
Z	4
density (calculated)	1.605 g/cm^3
absorption coefficient	1.602 mm^{-1}
F(000)	1368
θ range for data collection	2.779° to 28.370°
index ranges	$-18 \le h \le 18$, $-24 \le k \le 24$, $-16 \le l \le 16$
reflections collected	60712
independent reflections	7083 [*R*(int) = 0.2089]
absorption correction	multi-scan
data / restraints / parameters	7083 / 0 / 318
goodness-of-fit on F^2	1.112
final R indices [I>2σ(I)]	$R1$ = 0.0802, w$R2$ = 0.1659
largest diff. peak and hole	1.241 and –1.671 e/Å^3

7.1.5 (*E*)-5-bromo-1-styryl-2-tosyl-1,2-dihydro-3H-1λ^3-benzo[*d*][1,2]iodazol-3-one (3c)

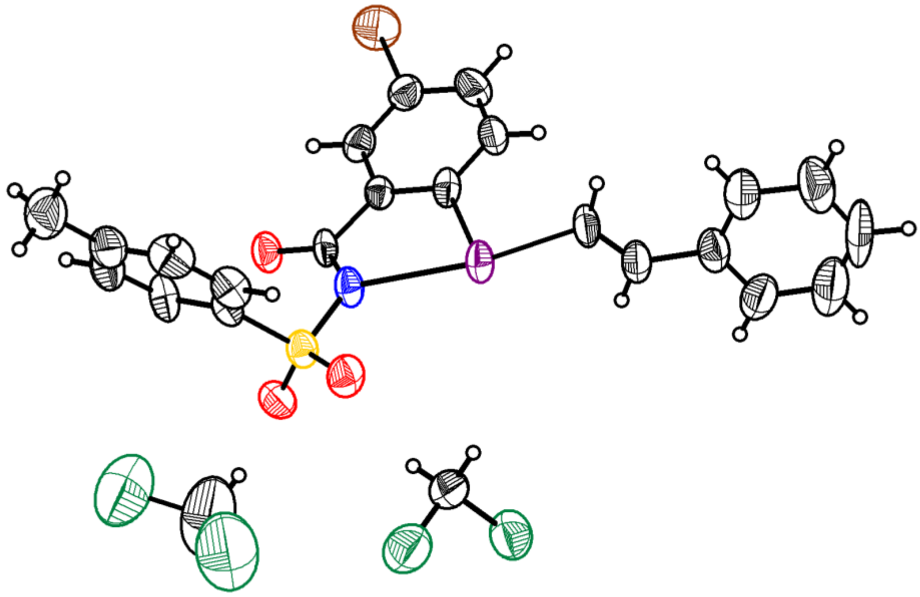

Figure S6. Structure of **3c** as determined by single crystal X-ray diffraction. Thermal ellipsoids are displayed with 50% probability. Atoms are coloured as the following: carbon (black), oxygen (red), nitrogen (blue), iodine (violet), sulfur (yellow), bromine (brown), chlorine (green), hydrogen (white spheres). Single crystals were grown in a solution of boiling DCM.

Table S6: Crystallographic data and refinement details for compound **3c**.

deposition number	CCDC 2323462
empirical formula	C24 H21 Br Cl4 I N O3 S
formula weight	752.09
temperature	296 K
wavelength	1.54178 Å
crystal system	monoclinic
space group	$P2_1/c$ (No. 14)
unit cell dimensions	a = 13.642 (2) Å
	b = 18.156 (2) Å
	c = 12.6844 (17) Å
	β = 115.170 (4)°
volume	2843.4 (7) Å^3
Z	4
density (calculated)	1.757 g/cm^3
absorption coefficient	3.008 mm^{-1}
F(000)	1472
θ range for data collection	1.995° to 26.373°
index ranges	$-15\leq h\leq 17$, $-22\leq k\leq 22$, $-15\leq l\leq 15$
reflections collected	36399
independent reflections	5803 [R(int) = 0.1266]
absorption correction	multi-scan
data / restraints / parameters	5803 / 0 / 317
goodness-of-fit on F^2	1.085
final R indices [$I>2\sigma(I)$]	$R1$ = 0.0662, w$R2$ = 0.1286
largest diff. peak and hole	1.143 and –0.882 e/Å^3

7.1.6 (*E*)-4-methyl-*N*-((*Z*)-7-methyl-1-((*E*)-styryl)-1λ^3 -benzo[*d*][1,2]iodaoxol-3(1*H*)-ylidene)benzenesulfonamide (4)

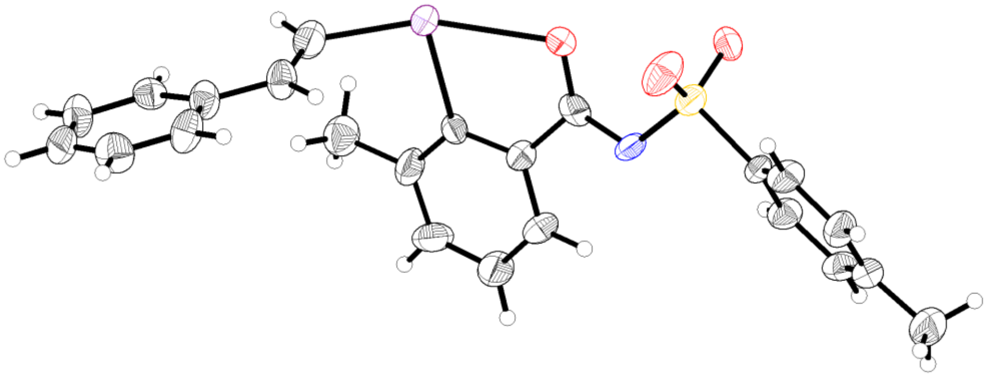

Figure S7. Structure of **4** as determined by single crystal X-ray diffraction. Thermal ellipsoids are displayed with 50% probability. Atoms are coloured as the following: carbon (black), oxygen (red), nitrogen (blue), iodine (violet), sulfur (yellow), hydrogen (white spheres). Single crystals were grown in a solution of DCM/Pentane.

Table S7: Crystallographic data and refinement details for compound **4**.

deposition number	CCDC 2323811
empirical formula	C23 H20 I N O3 S
formula weight	517.36
temperature	296 K
wavelength	0.71073
crystal system	orthorhombic
space group	*Pbca* (No. 61)
unit cell dimensions	*a* = 17.947 (3) Å
	b = 11.4958 (19) Å
	c = 20.864 (5) Å
volume	4304.7 (14) Å^3
Z	8
density (calculated)	1.597 g/cm^3
absorption coefficient	1.609 mm^{-1}
F(000)	2064
θ range for data collection	2.258 ° to 25.399 °
index ranges	$-21 \leq h \leq 21$, $-13 \leq k \leq 13$, $-25 \leq l \leq 25$
reflections collected	28723
independent reflections	3955 [*R*(int) = 0.3617]
absorption correction	multi-scan
data / restraints / parameters	4882 / 0 / 264
goodness-of-fit on F^2	1.013
final R indices [$I > 2\sigma(I)$]	*R*1 = 0.0739, w*R*2 = 0.1180
largest diff. peak and hole	0.753 and -0.710 e/Å^3

7.1.7 (*E*)-1-styryl-1H-1λ^3-benzo[*d*][1,2,3]iodaoxathiole 3,3-dioxide (5a)

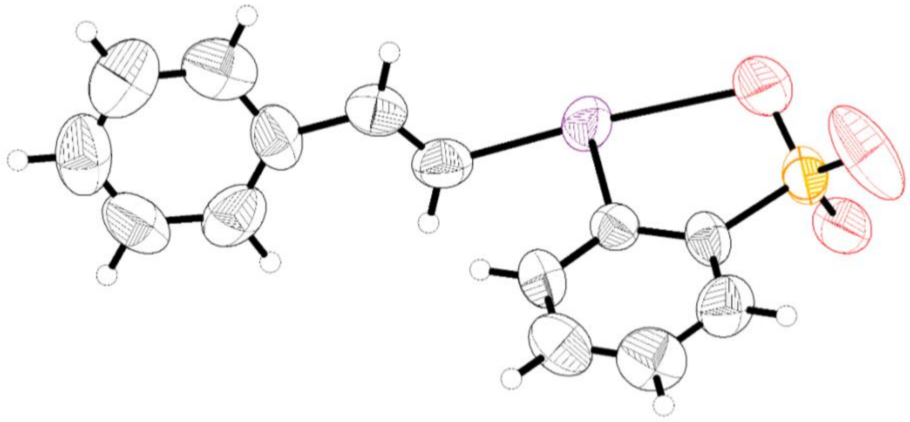

Figure S8. Structure of **5a** as determined by single crystal X-ray diffraction. Thermal ellipsoids are displayed with 50% probability. The structure has positional disorder in the vinyl and sulfonate groups which was modelled by split occupancies in the carbon and oxygen positions. Only the carbon and oxygen positions with the higher occupancies are displayed here for clarity. Atoms are colored as the following: carbon (black), oxygen (red), iodine (violet), sulfur (yellow), hydrogen (white spheres). Single crystals were grown in a solution of boiling MeCN.

Table S8: Crystallographic data and refinement details for compound **5a**.

deposition number	CCDC 2326666
empirical formula	C14 H11 I O3 S
formula weight	386.19
temperature	296 K
wavelength	0.71073 Å
crystal system	monoclinic
space group	$P2_1/c$ (No. 14)
unit cell dimensions	a = 9.1334 (6) Å
	b = 14.5161 (13) Å
	c = 10.4124 (10) Å
	β = 90.569 (3) °
volume	1380.4 (2) Å^3
Z	4
density (calculated)	1.858 g/cm^3
absorption coefficient	2.471 mm^{-1}
F(000)	752
θ range for data collection	2.407 ° to 25.351 °
index ranges	$-10 \leq h \leq 11$, $-17 \leq k \leq 17$, $-12 \leq l \leq 12$
reflections collected	8603
independent reflections	2519 [R(int) = 0.0544]
absorption correction	multi-scan
data / restraints / parameters	2519 / 18 / 219
goodness-of-fit on F^2	1.070
final R indices [$I>2\sigma(I)$]	$R1$ = 0.0455, w$R2$ = 0.0912
largest diff. peak and hole	1.045 and -1.026 e/Å^3

7.1.8 (*E*)-(2-(methoxycarbonyl)phenyl)(styryl)iodonium trifluoromethanesulfonate (6a)

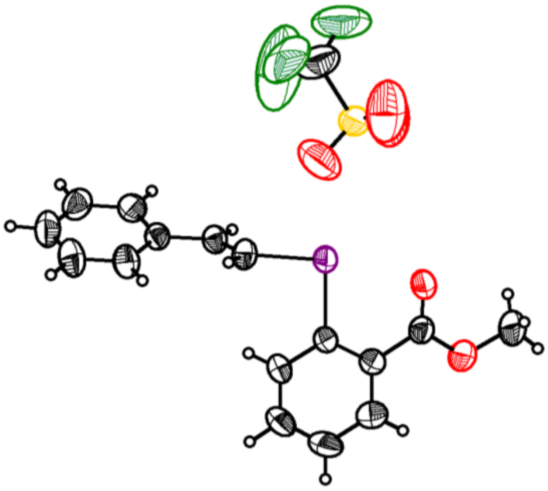

Figure S8: Structure of **6a** as determined by single crystal X-ray diffraction. Thermal ellipsoids are displayed with 50% probability. Atom are coloured as the following: carbon (black), oxygen (red), iodine (violet), sulfur (yellow), fluorine (green), hydrogen (white spheres). Single crystals were grown in a solution of DCM/Et_2O.

Table S8: Crystallographic data and refinement details for compound **6a**.

deposition number	CCDC 2323461
empirical formula	C17 H14 F3 I O5 S
formula weight	514.24
temperature	296 K
wavelength	1.54178 Å
crystal system	triclinic
space group	*P*-1 (No. 2)
unit cell dimensions	*a* = 9.2089 (17) Å
	b = 10.4808 (19)Å
	c = 11.656 (2)Å
	α = 109.199 (7) °
	β = 96.235 (8)°
	γ = 108.745 (8)°
volume	976.8 (3)$Å^3$
Z	2
density (calculated)	1.748 g/cm^3
absorption coefficient	1.798 mm^{-1}
F(000)	504
θ range for data collection	2.225 ° to 28.423 °
index ranges	$-12 \leq h \leq 12$, $-13 \leq k \leq 13$, $-15 \leq l \leq 15$
reflections collected	31705
independent reflections	4882 [*R*(int) = 0.0414]
absorption correction	multi-scan
data / restraints / parameters	4882 / 0 / 245
goodness-of-fit on F^2	1.035
final R indices [$I>2\sigma(I)$]	*R*1 = 0.0434, w*R*2 = 0.1222
largest diff. peak and hole	1.197 and -1.183 e/$Å^3$

7.1.10 (*E*)-1-styryl-1,4-dihydro-3H-1λ^3-benzo[*d*][1,2]iodaoxine-3-one (7)

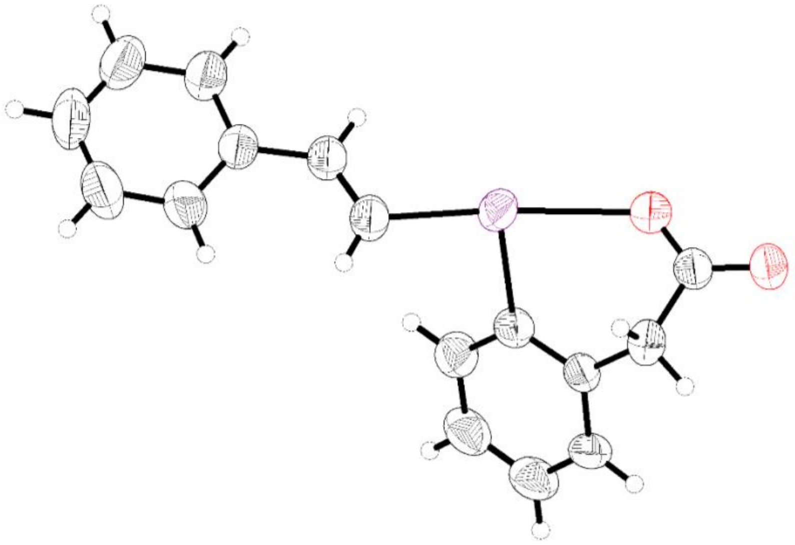

Figure S10: Structure of **7** as determined by single crystal X-ray diffraction. Thermal ellipsoids are displayed with 50% probability. Atom are coloured as the following: carbon (black), oxygen (red), iodine (violet), hydrogen (white spheres). Single crystals were grown in a solution of boiling MeCN.

Table S10: Crystallographic data and refinement details for compound **7**.

deposition number	CCDC 2324988
empirical formula	C16 H13 I O2
formula weight	364.16
temperature	296 K
wavelength	0.71073 Å
crystal system	monoclinic
space group	$P2_1/c$ (No. 14)
unit cell dimensions	a = 8.954 (2) Å
	b = 10.454 (3) Å
	c = 15.365 (4) Å
	β = 102.560 (6) °
volume	1403.8 (6) Å^3
Z	4
density (calculated)	1.723 g/cm^3
absorption coefficient	2.276 mm^{-1}
F(000)	712
θ range for data collection	2.375 ° to 26.416 °
index ranges	$-11 \le h \le 11$, $-13 \le k \le 13$, $-19 \le l \le 19$
reflections collected	31047
independent reflections	2873 [R(int) = 0.0888]
absorption correction	multi-scan
data / restraints / parameters	2873 / 0 / 172
goodness-of-fit on F^2	1.003
final R indices [$I>2\sigma(I)$]	$R1$ = 0.0342, w$R2$ = 0.0812
largest diff. peak and hole	0. 501 and -0.368 e/Å^3

8 Electrochemical analysis

8.1 Electrode potential setup

All cyclic voltammetric (CV) measurements were performed at room temperature using an Potentiostat/Galvanostat/ZRA Gamry Interface 1010E (EC lab). CV experiments were carried out with a working electrode (GC = glassy carbon), a counter electrode (platinum wire) and a reference electrode (Ag/AgCl (3.0 M KCl). All measurements were then corrected to Fc/Fc^+. All working electrodes were polished before each experiment. After each CV, the solution was stirred for approximately 10 seconds, whilst being degassed by a stream of N_2.

8.2 Cyclic voltametric plots

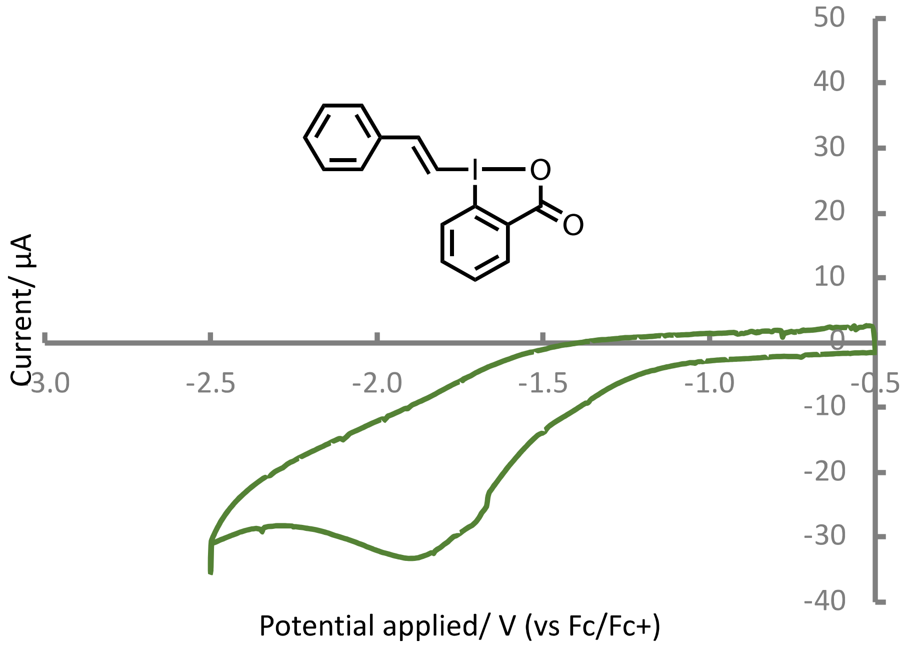

Figure S11. CV of **1a.**

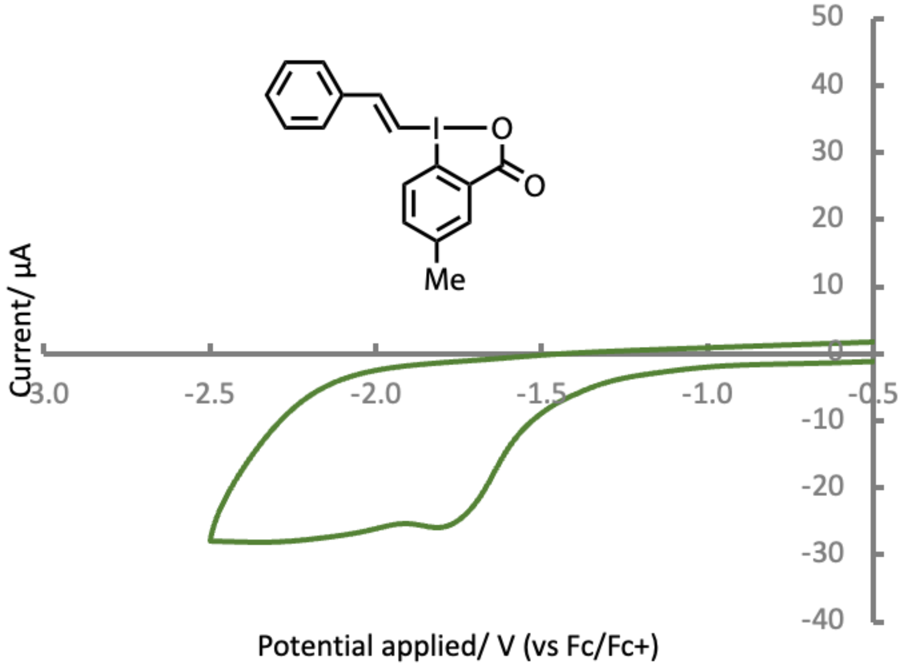

Figure S12. CV of **1b.**

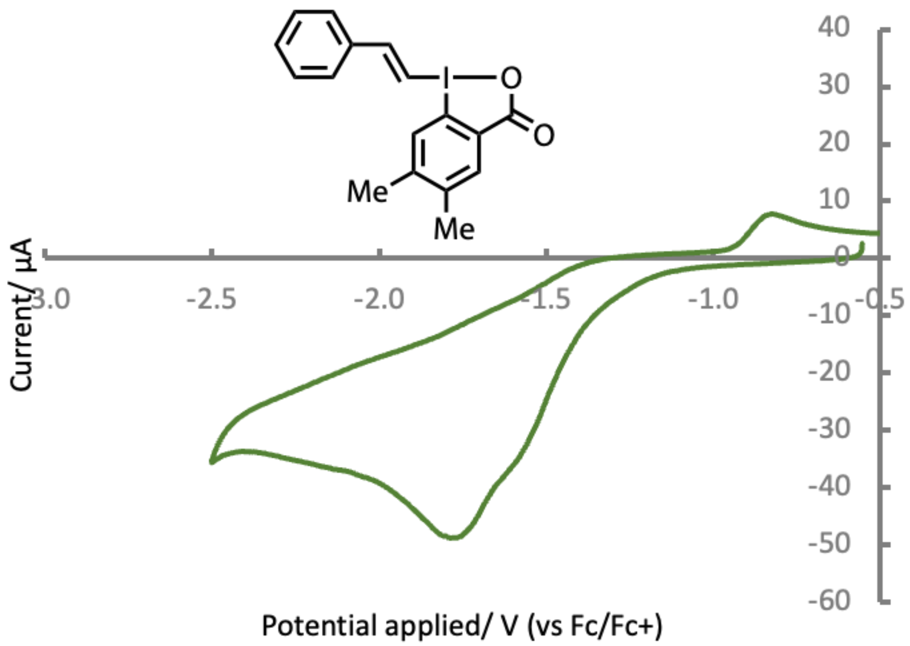

Figure S13. CV of **1c.**

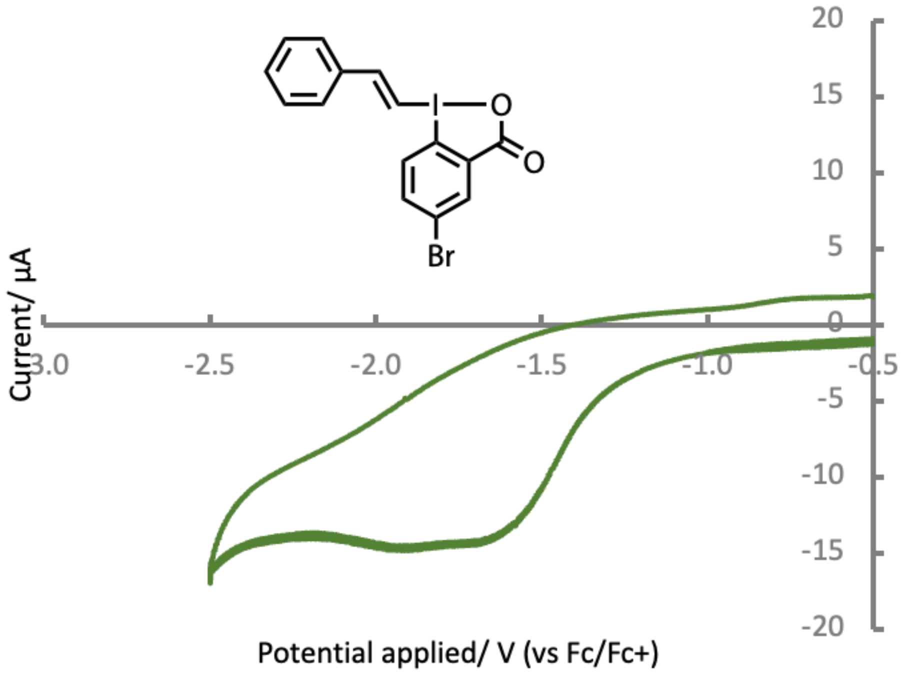

Figure S14. CV of **1d.**

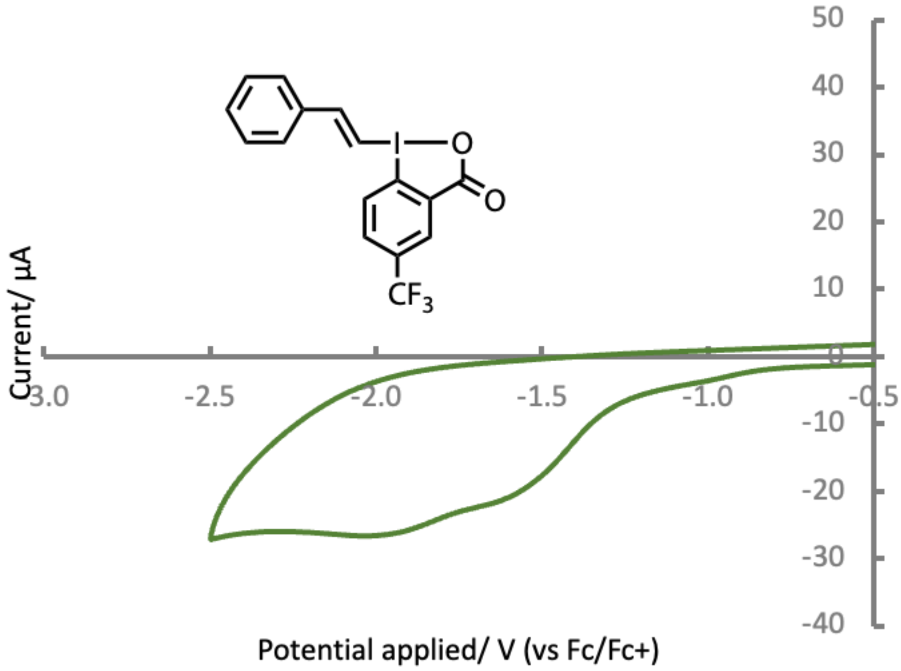

Figure S15. CV of **1e.**

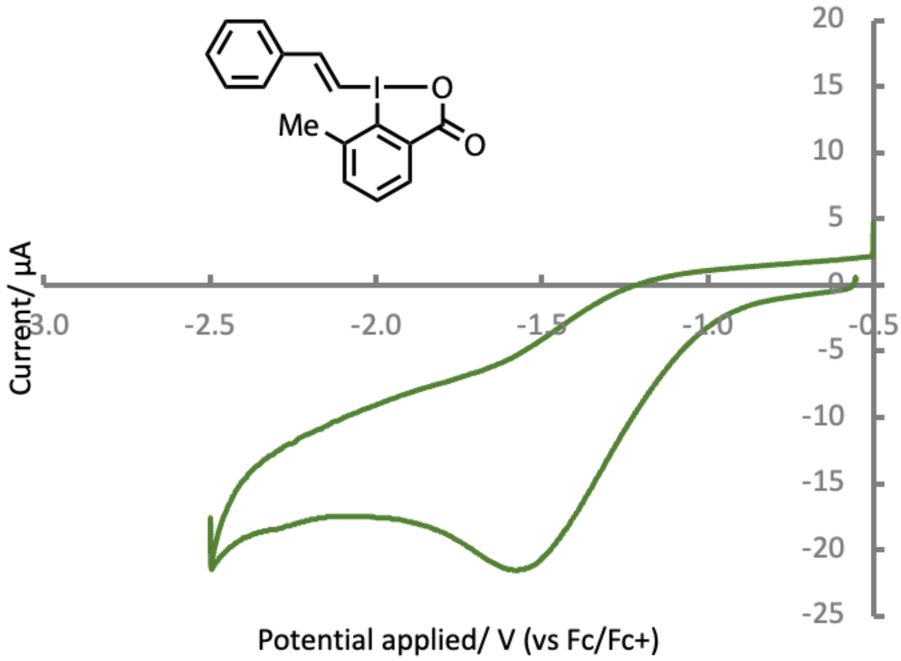

Figure S16. CV of **1f.**

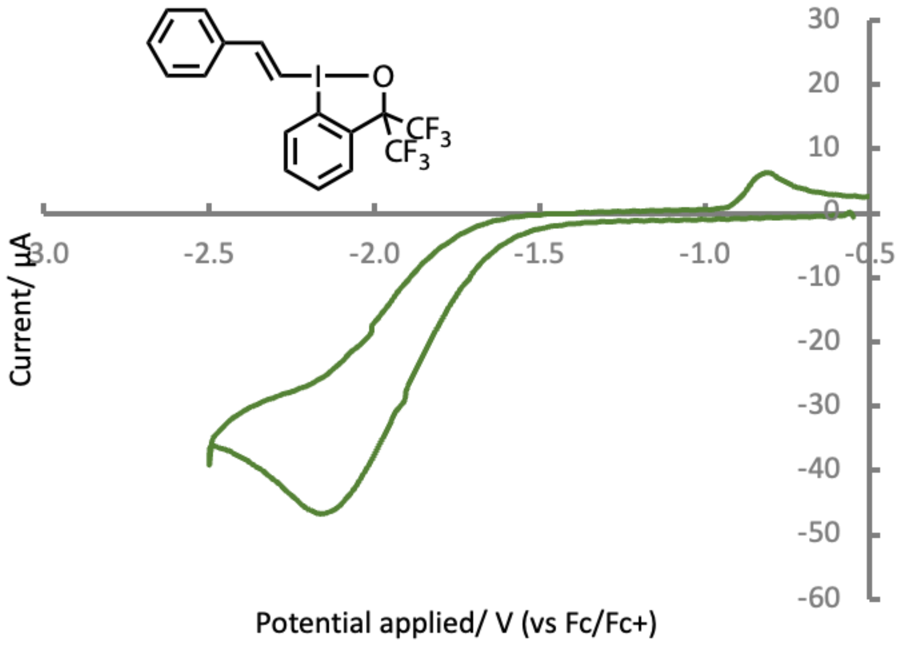

Figure S17. CV of **2a.**

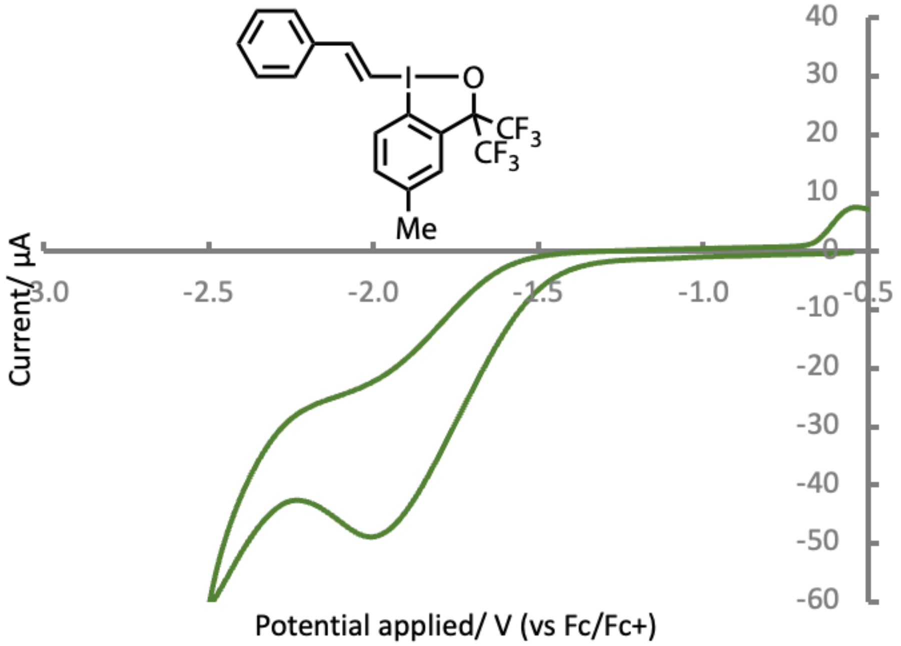

Figure S18. CV of **2b.**

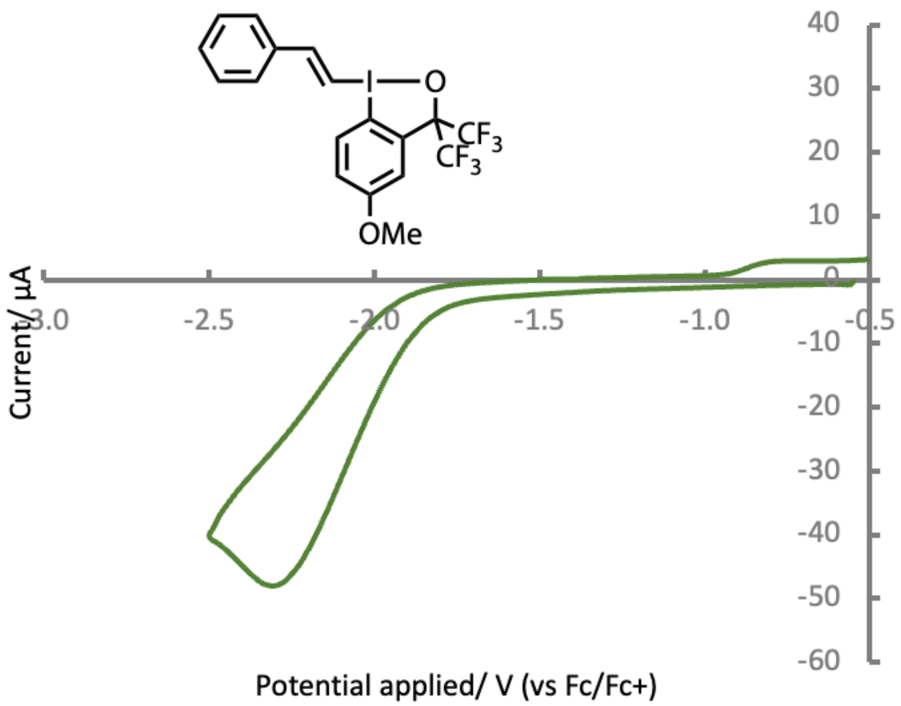

Figure S19. CV of **2c.**

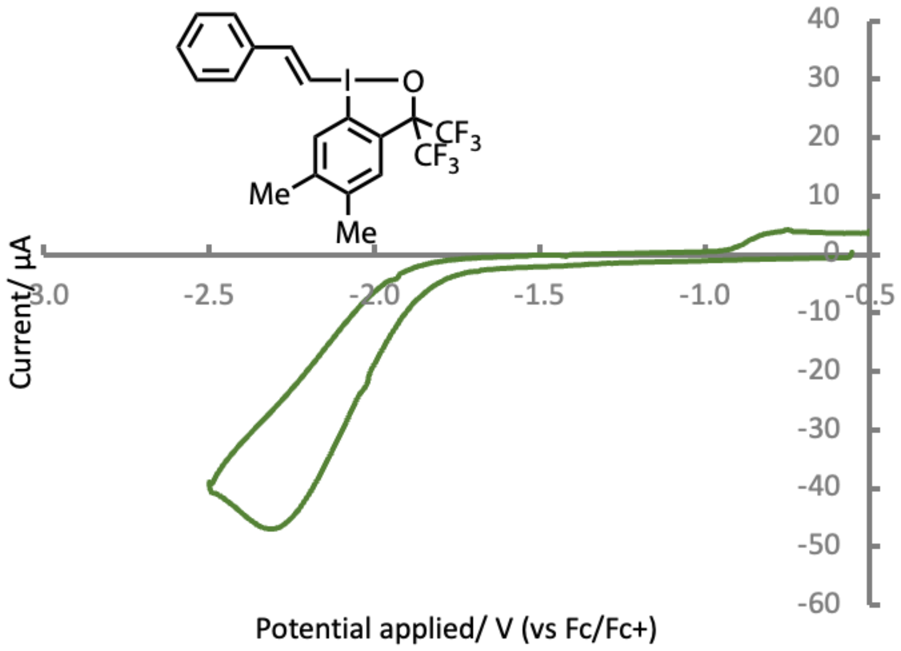

Figure S20. CV of **2d.**

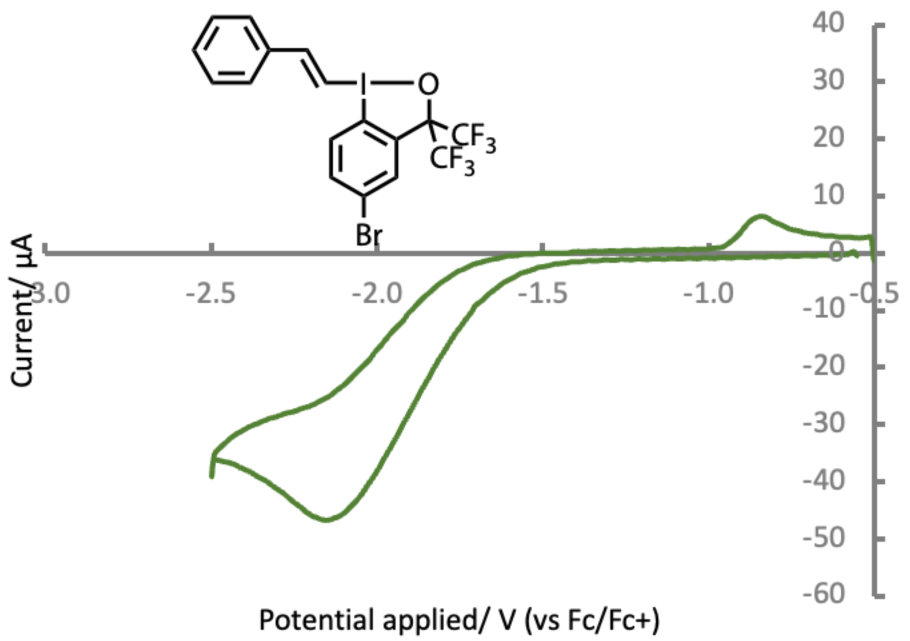

Figure S21. CV of **2e.**

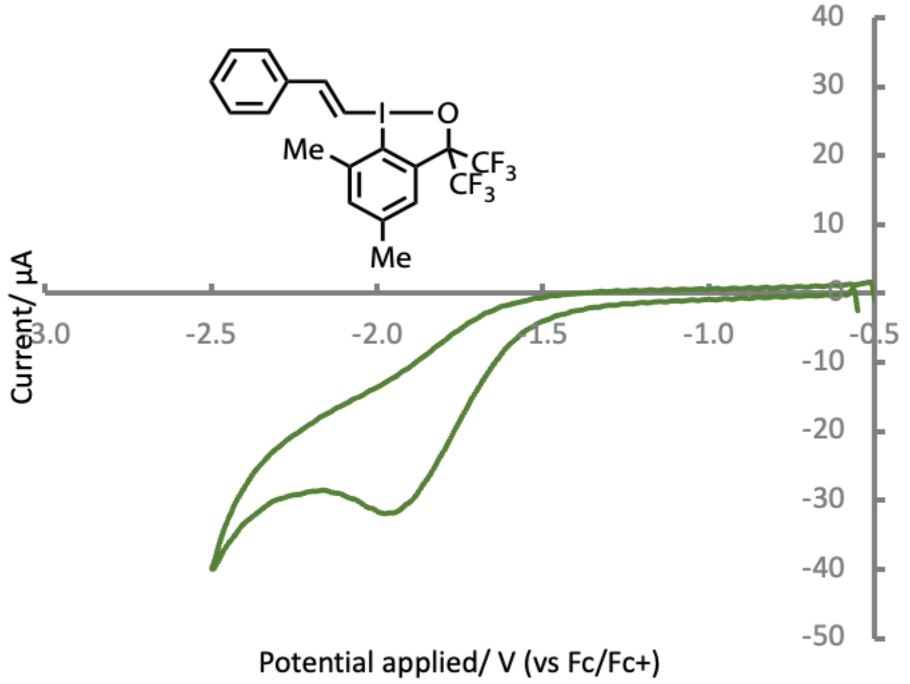

Figure S22. CV of **2f.**

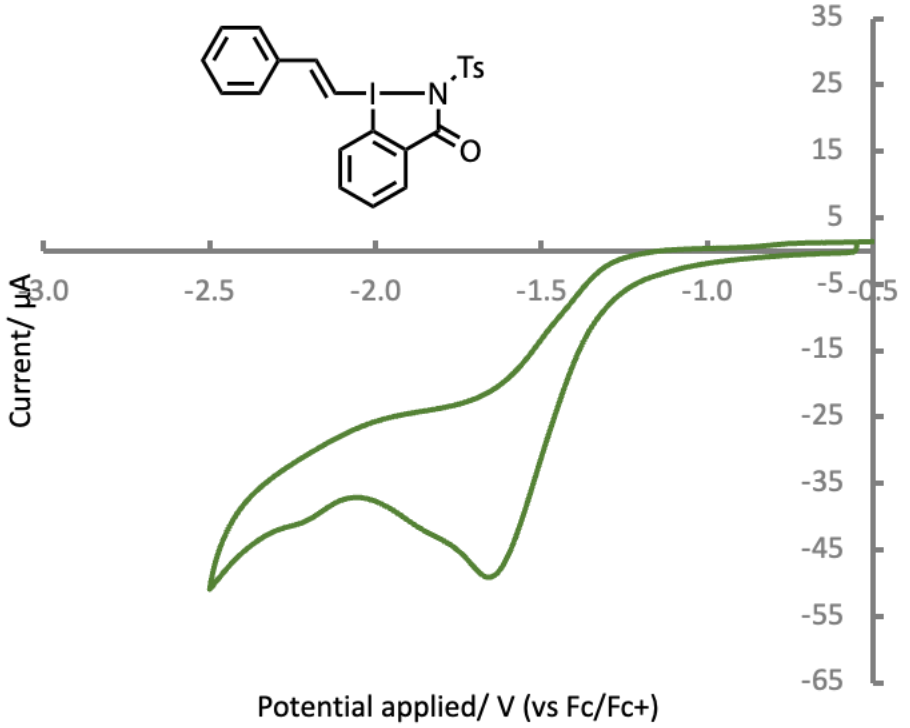

Figure S23. CV of **3a.**

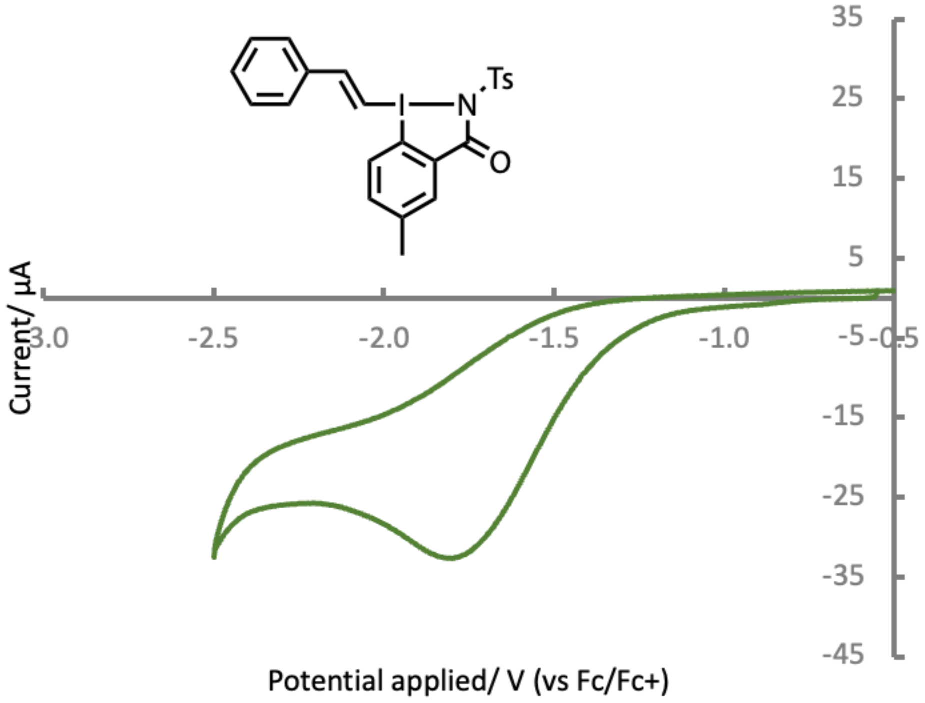

Figure S24. CV of **3b.**

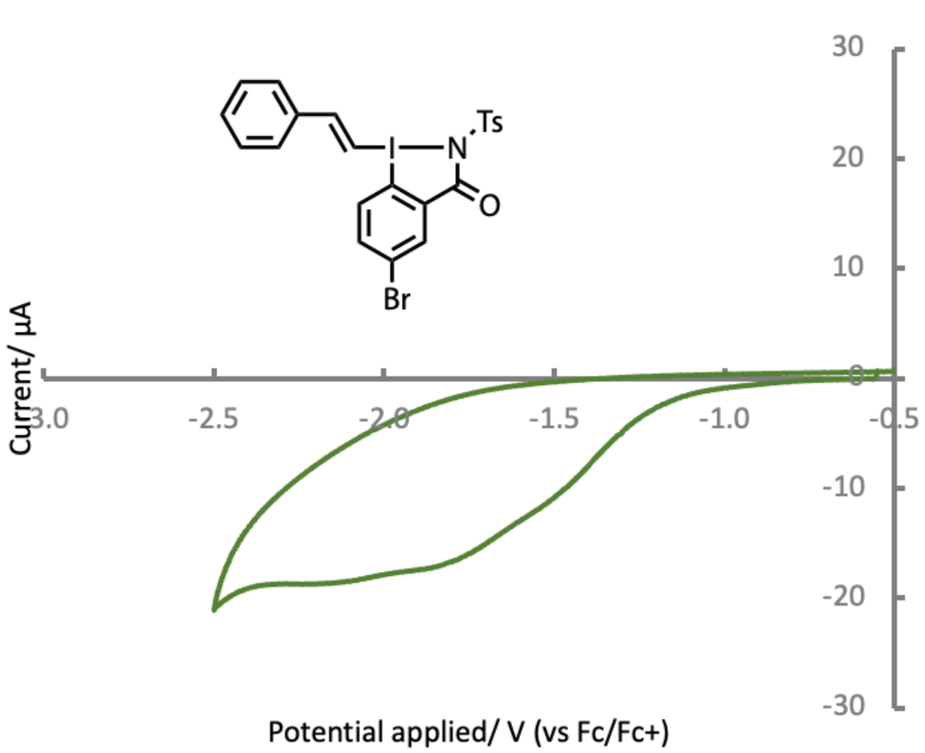

Figure S25. CV of **3c.**

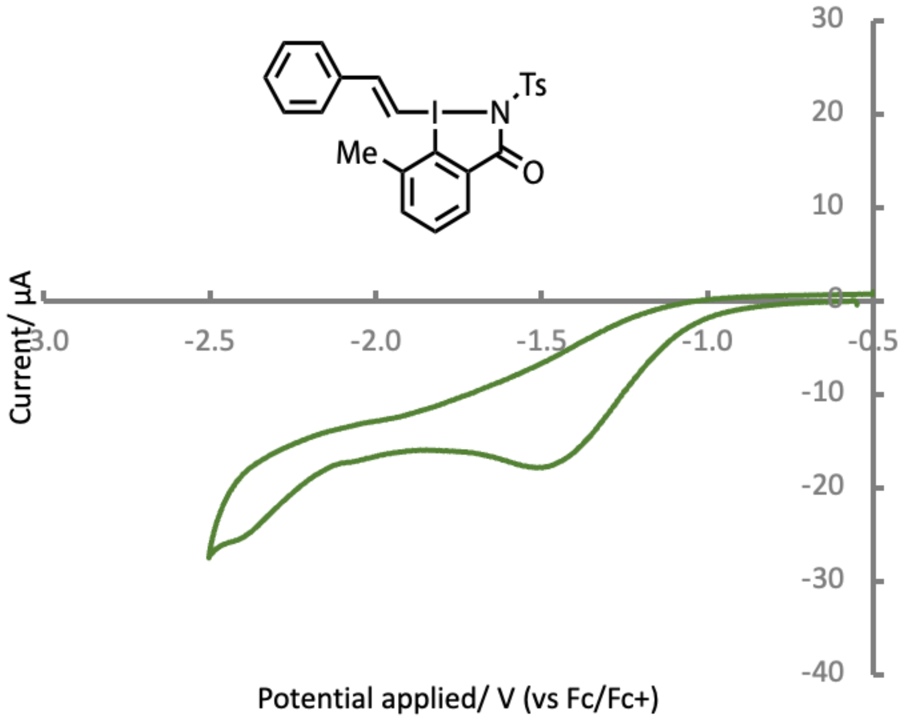

Figure S26. CV of **4.**

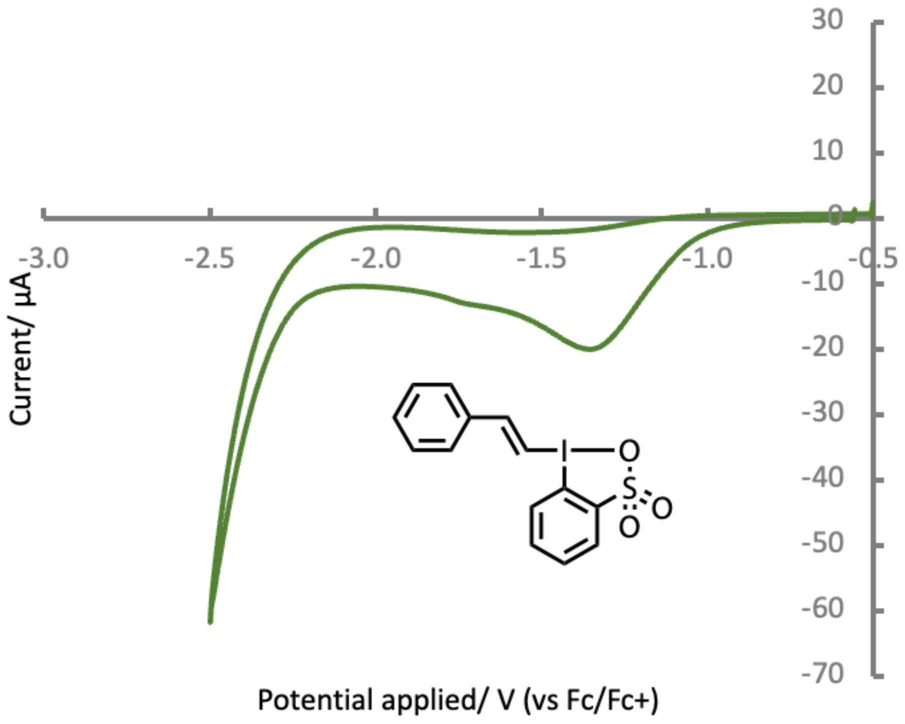

Figure S27. CV of **5a.**

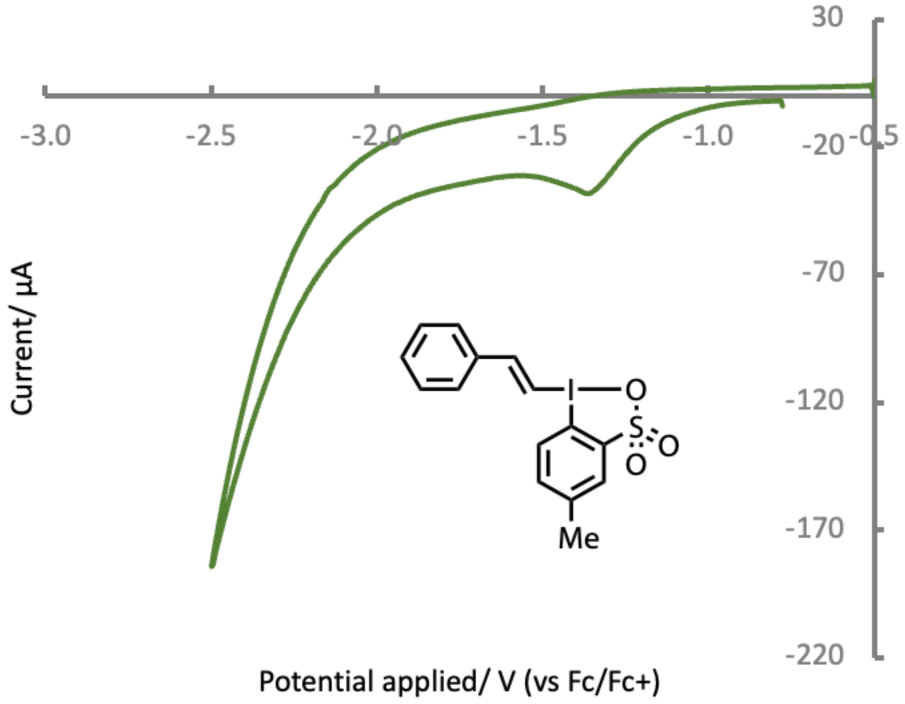

Figure S28. CV of **5b.**

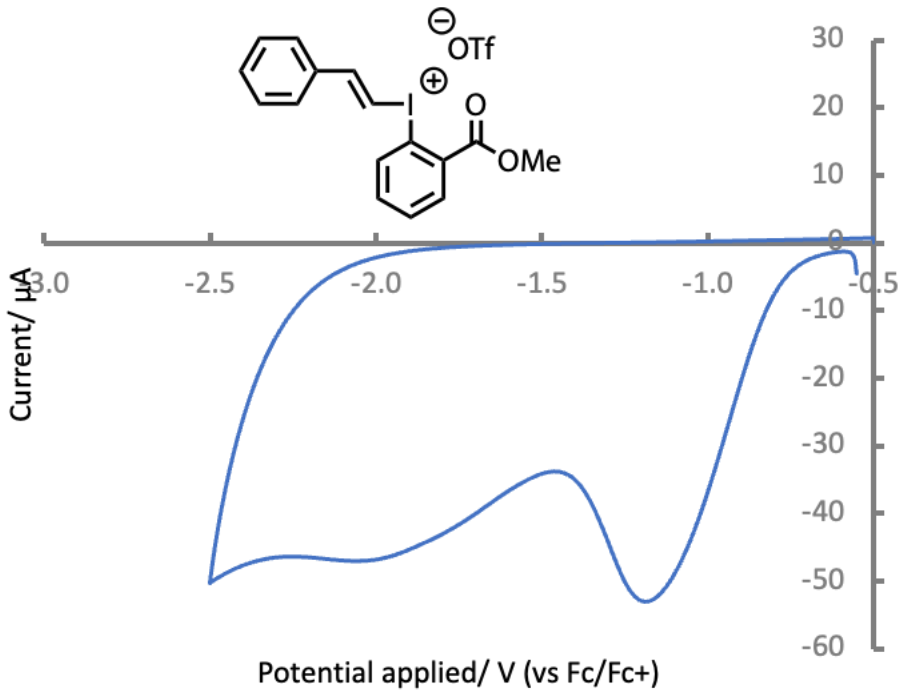

Figure S29. CV of **6a.**

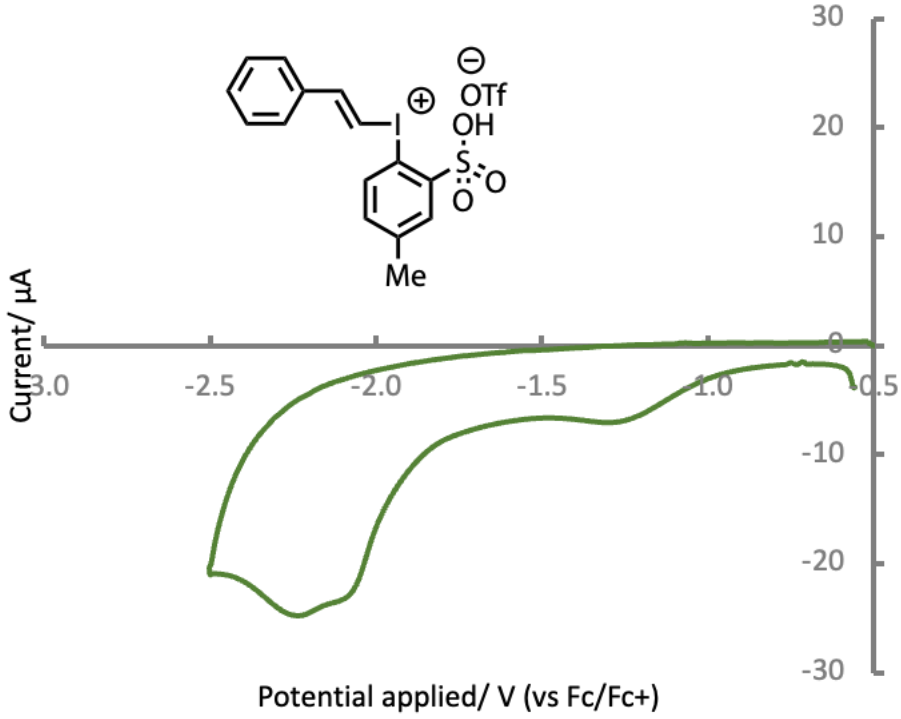

Figure S30. CV of **6b.**

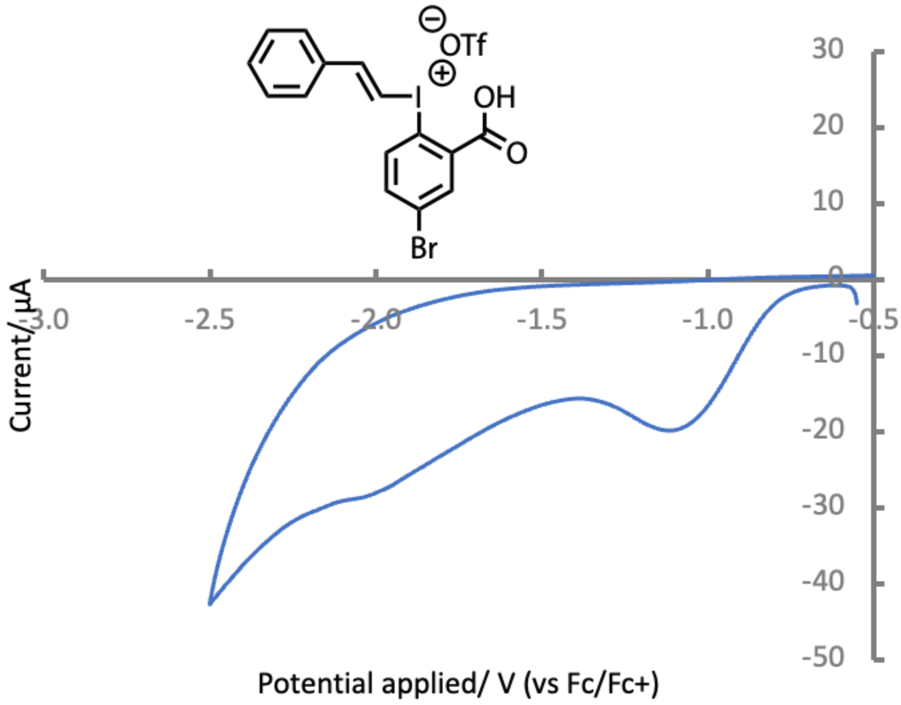

Figure S31. CV of **6c.**

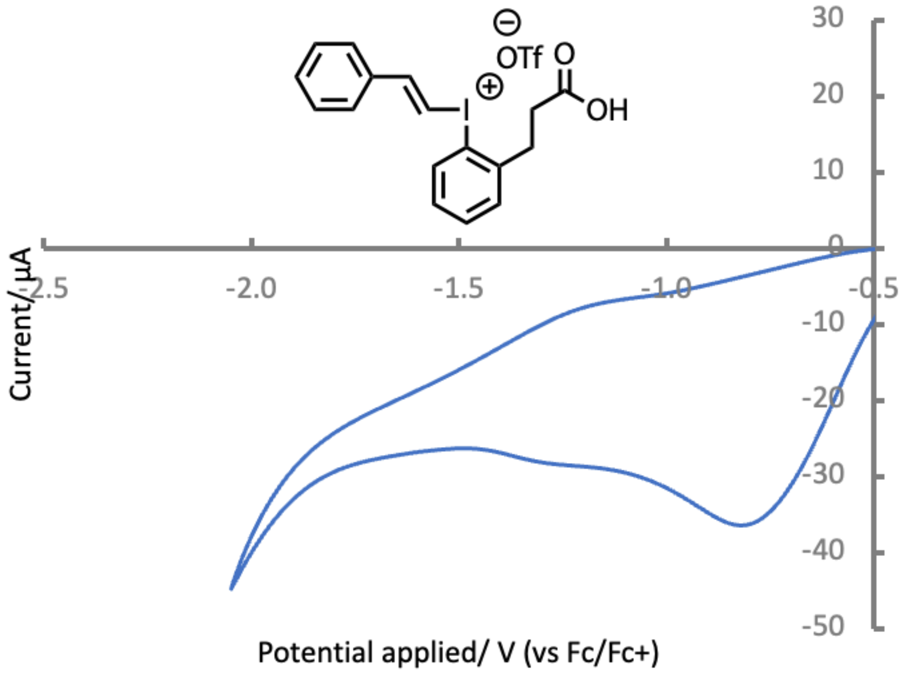

Figure S32. CV of **6d.**

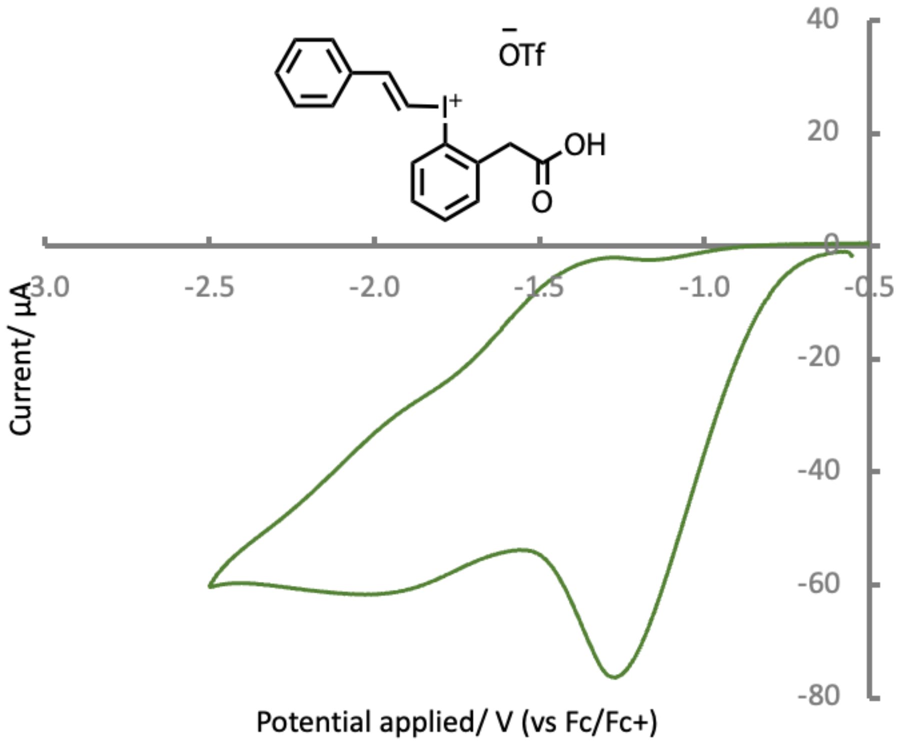

Figure S33. CV of **6e.**

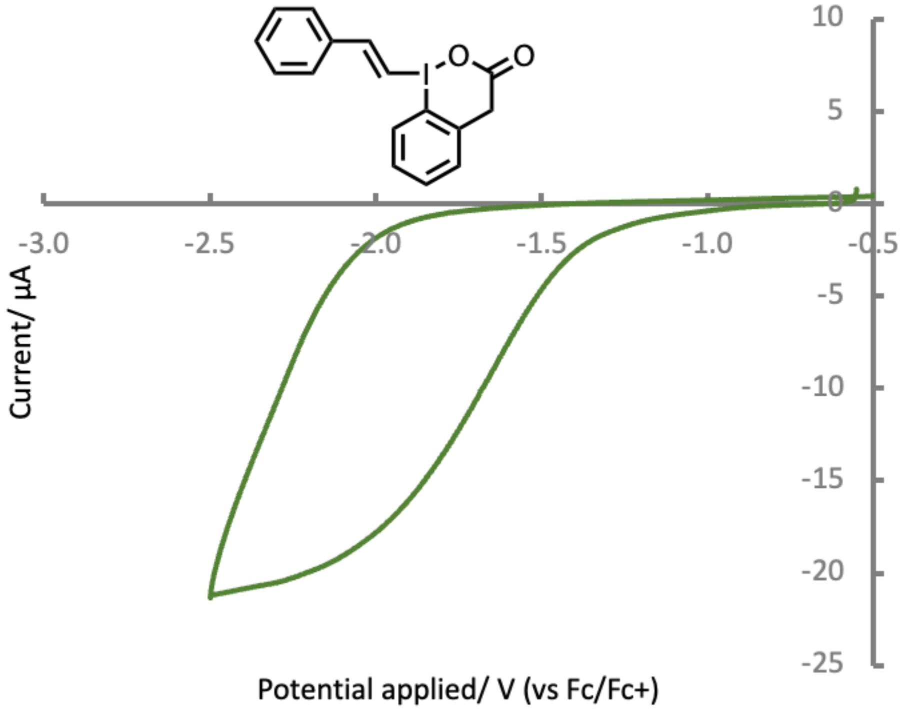

Figure S34. CV of **7.**

8.3 Measured reduction potentials

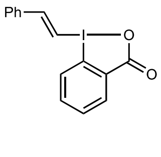

1a
-1.47 V

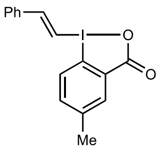

1b
-1.57 V

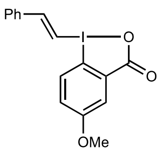

1b
-1.59 V

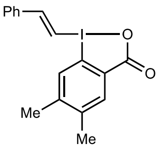

1c
-1.50 V

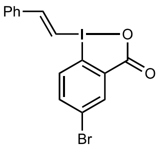

1d
-1.37 V

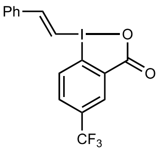

1e
-1.33 V

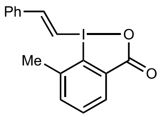

1f
-1.35 V

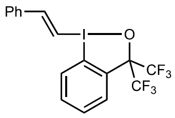

2a
-1.96 V

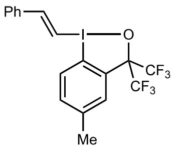

2b
-1.97 V

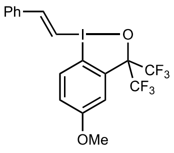

2c
-2.00 V

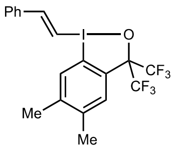

2d
-1.98 V

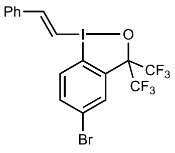

2e
-1.81V

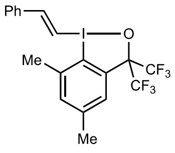

2f
-1.68 V

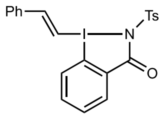

3a
-1.39 V

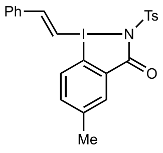

3b
-1.48 V

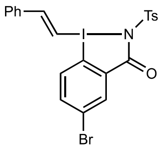

3c
-1.32 V

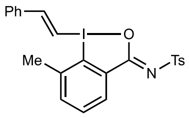

4
-1.21 V

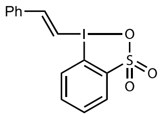

5a
-1.21 V

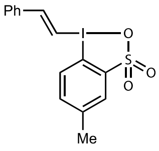

5b
-1.17V

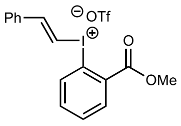

6a
-0.89 V

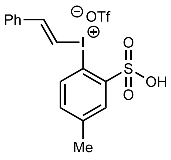

6b
-1.16 V

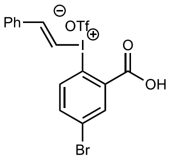

6c
-0.75 V

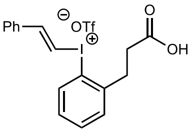

6d
-0.98 V

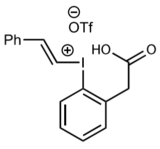

6e
-0.92 V)

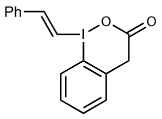

7
-1.61 V

9 NMR of substrates

9.1.1 (2-Amino-4,5-dimethylphenyl)-1,1,1,3,3,3-hexafluoropropan-2-ol (9d)

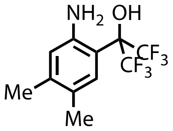

δH (400 MHz, CDCl3)

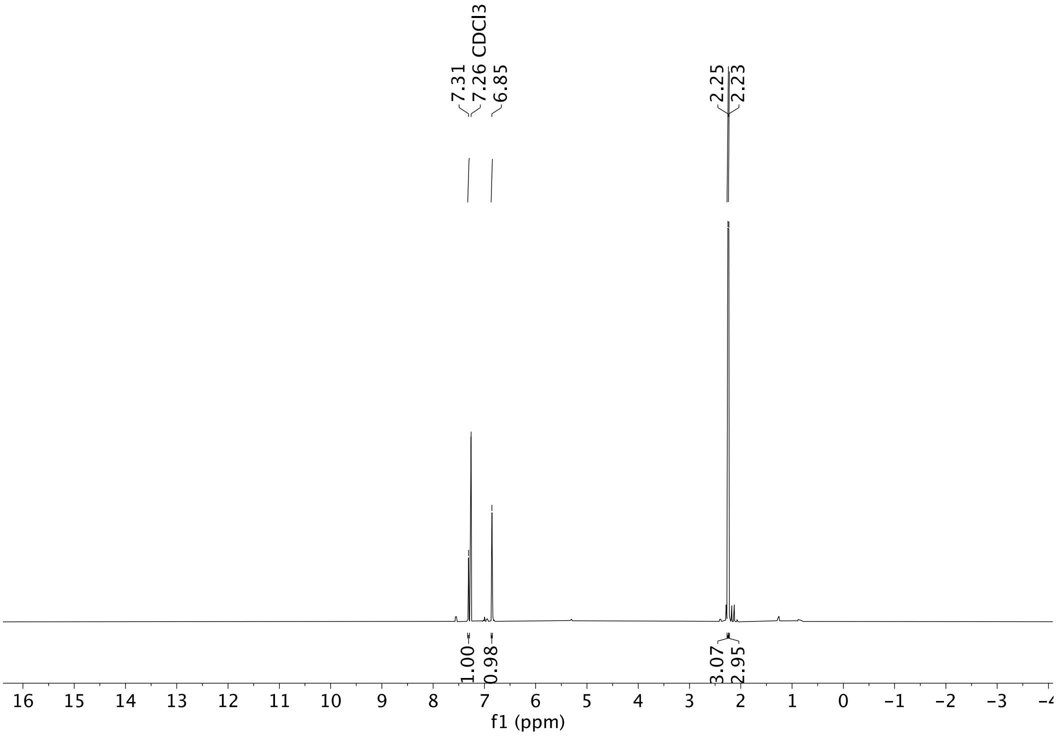

δF (377 MHz, CDCl3)

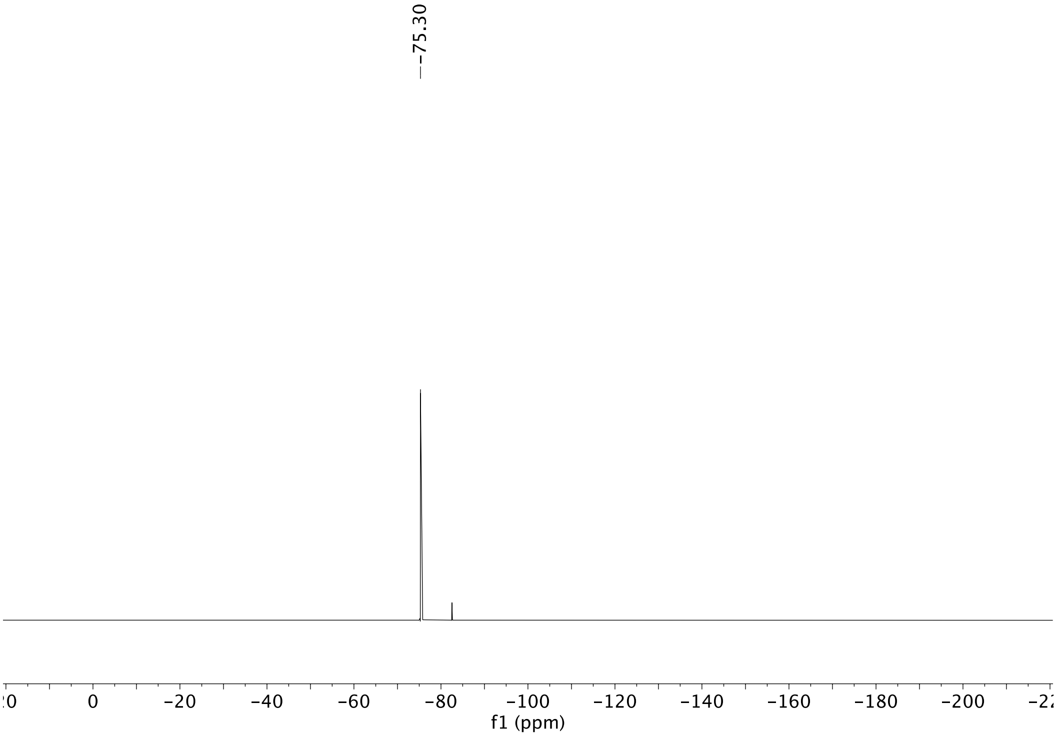

δ$_C$ (101 MHz, $CDCl_3$)

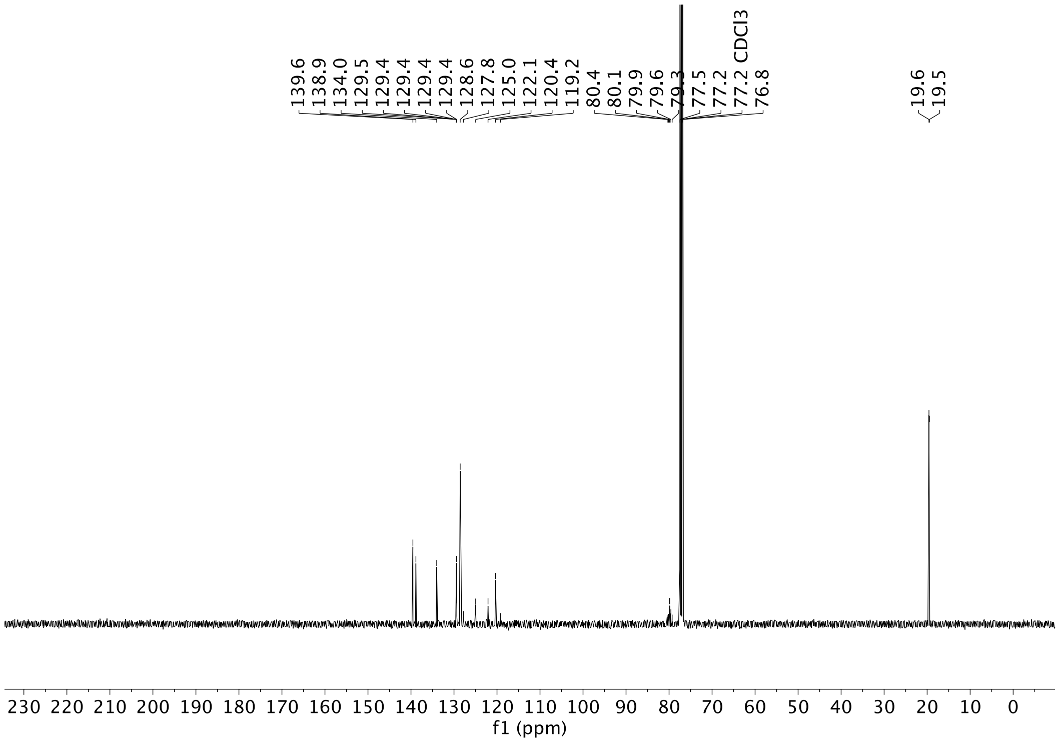

9.1.2 2-(2-Amino-5-bromophenyl)-1,1,1,3,3,3-hexafluoropropan-2-ol (9e)

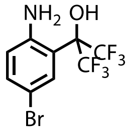

δH (400 MHz, CDCl3)

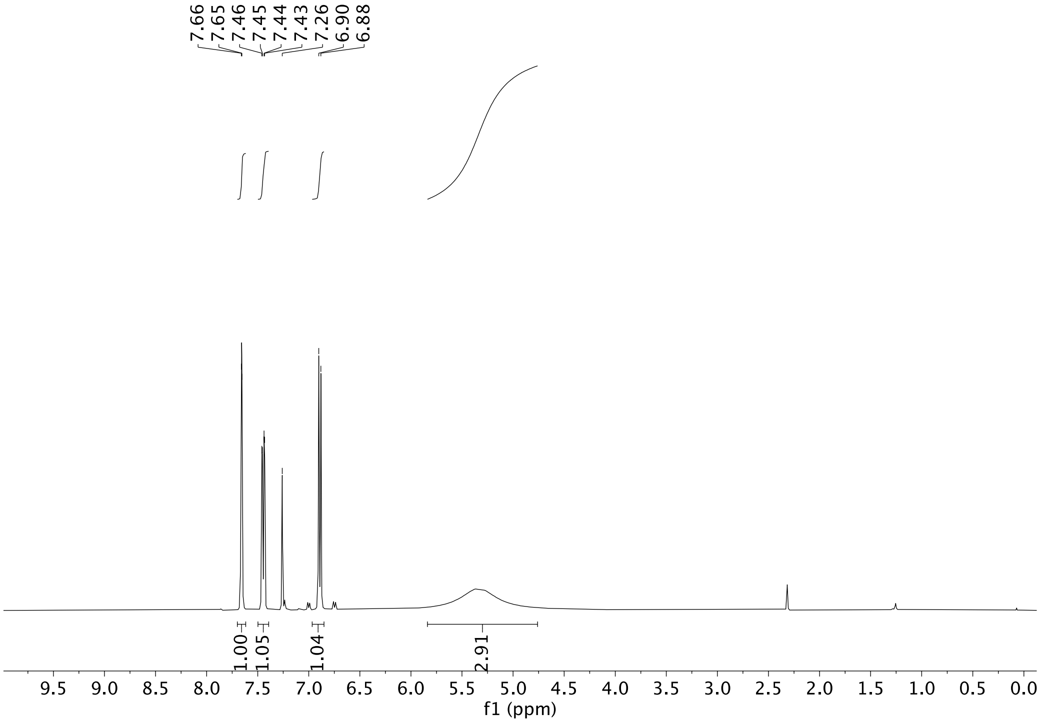

δF (377 MHz, CDCl3)

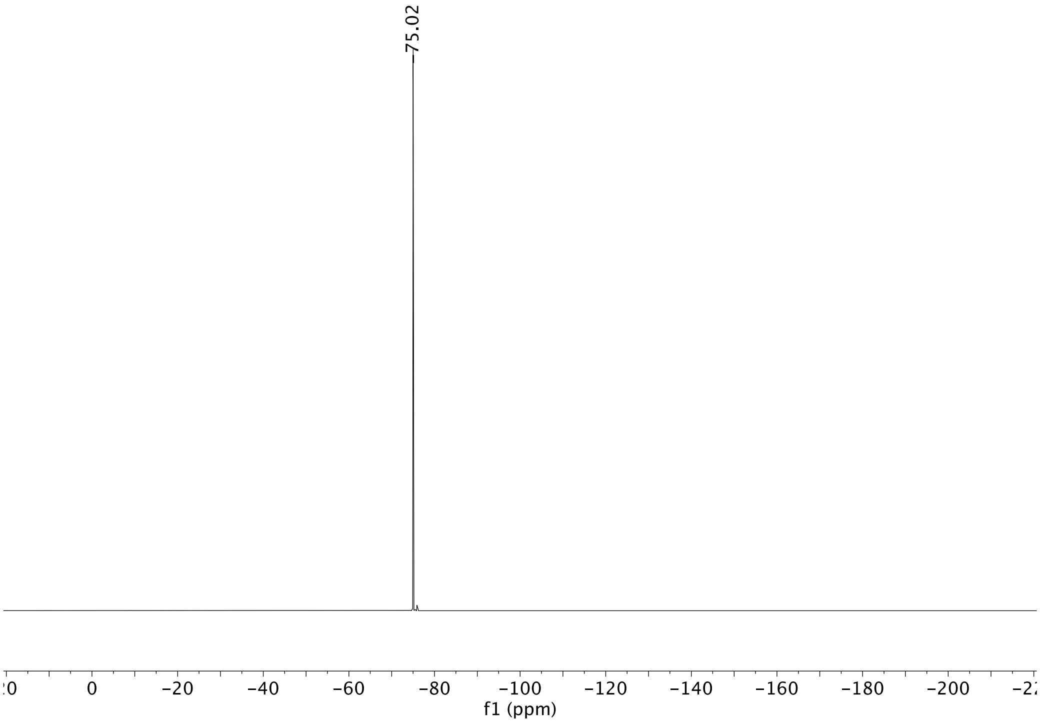

δC (101 MHz, CDCl3)

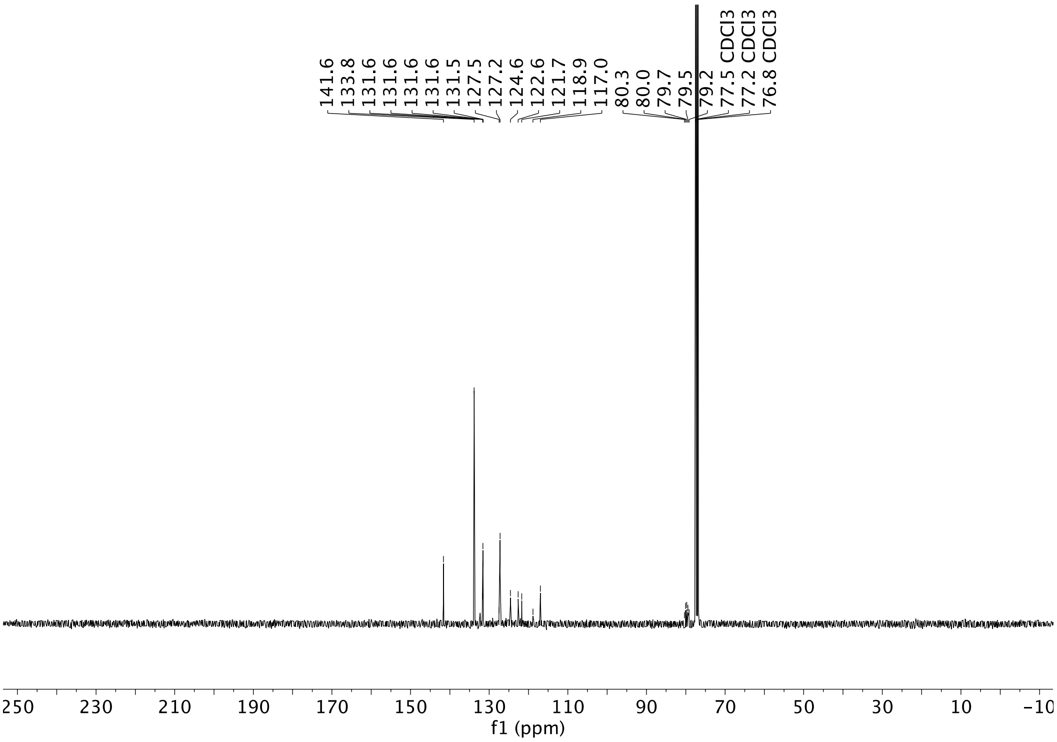

9.1.3 2-(2-Amino-3,5-dimethylphenyl)-1,1,1,3,3,3-hexafluoropropan-2-ol (9f)

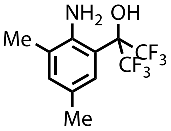

δH (400 MHz, CDCl3)

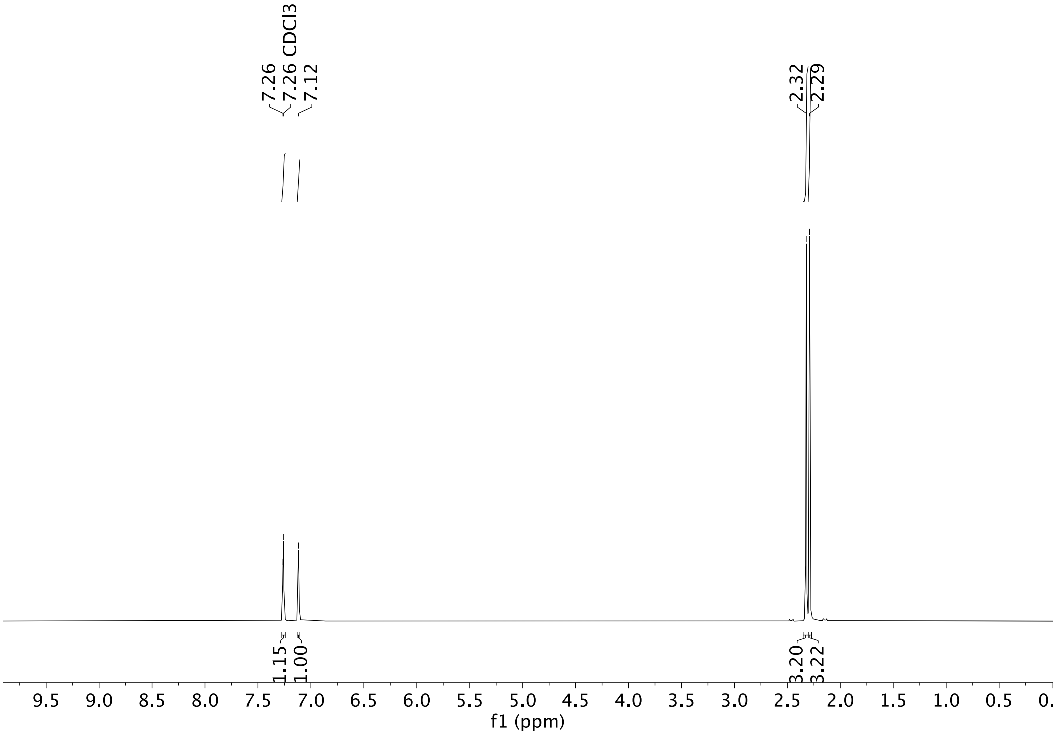

δF (377 MHz, CDCl3)

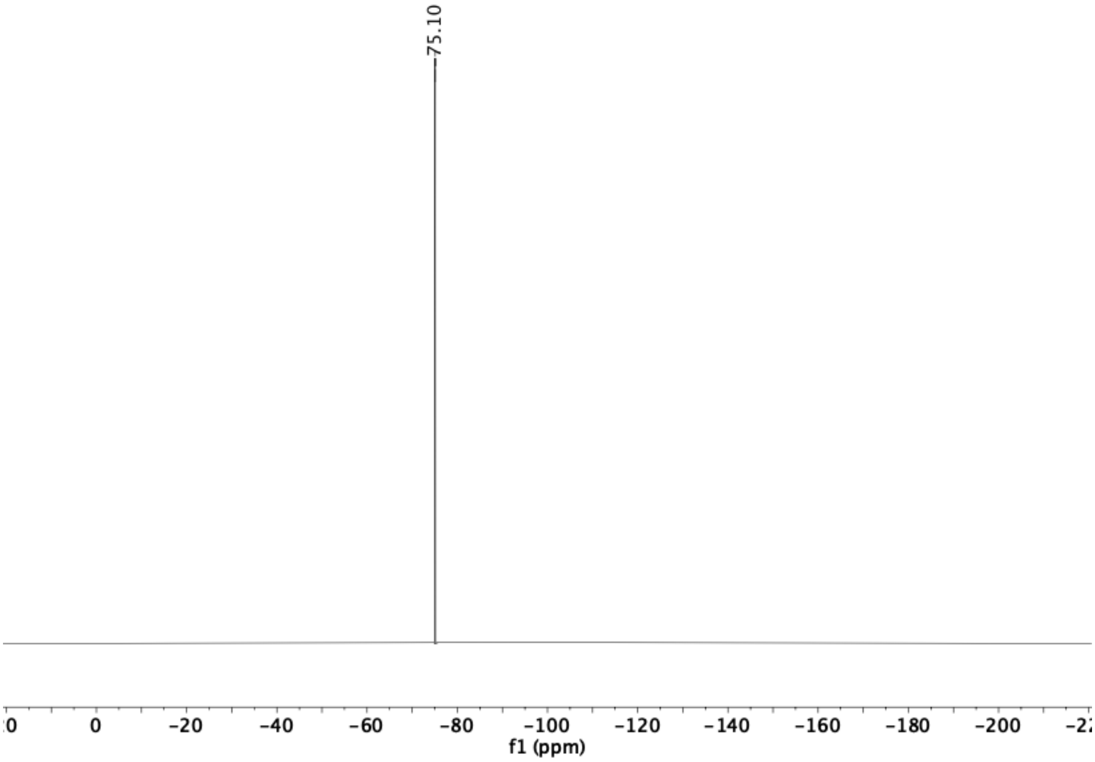

δC (101 MHz, CDCl3)

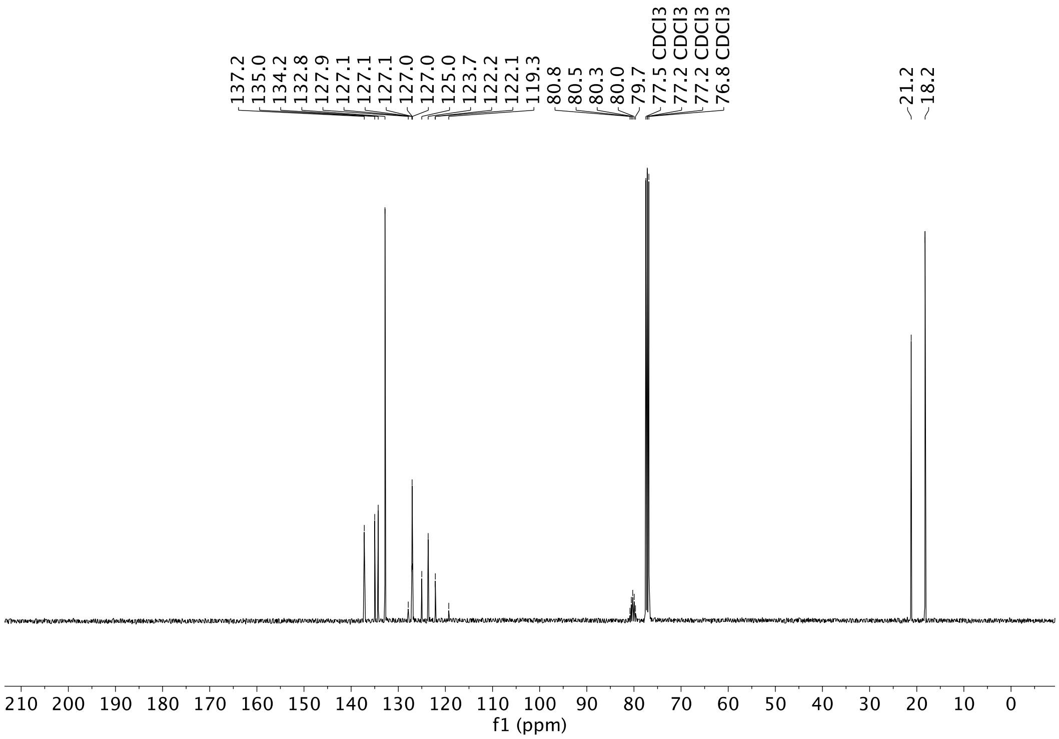

9.1.4 1,1,1,3,3,3-Hexafluoro-2-(2-iodo-4,5-dimethylphenyl)propan-2-ol (10d)

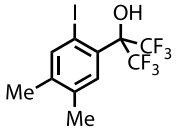

δH (400 MHz, CDCl3)

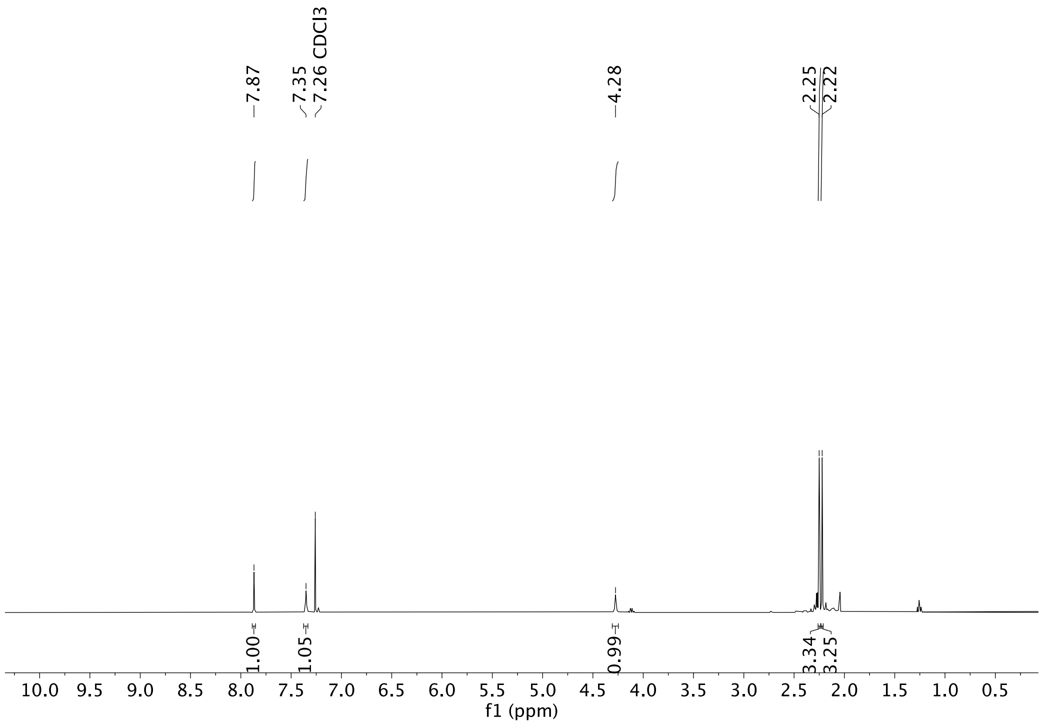

δF (377 MHz, CDCl3)

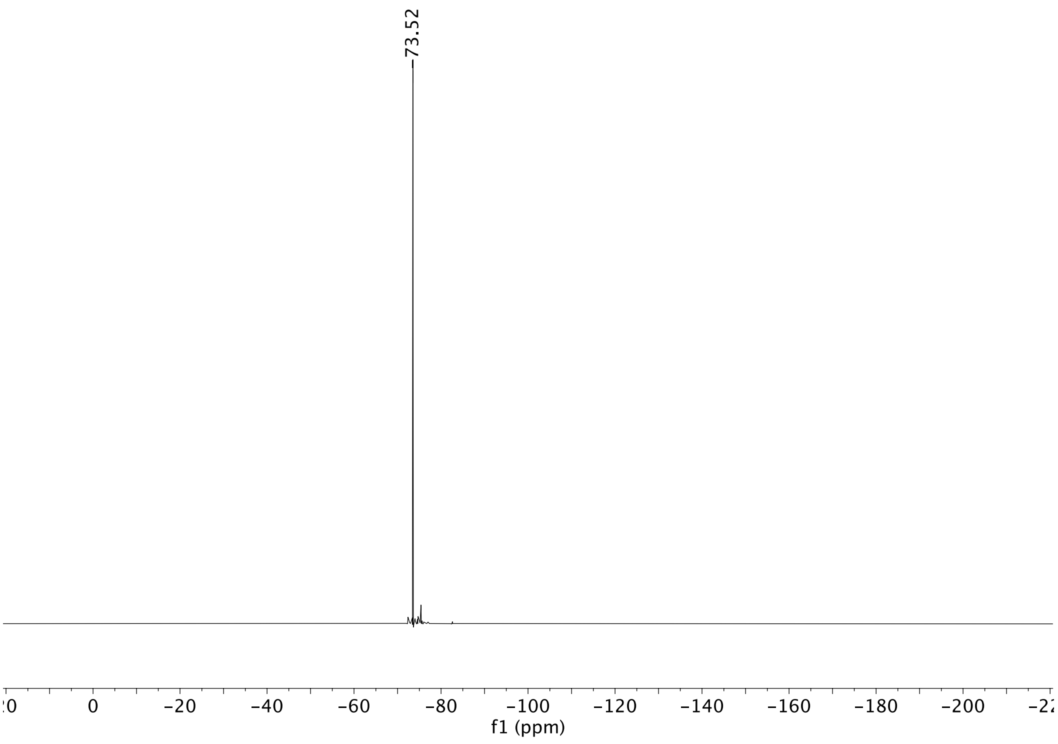

δC (101 MHz, CDCl3)

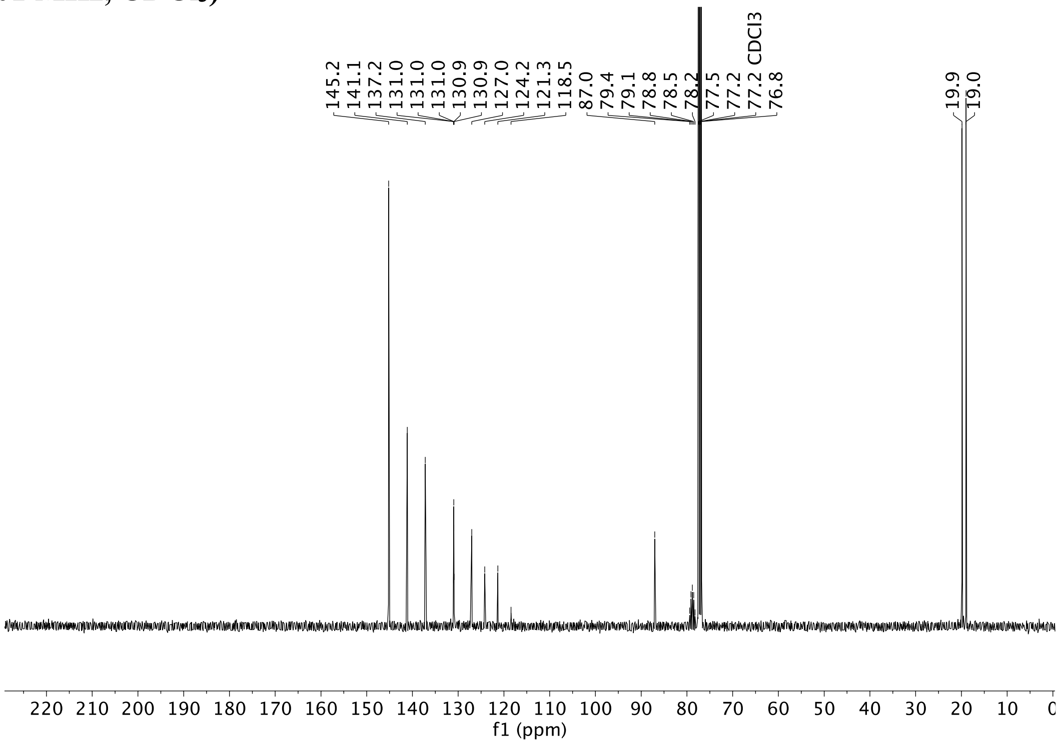

9.1.5 2-(5-Bromo-2-iodophenyl)-1,1,1,3,3,3-hexafluoropropan-2-ol (10e)

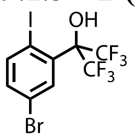

δH (400 MHz, CDCl3)

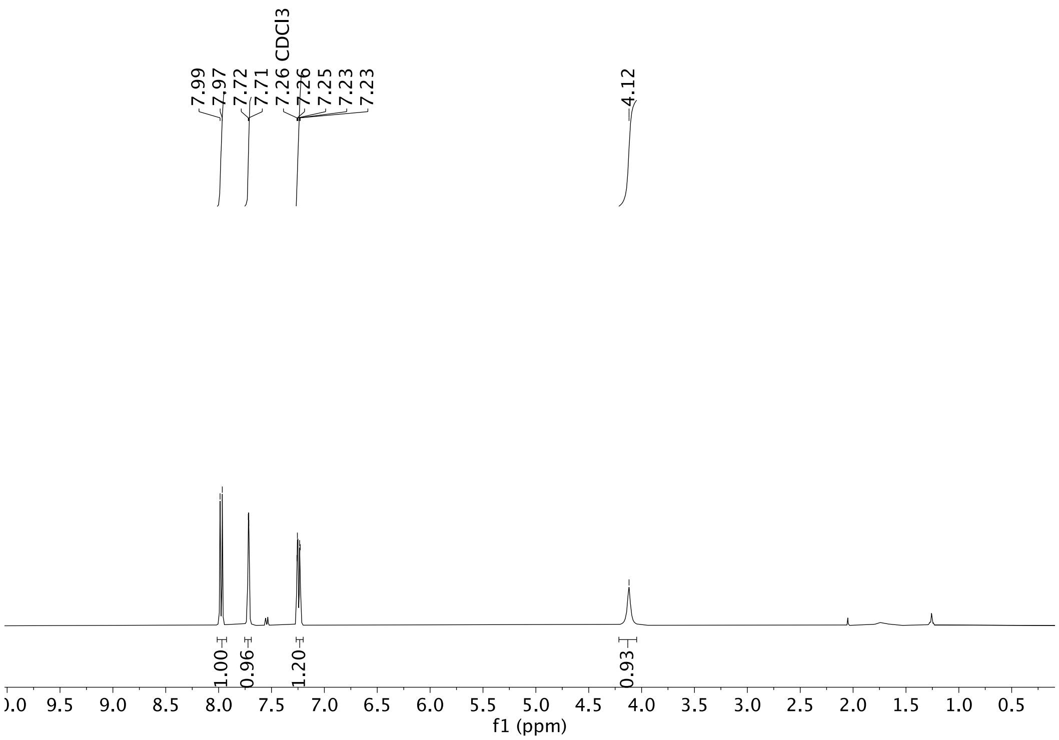

δF (377 MHz, CDCl3)

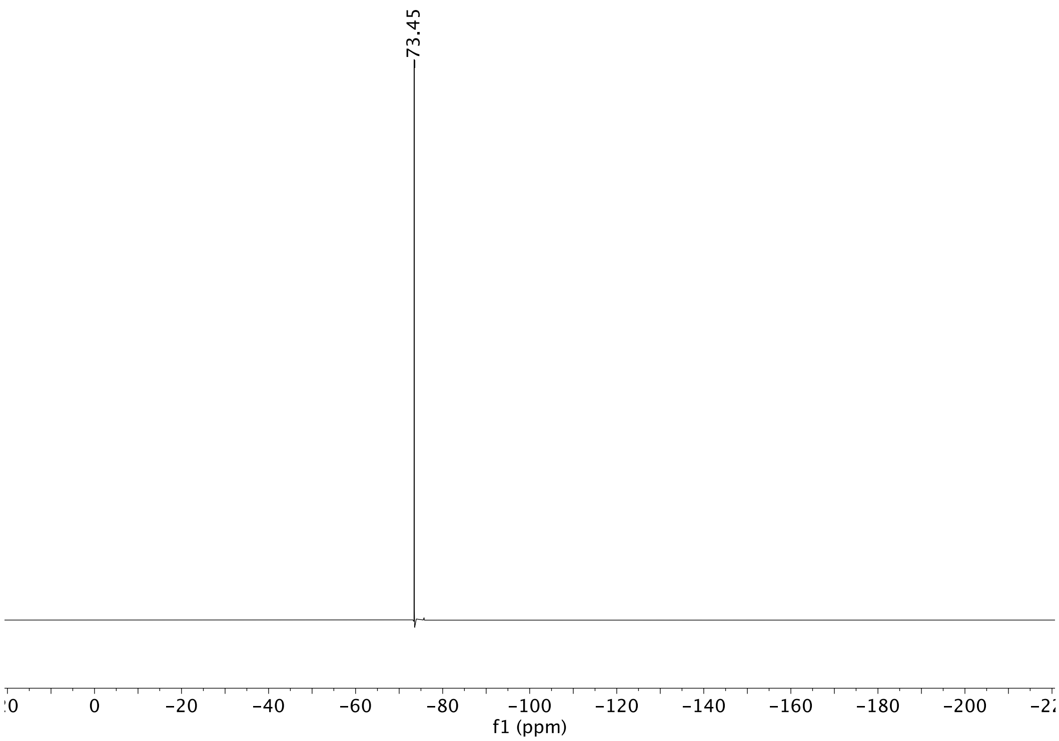

δ$_C$ (101 MHz, CDCl$_3$)

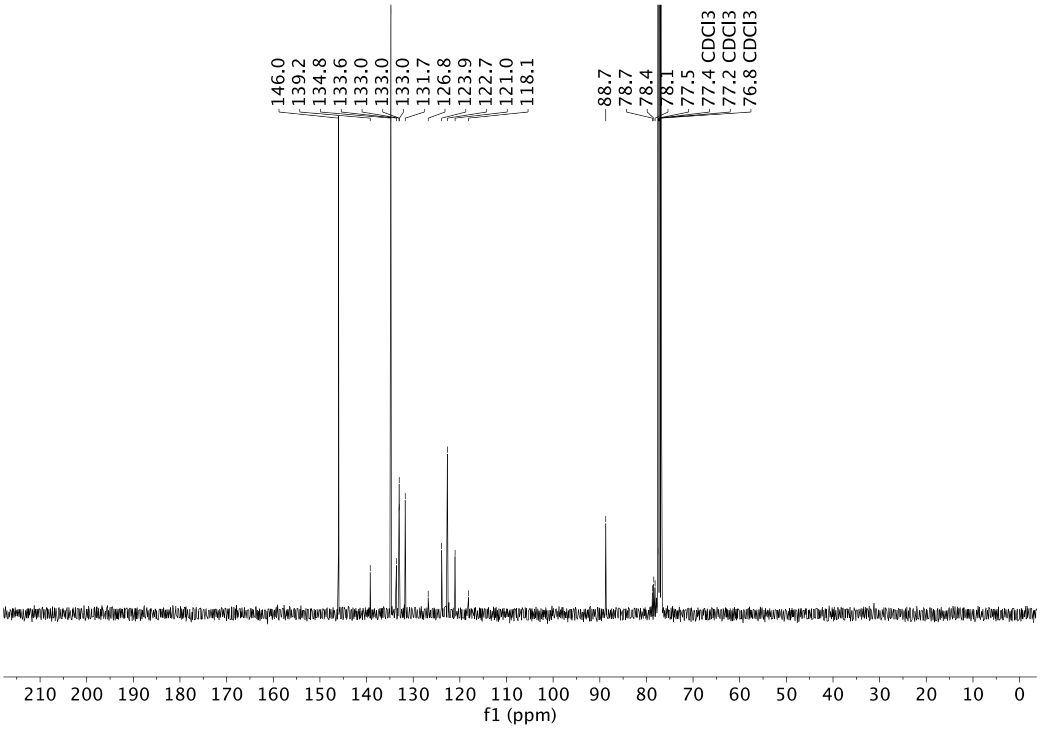

9.1.6 2-(3,5-Dimethyl-2-iodophenyl)-1,1,1,3,3,3-hexafluoropropan-2-ol (10f)

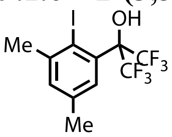

δ_H (400 MHz, $CDCl_3$)

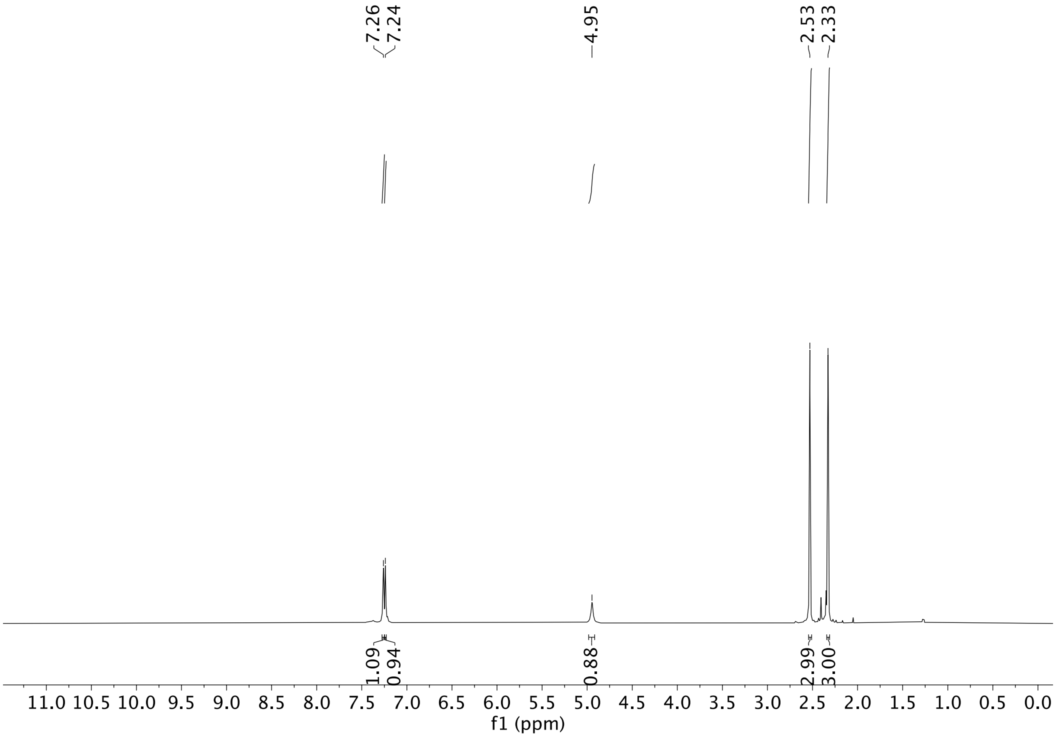

δ_F (377 MHz, $CDCl_3$)

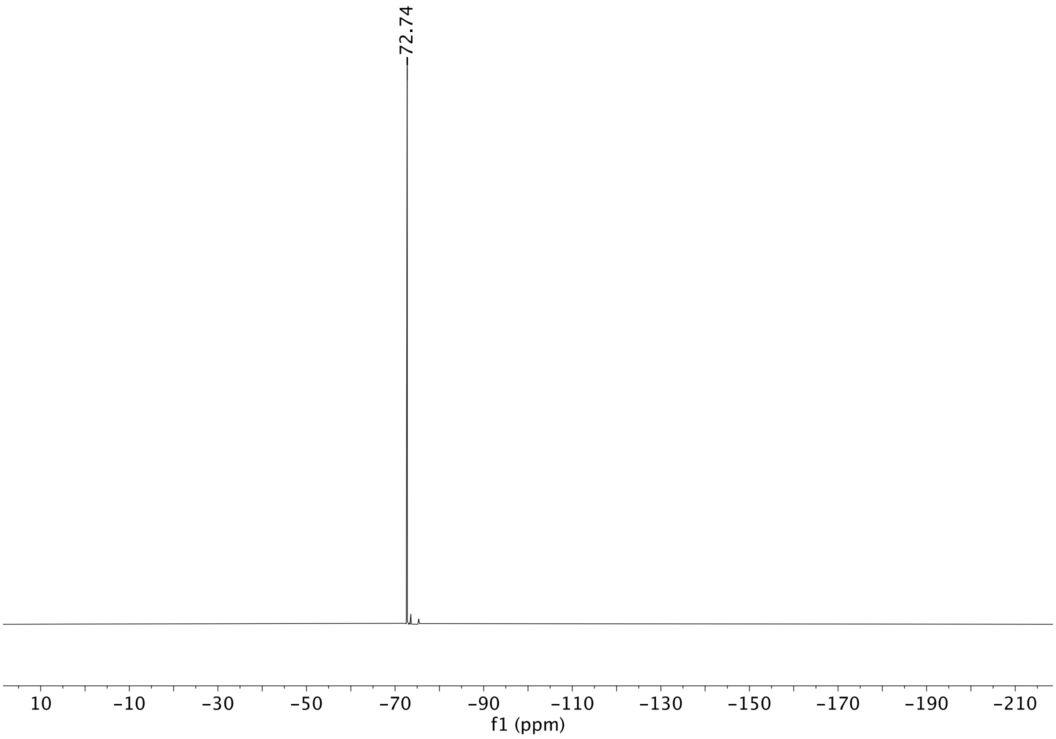

δC (101 MHz, CDCl3)

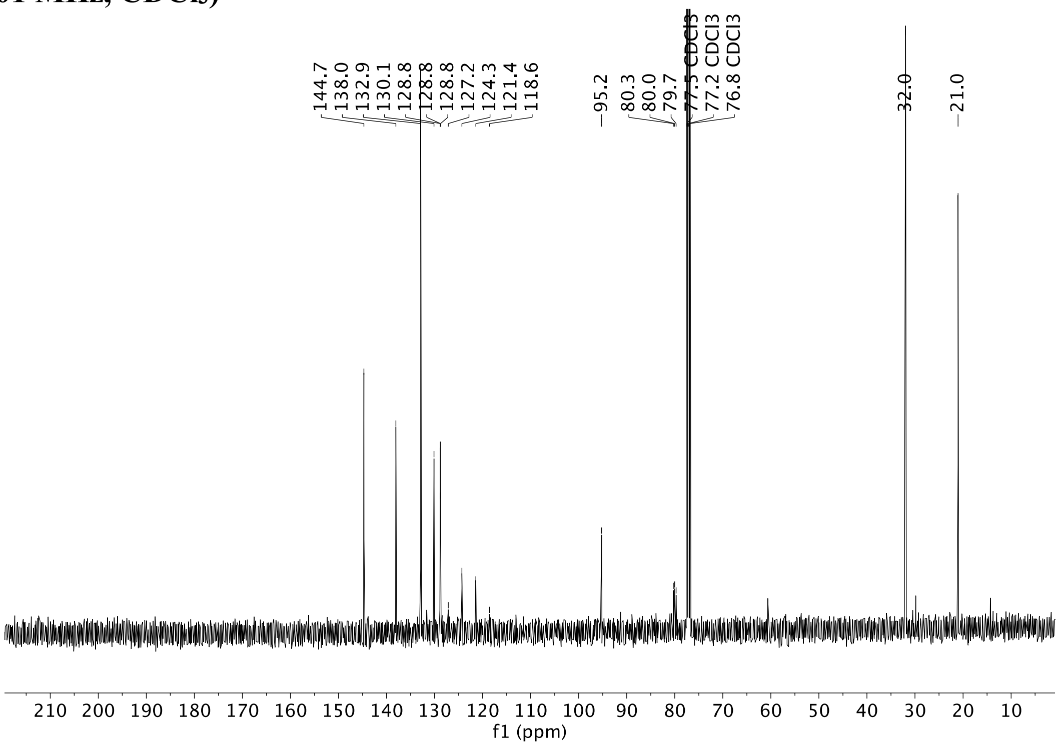

9.1.7 5-Methoxy-3,3-bis(trifluoromethyl)-1λ^3-benzo[*d*][1,2]iodaoxol-1(3H)-ol (11c)

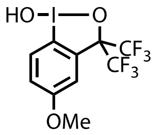

δH (400 MHz, CDCl3)

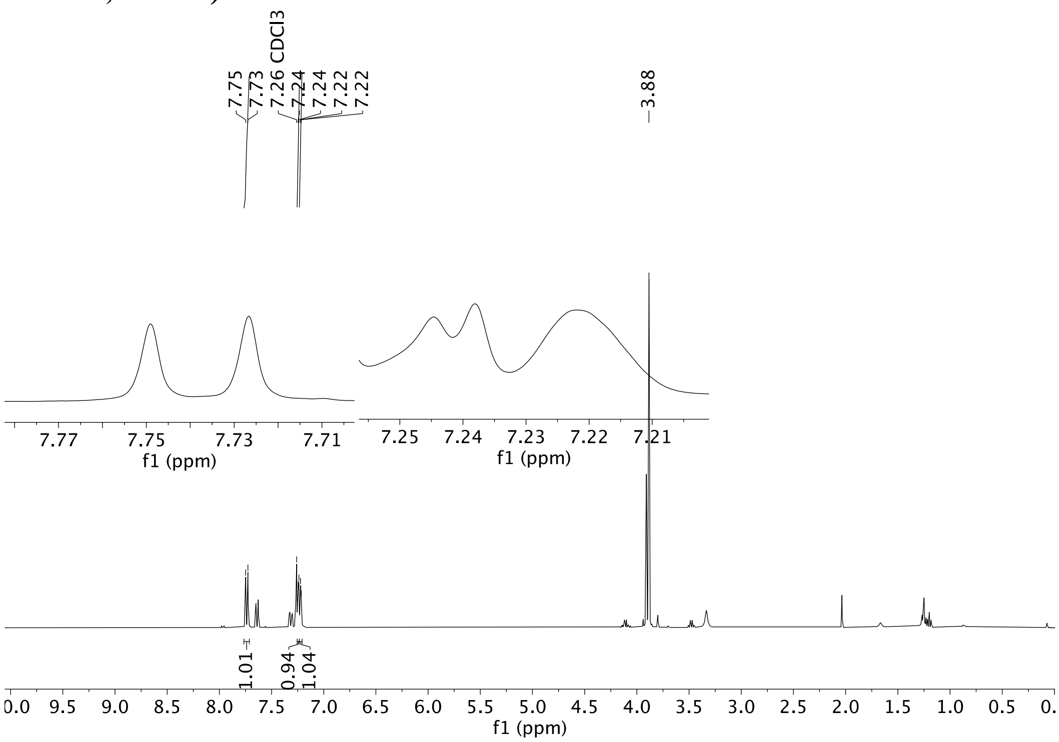

δF (377 MHz, CDCl3)

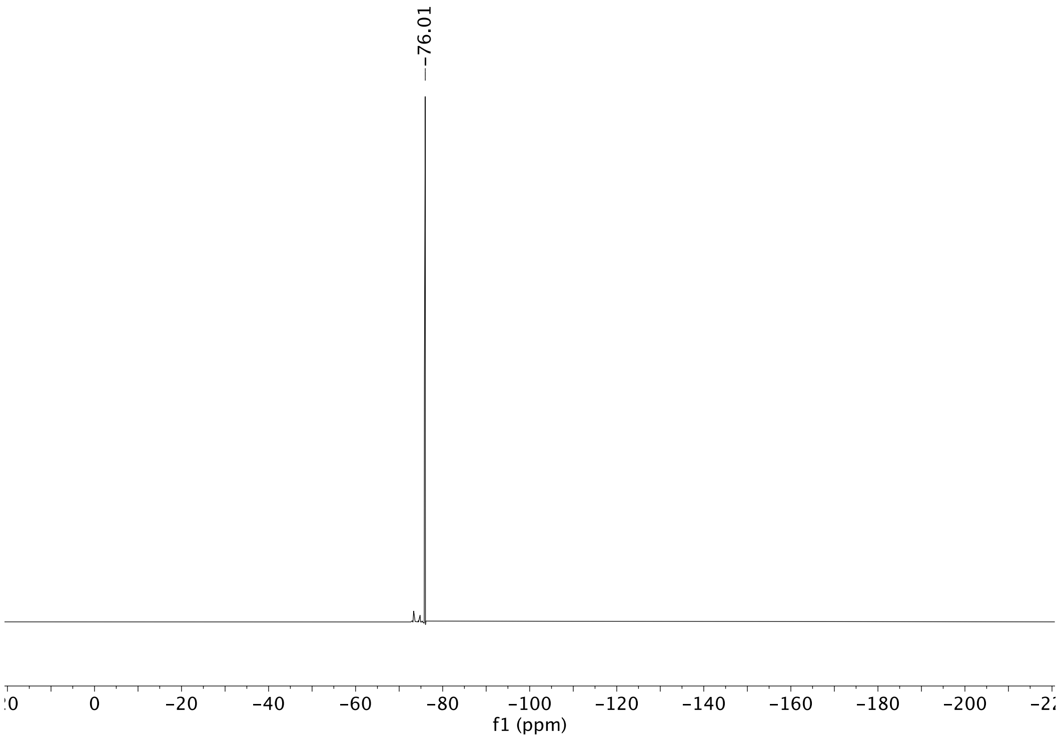

δ_C (101 MHz, $CDCl_3$)

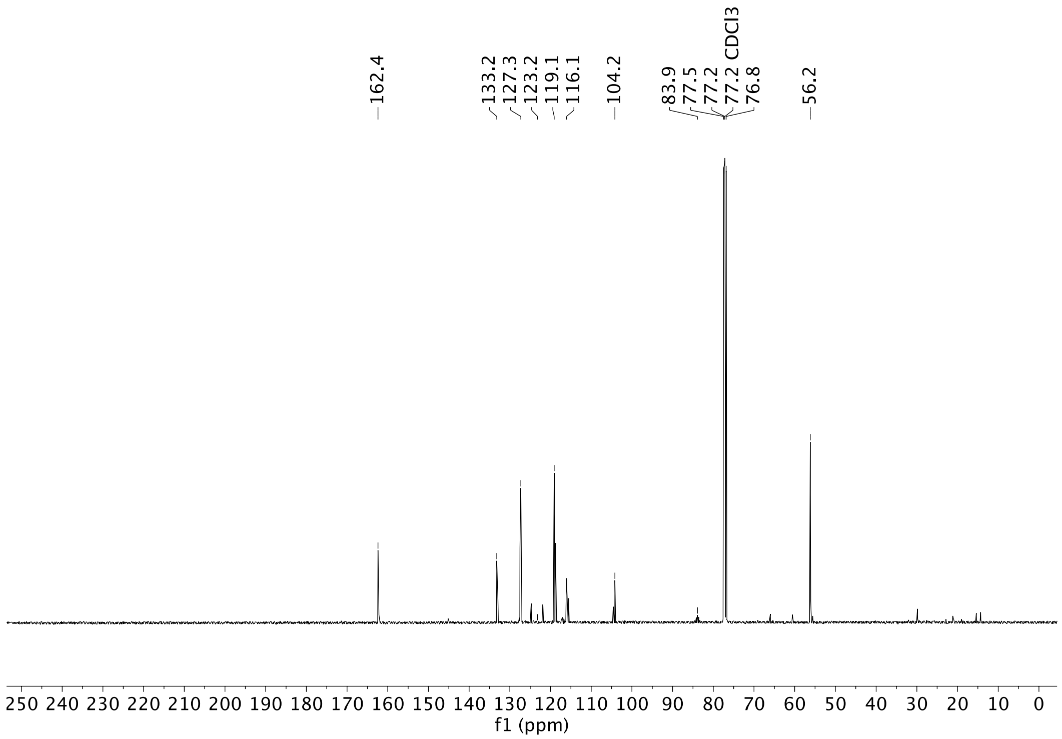

9.1.8 5,6-Dimethyl-3,3-bis(trifluoromethyl)-1λ^3-benzo[*d*][1,2]iodaoxol-1(3H)-ol (11d)

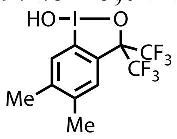

δ$_H$ (400 MHz, CDCl$_3$)

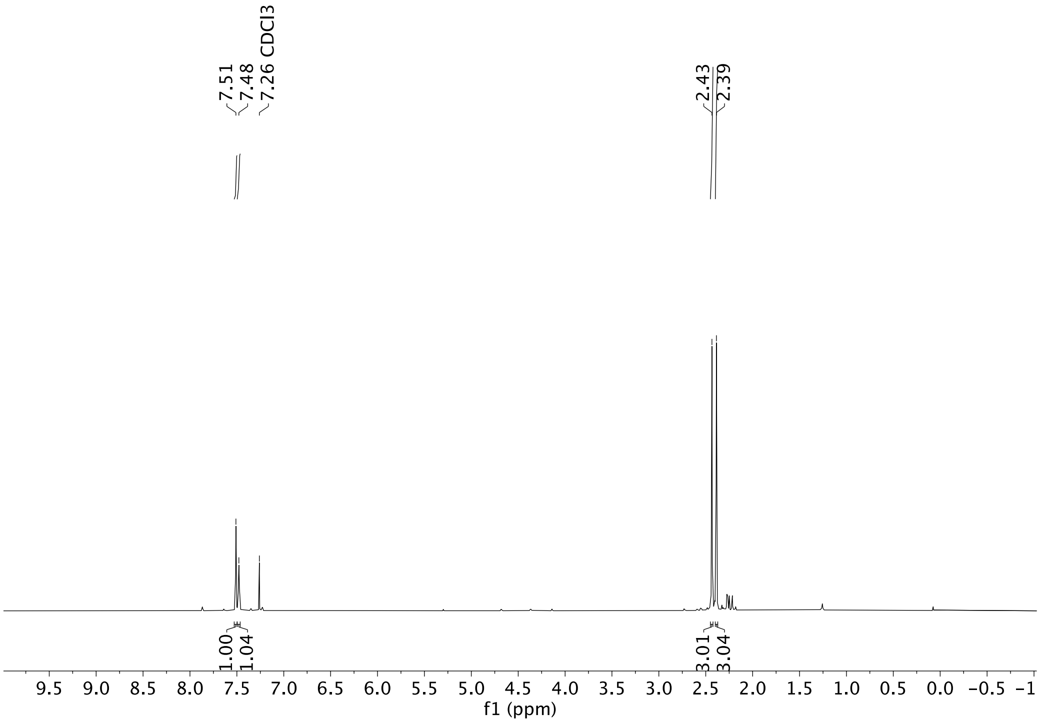

δ$_F$ (377 MHz, CDCl$_3$)

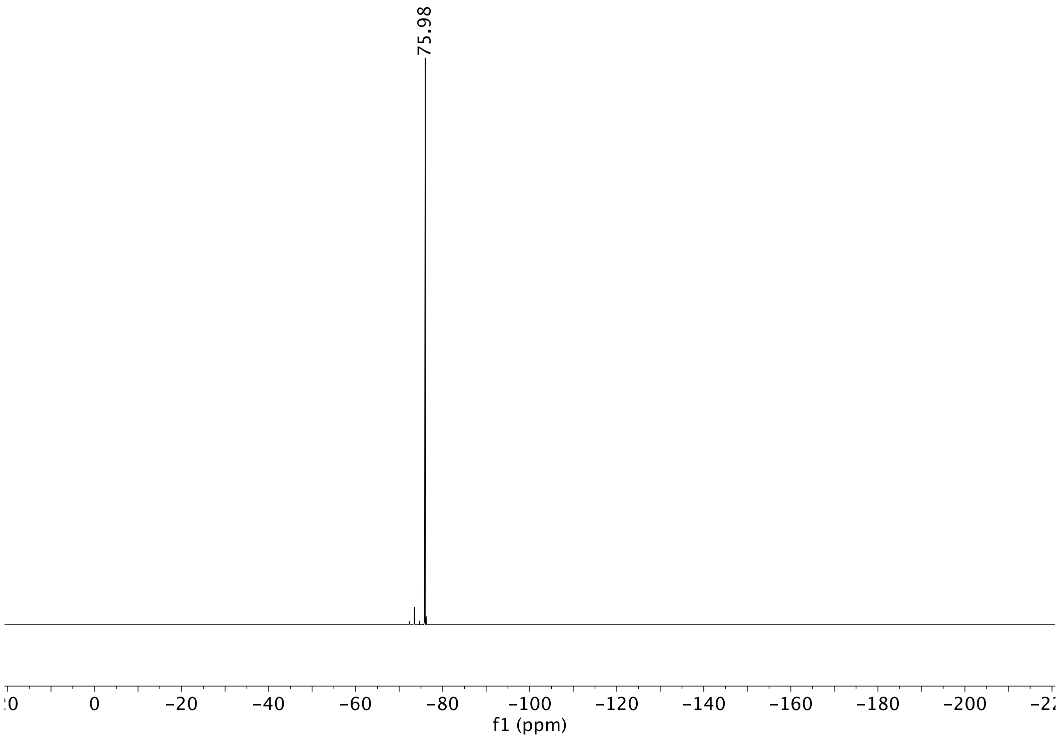

δC (101 MHz, CDCl3)

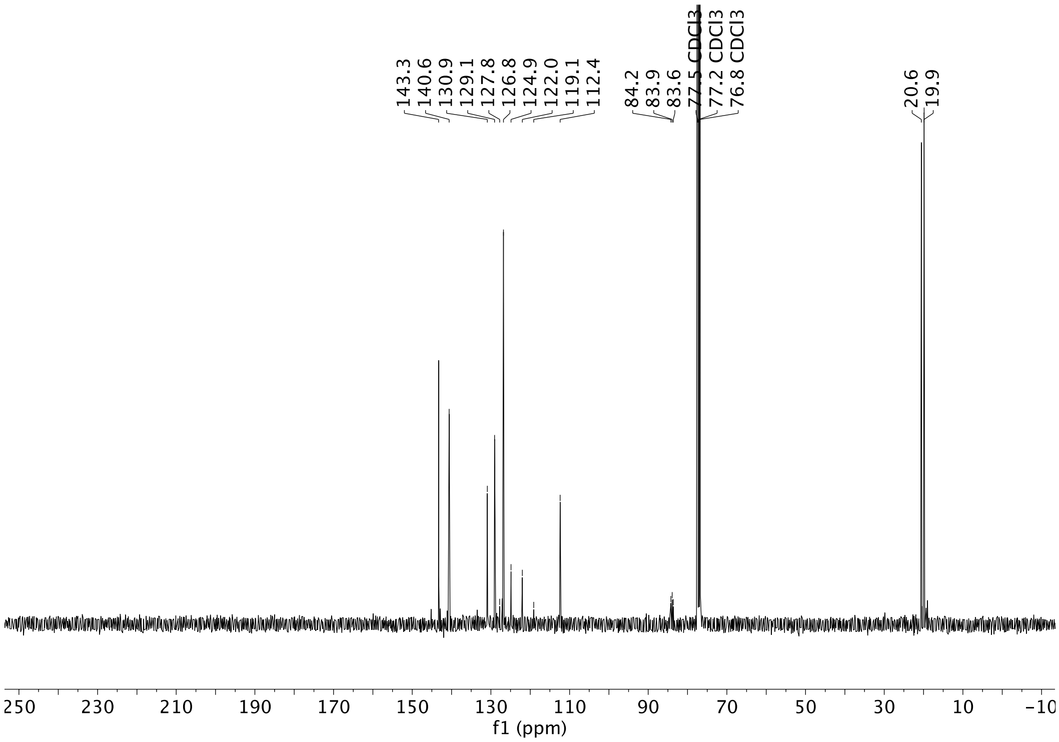

9.1.9 5-Bromo-3,3-bis(trifluoromethyl)-1λ^3-benzo[*d*][1,2]iodaoxol-1(3H)-ol (11e)

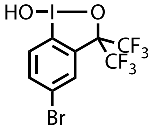

δH (400 MHz, CDCl3)

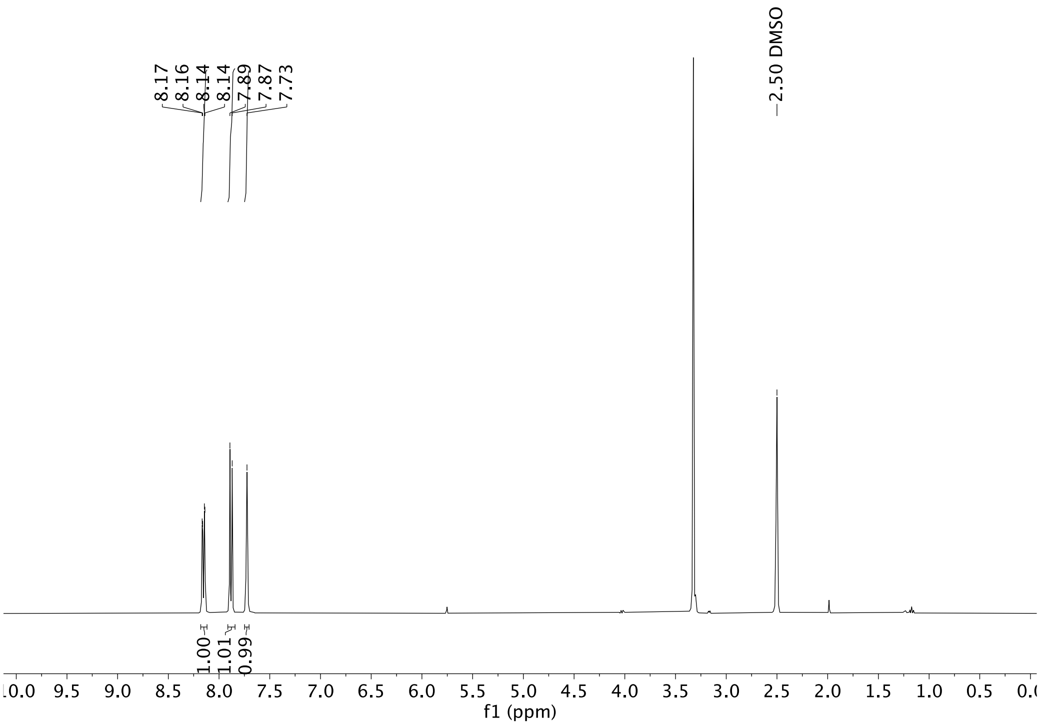

δF (377 MHz, CDCl3)

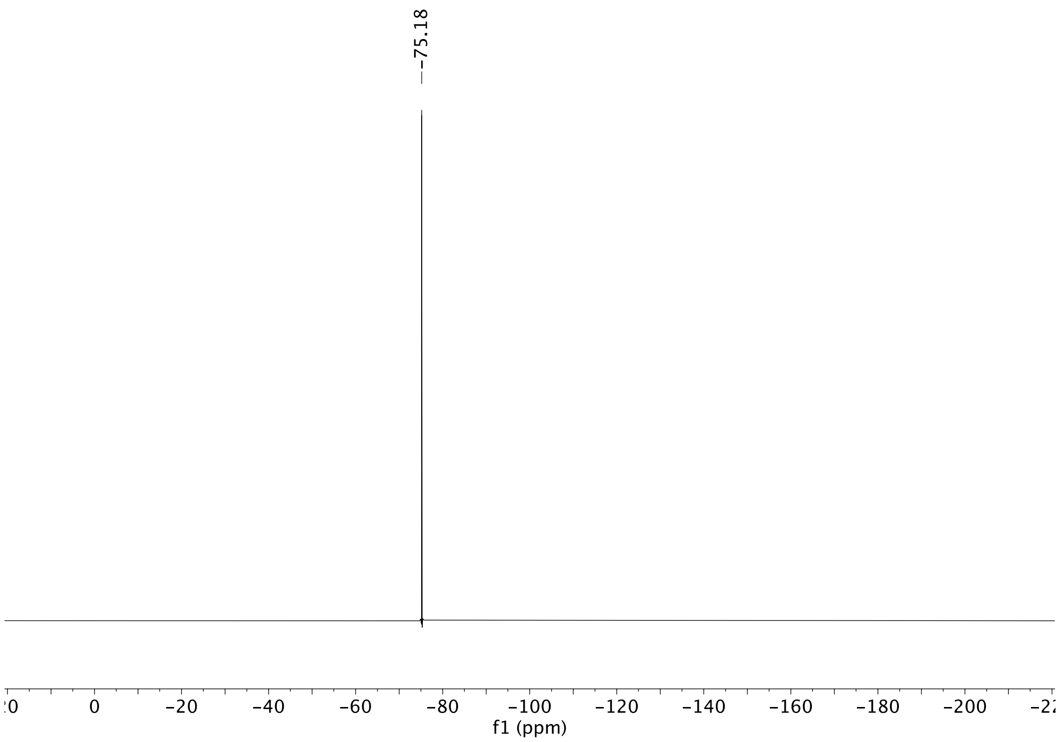

δC (101 MHz, CDCl3)

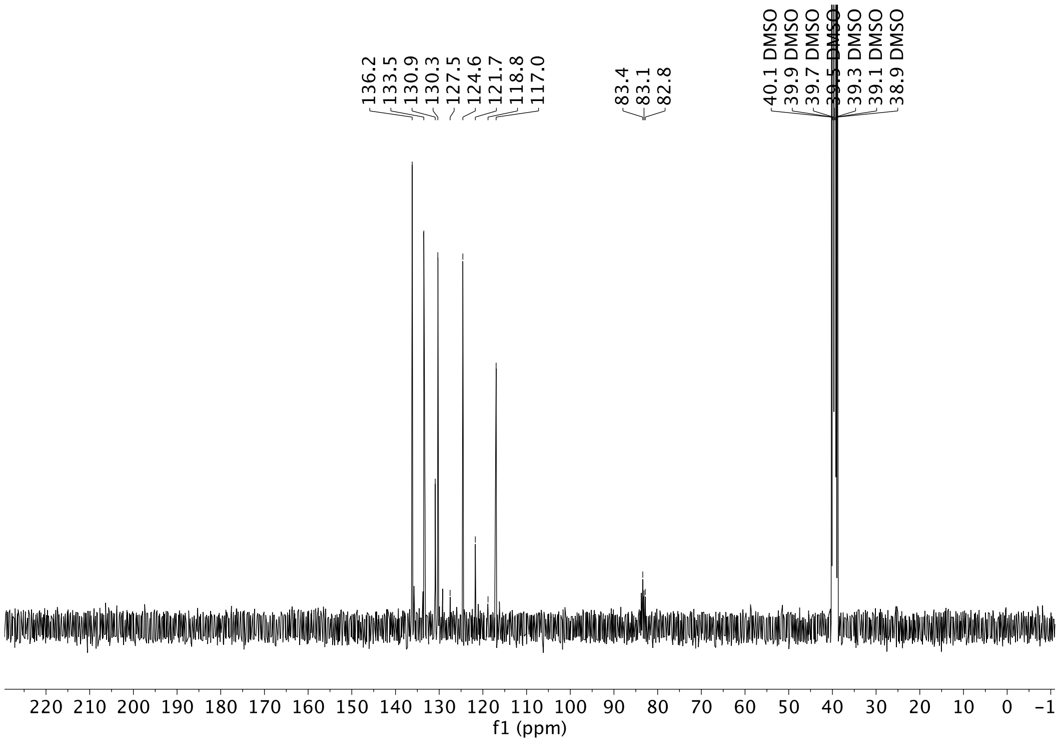

9.1.10 5,7-Dimethyl-3,3-bis(trifluoromethyl)-1λ^3-benzo[*d*][1,2]iodaoxol-1(3H)-ol (11f)

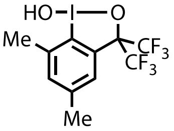

δ$_H$ (400 MHz, CDCl$_3$)

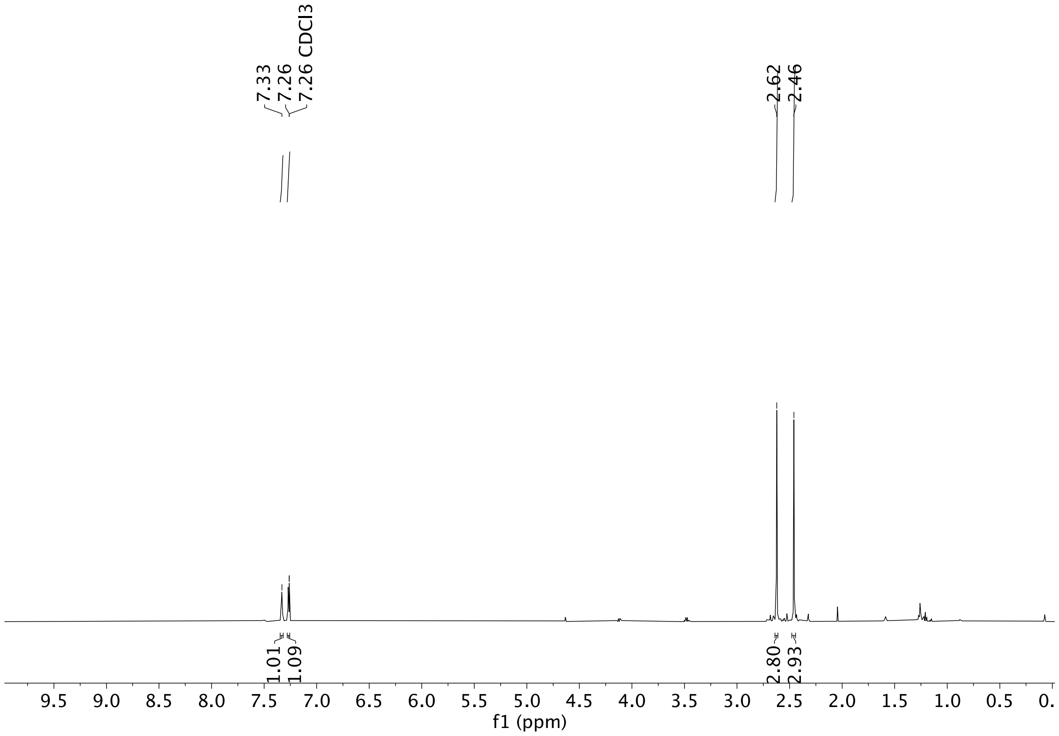

δ$_F$ (377 MHz, CDCl3)

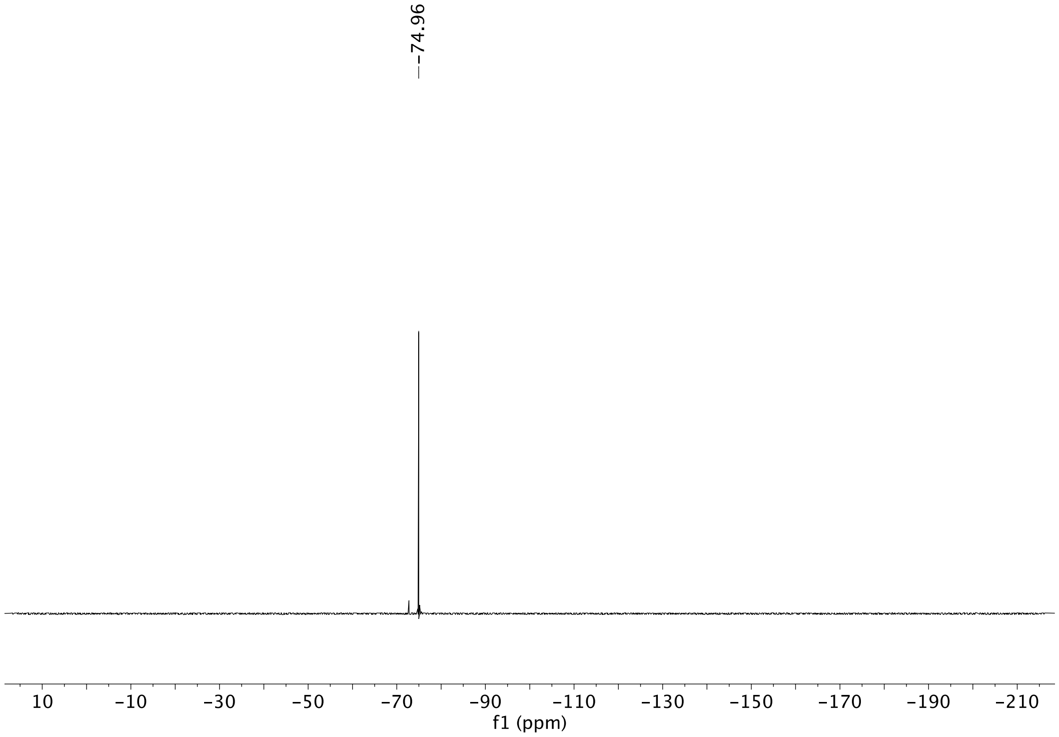

δC (101 MHz, CDCl3)

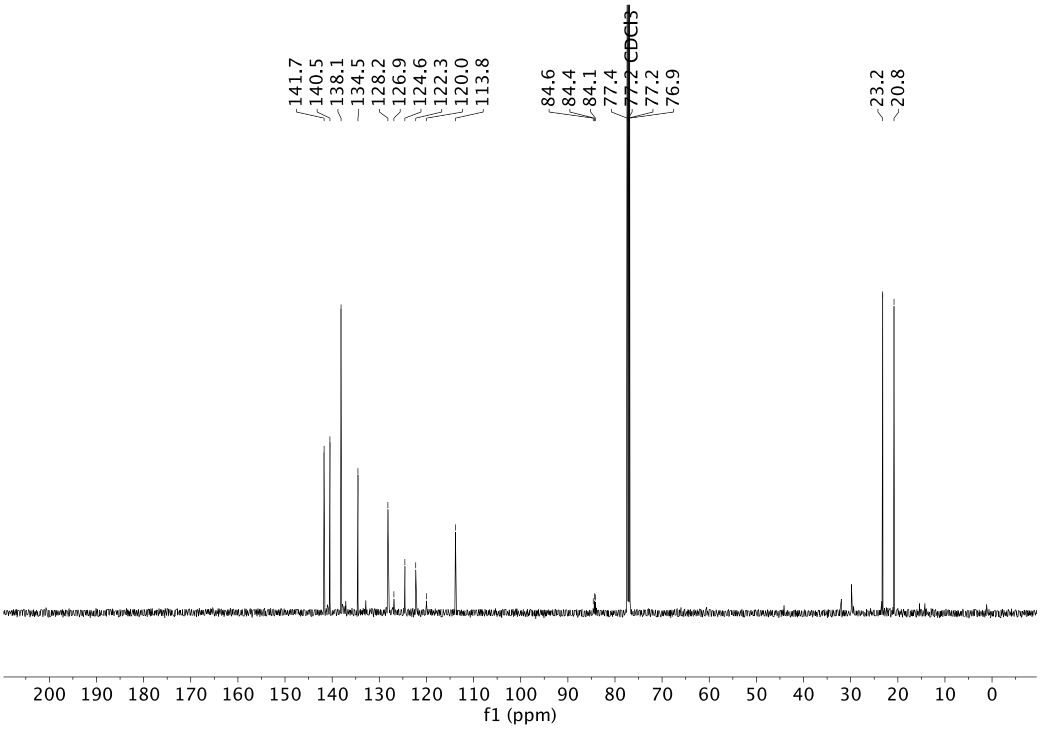

10 NMR of products

10.1.1 (*E*)-1-styryl-5-(trifluoromethyl)-1λ^3-benzo[*d*][1,2]iodaoxol-3(1H)-one (1f)

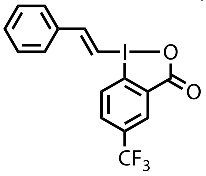

δ$_H$ (400 MHz, MeOD-d$_4$)

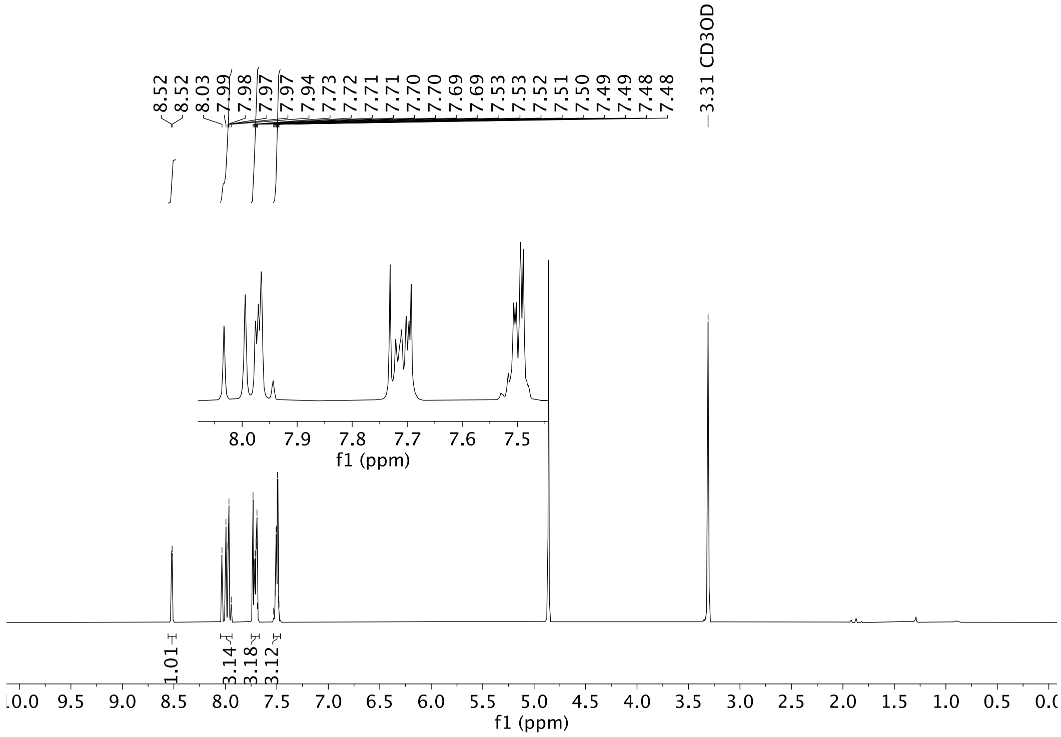

δ$_F$ (377 MHz, MeOD-d$_4$)

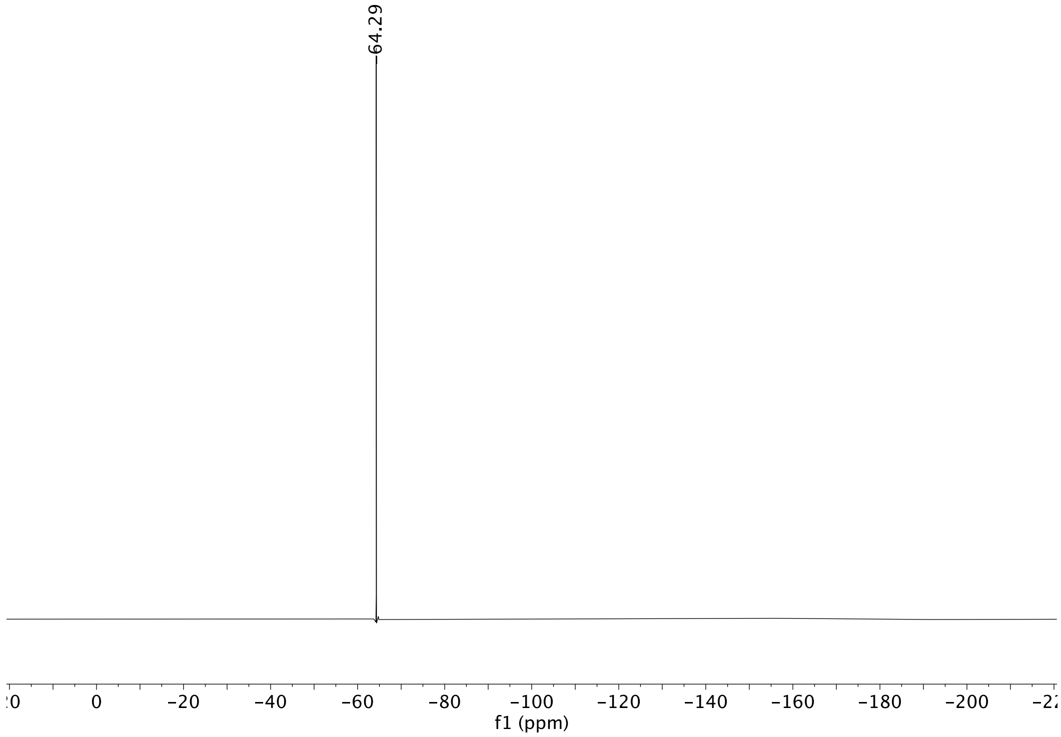

δ$_C$ (101 MHz, MeOD-d$_4$)

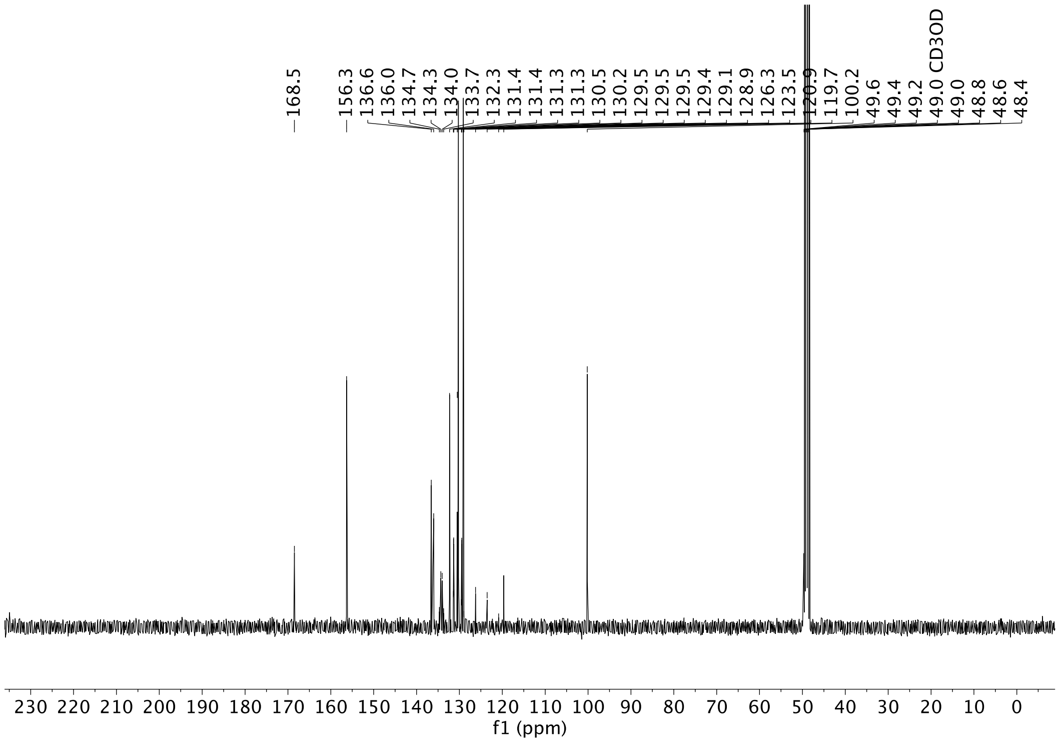

10.1.2 (*E*)-5-methyl-1-styryl-3,3-bis(trifluoromethyl)-1,3-dihydro-1λ^3-benzo[*d*][1,2]iodaoxole (2b)

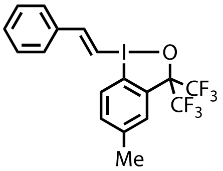

δ$_H$ (400 MHz, CDCl$_3$)

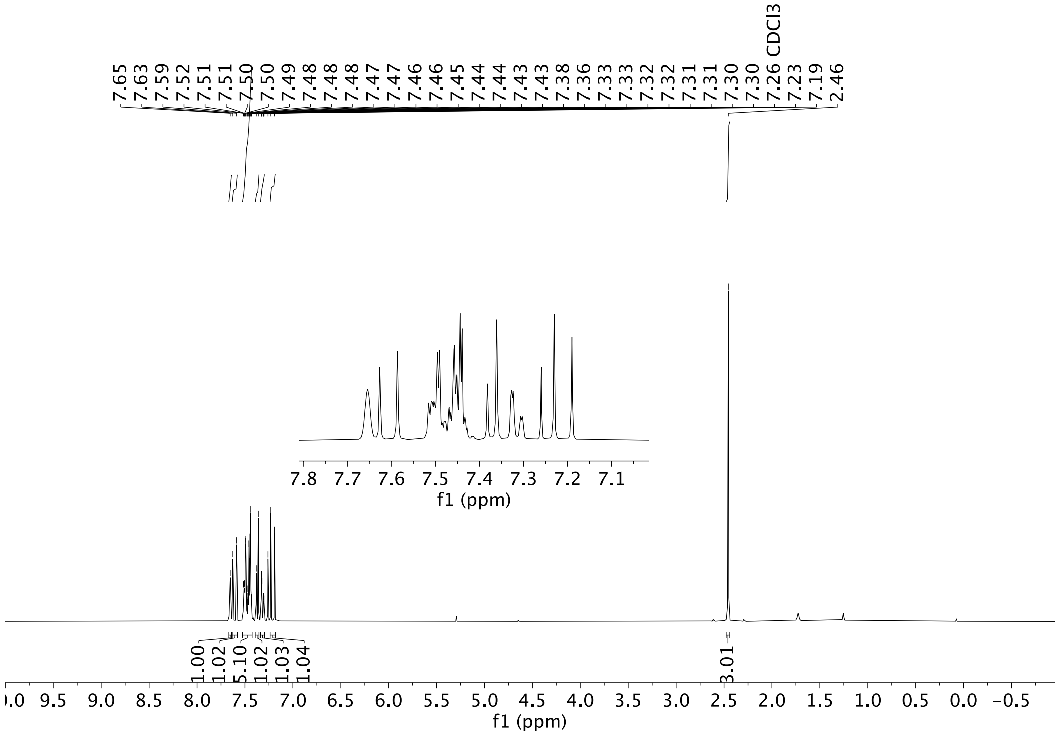

δ$_F$ (376 MHz, CDCl$_3$)

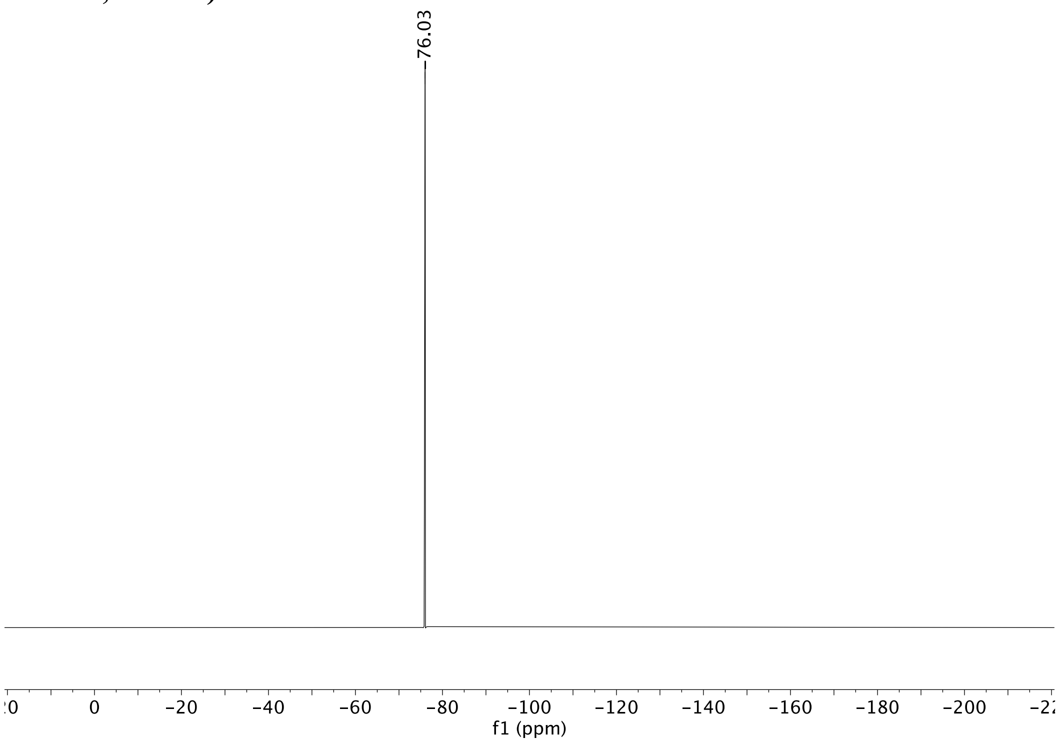

δC (101 MHz, CDCl₃)

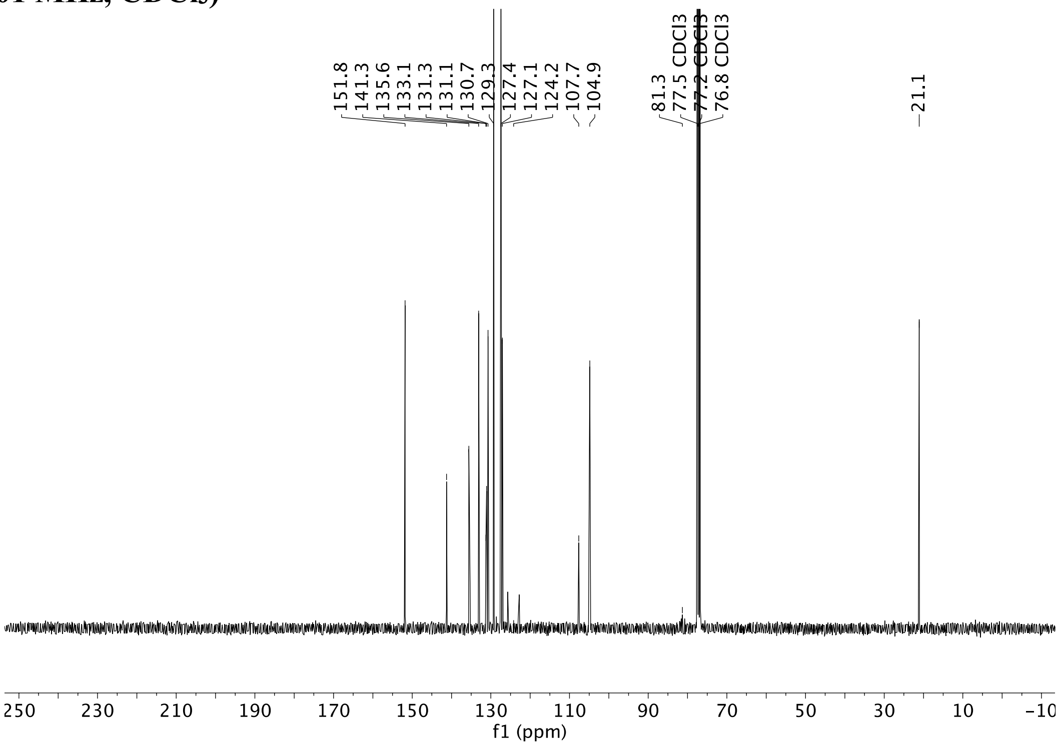

10.1.3 (*E*)-5-methoxy-1-styryl-3,3-bis(trifluoromethyl)-1,3-dihydro-1λ^3-benzo[*d*][1,2]iodaoxole (2c)

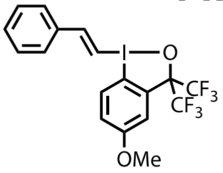

δ$_H$ (400 MHz, CDCl$_3$)

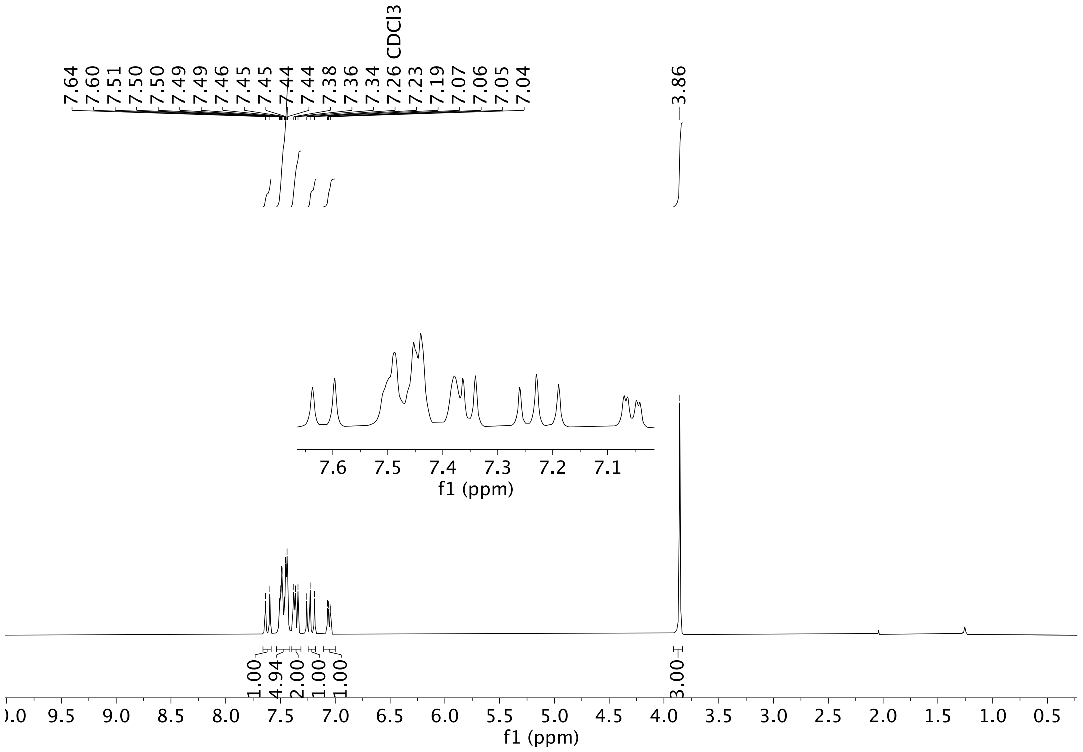

δ$_F$ (376 MHz, CDCl$_3$)

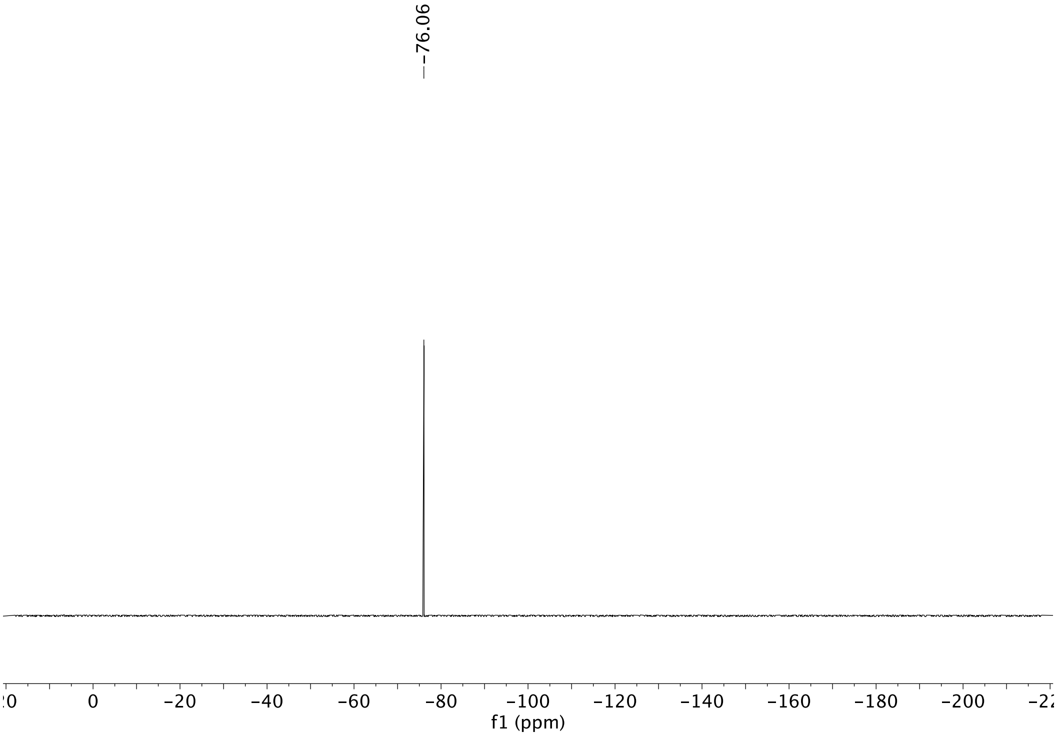

δC (101 MHz, CDCl3)

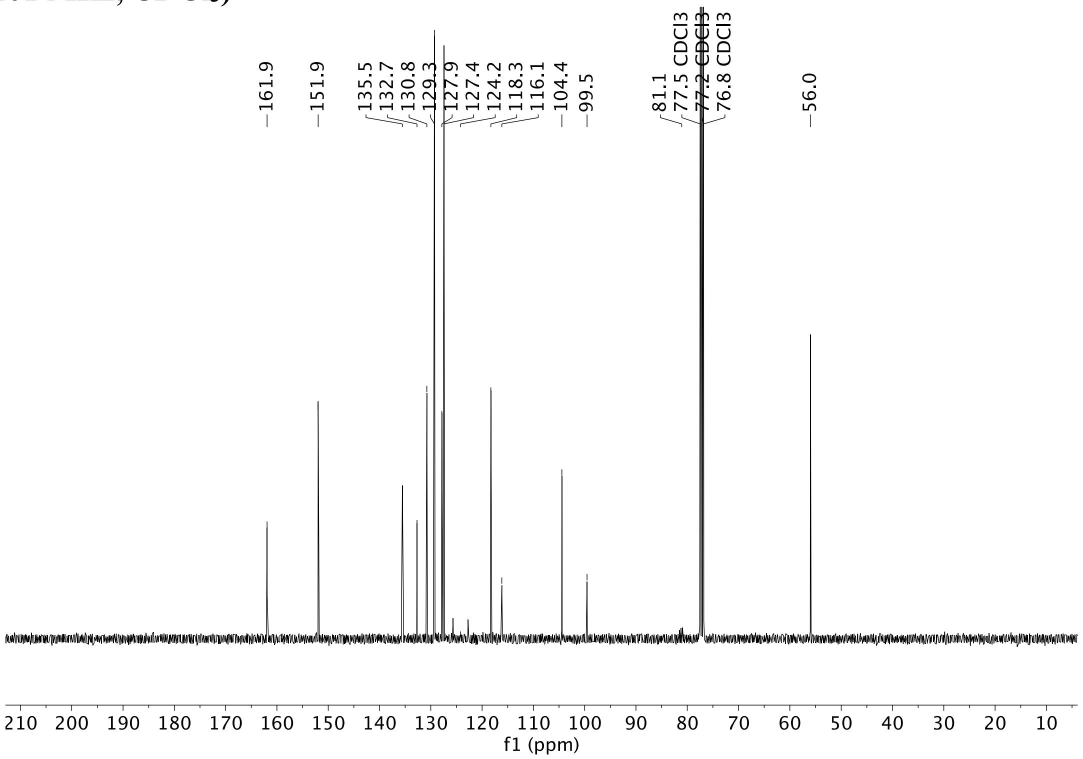

10.1.4 (*E*)-5,6-dimethyl-1-styryl-3,3-bis(trifluoromethyl)-1,3-dihydro-1λ³-benzo[*d*][1,2]iodaoxole (2d)

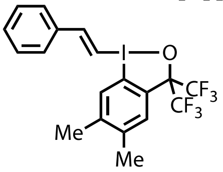

δH (400 MHz, CDCl3)

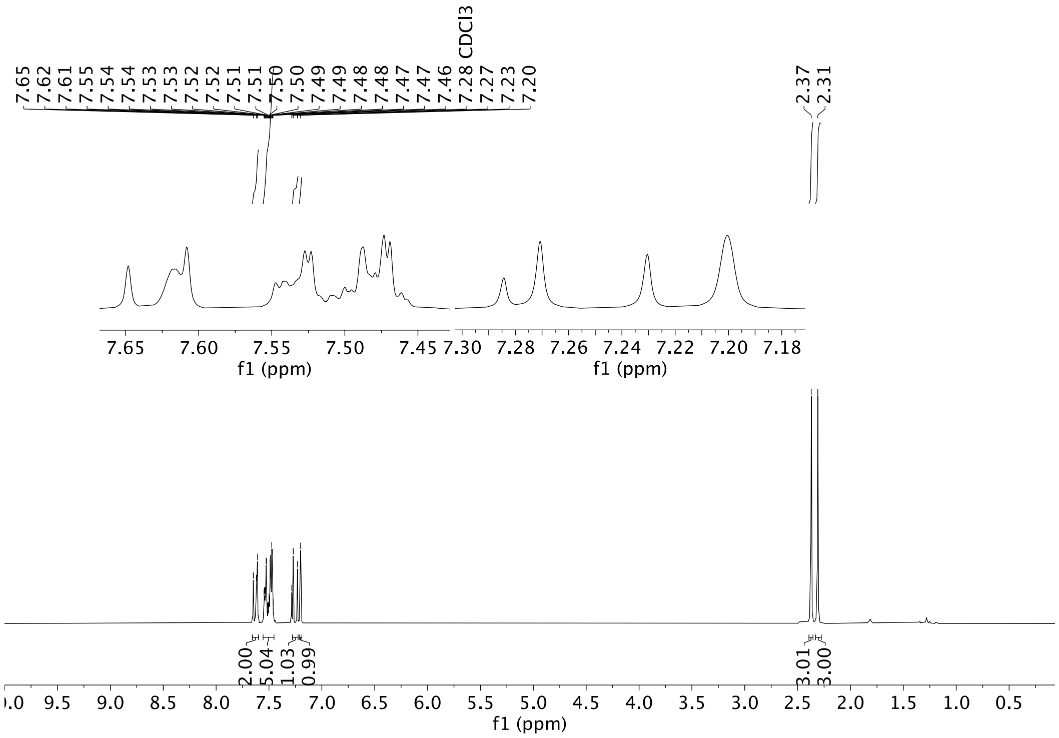

δF (376 MHz, CDCl3)

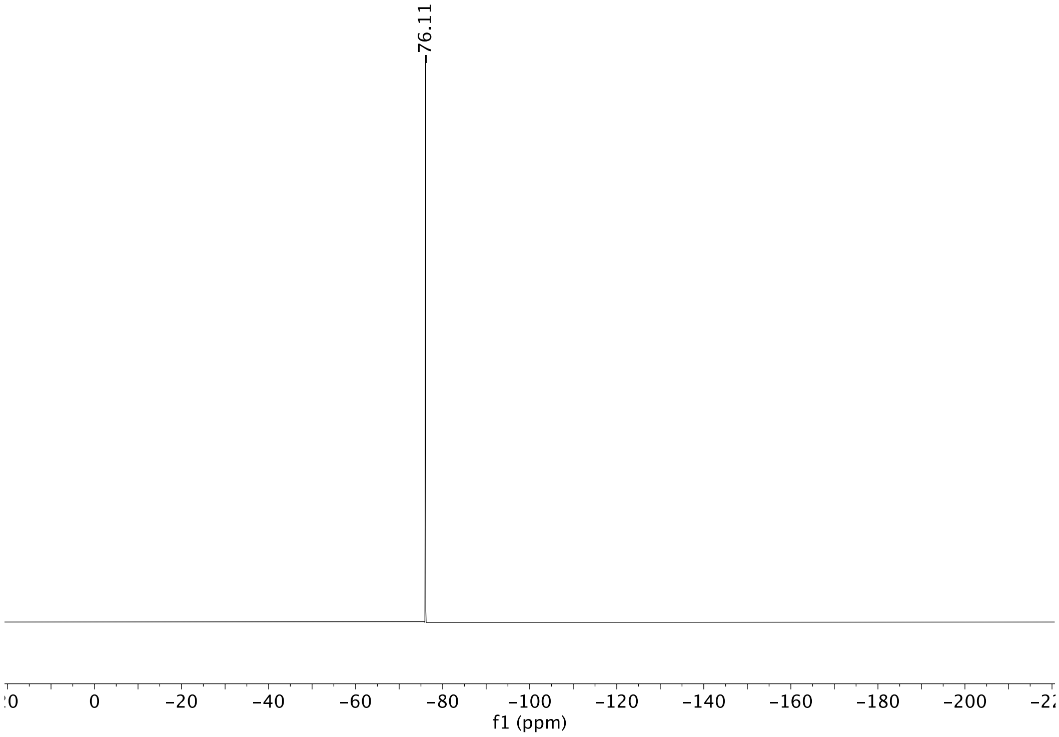

δC (101 MHz, CDCl3)

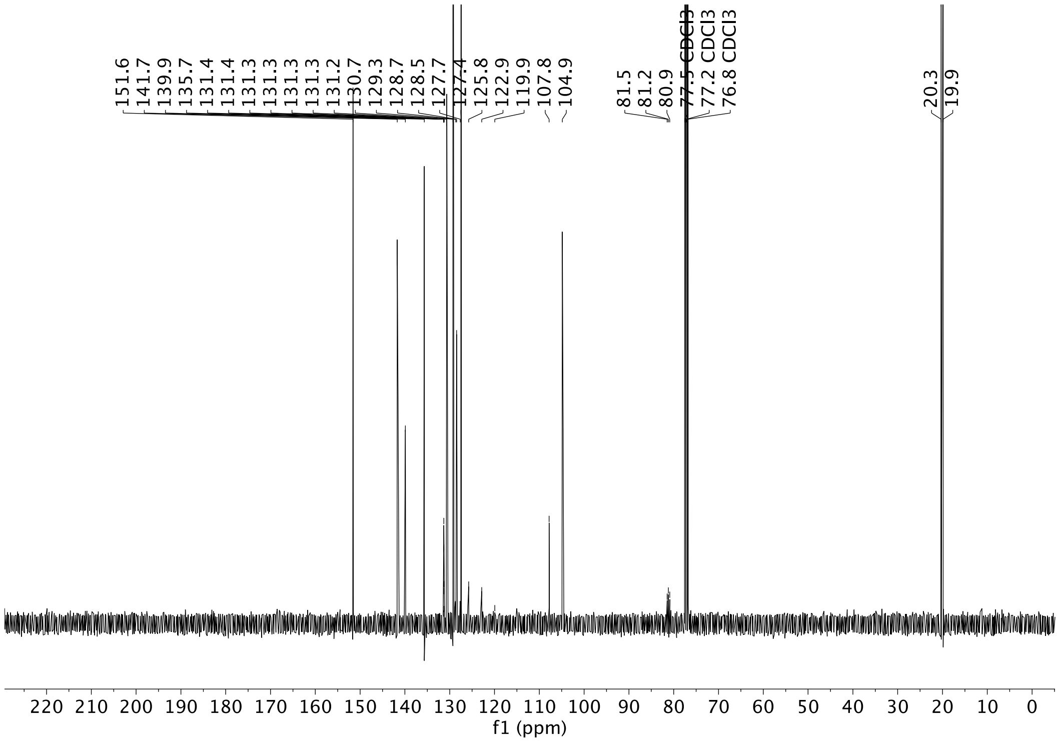

10.1.5 (*E*)-5-bromo-1-styryl-3,3-bis(trifluoromethyl)-1,3-dihydro-1λ^3-benzo[*d*][1,2]iodaoxole (2e)

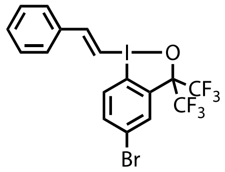

δH (400 MHz, CDCl3)

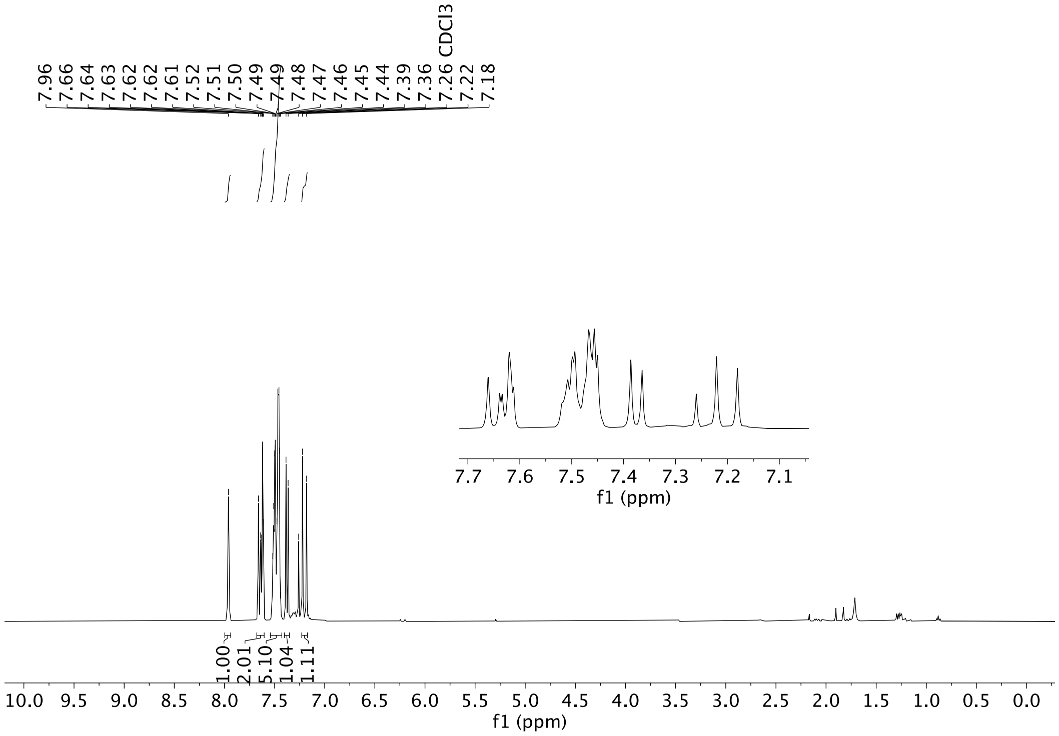

δF (376 MHz, CDCl3)

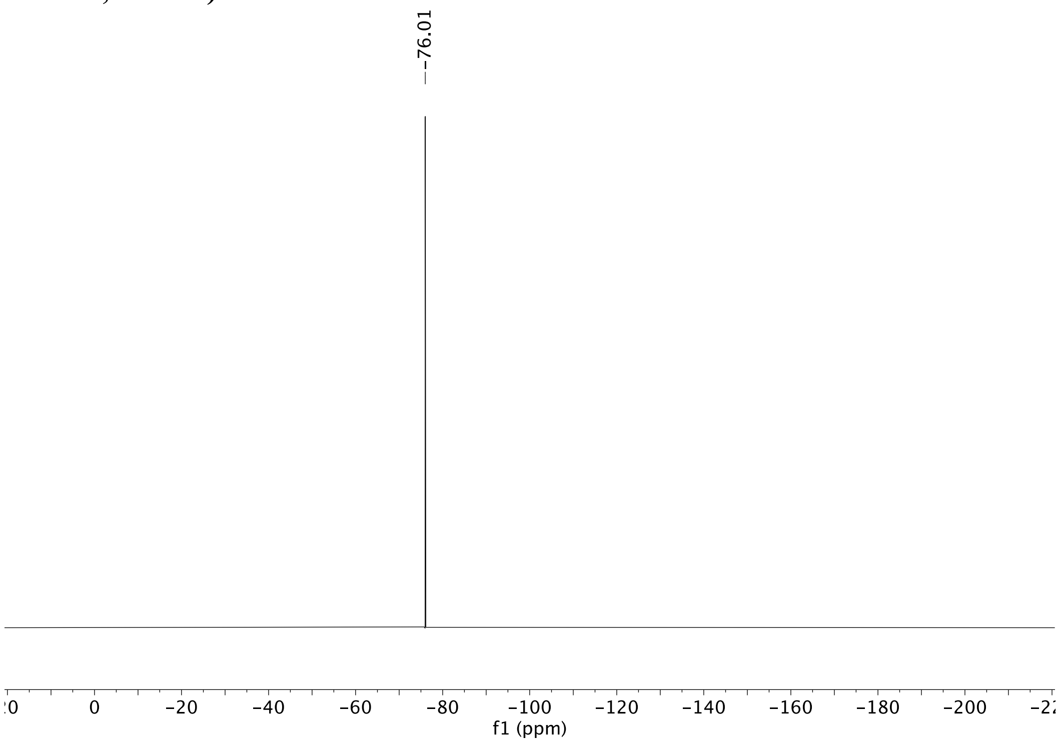

δ_C (101 MHz, $CDCl_3$)

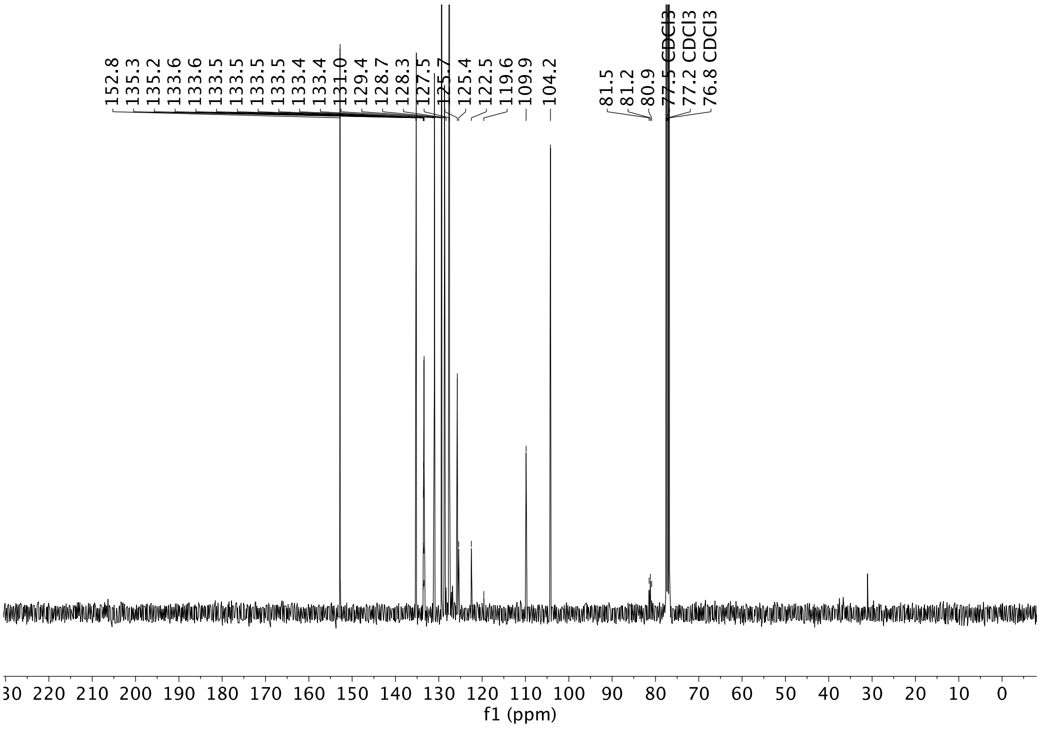

10.1.6 (*E*)-5,7-dimethyl-1-styryl-3,3-bis(trifluoromethyl)-1,3-dihydro-1λ^3-benzo[*d*][1,2]iodaoxole (2f)

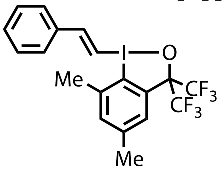

δH (500 MHz, CDCl3)

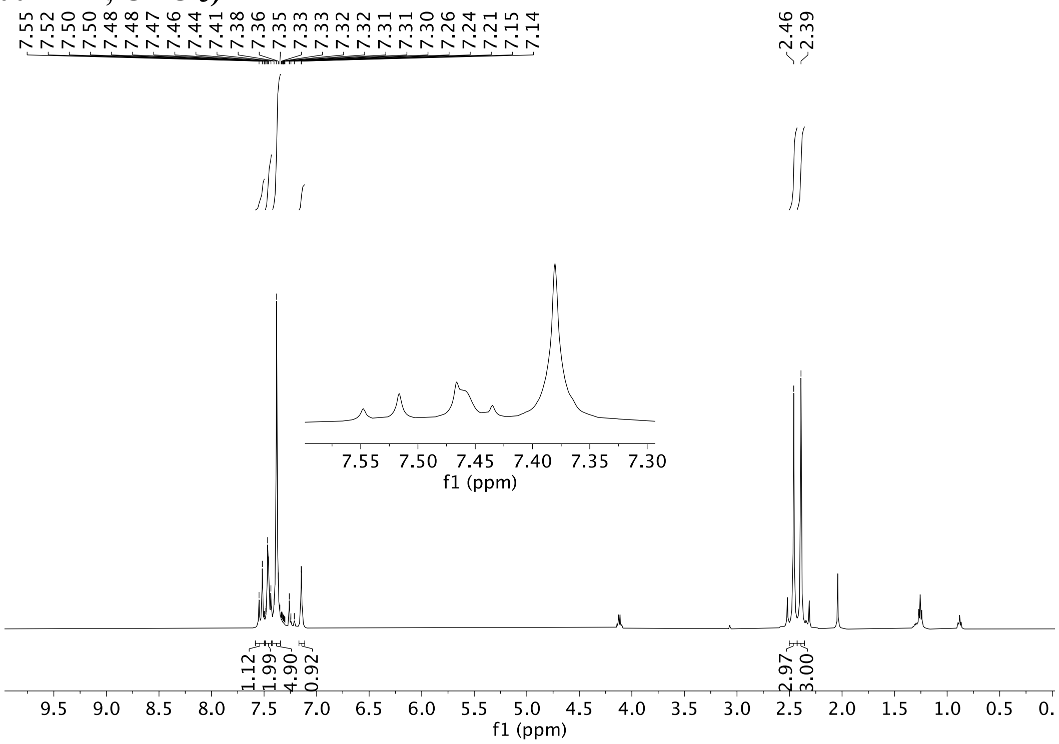

δF (376 MHz, CDCl3)

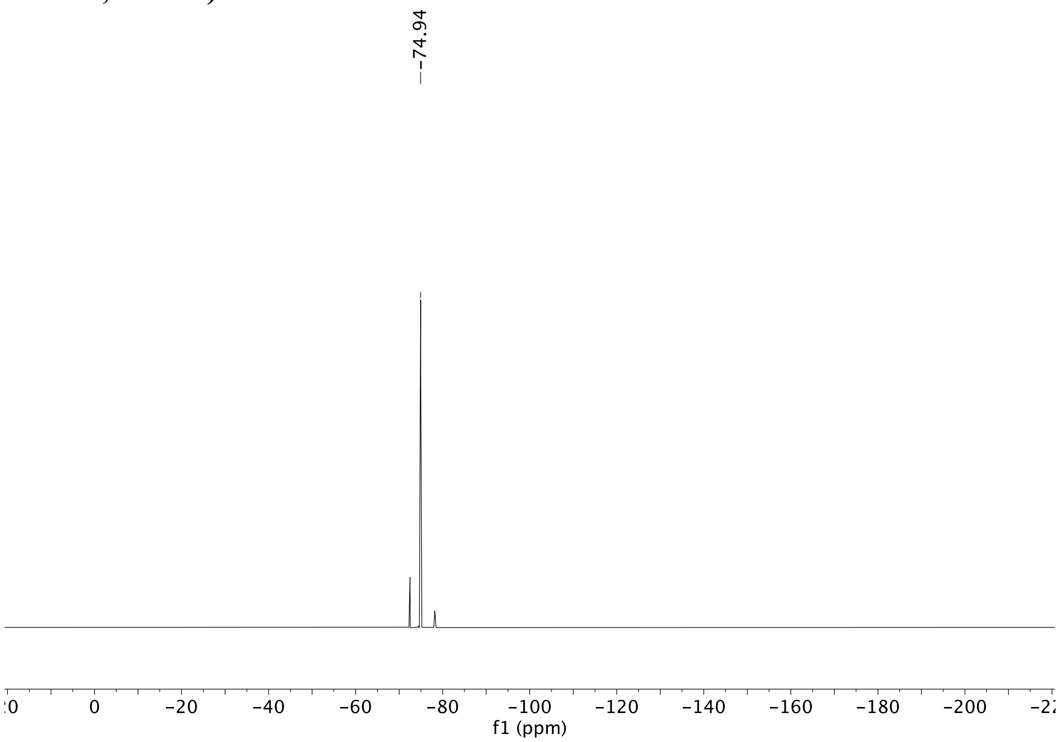

δC (125 MHz, CDCl3)

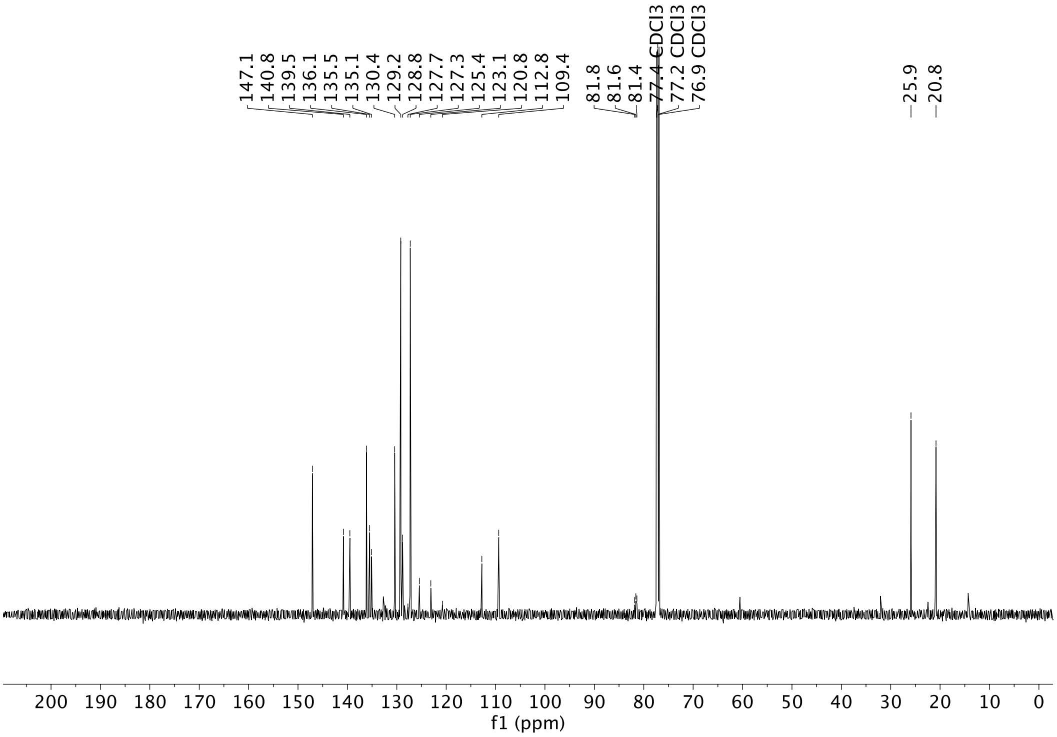

10.1.7 (*E*)-1-styryl-2-tosyl-1,2-dihydro-3H-1λ^3-benzo[*d*][1,2]iodazol-3-one (3a)

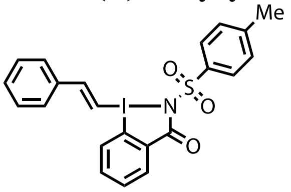

δ_H (400 MHz, DMSO-d$_6$)

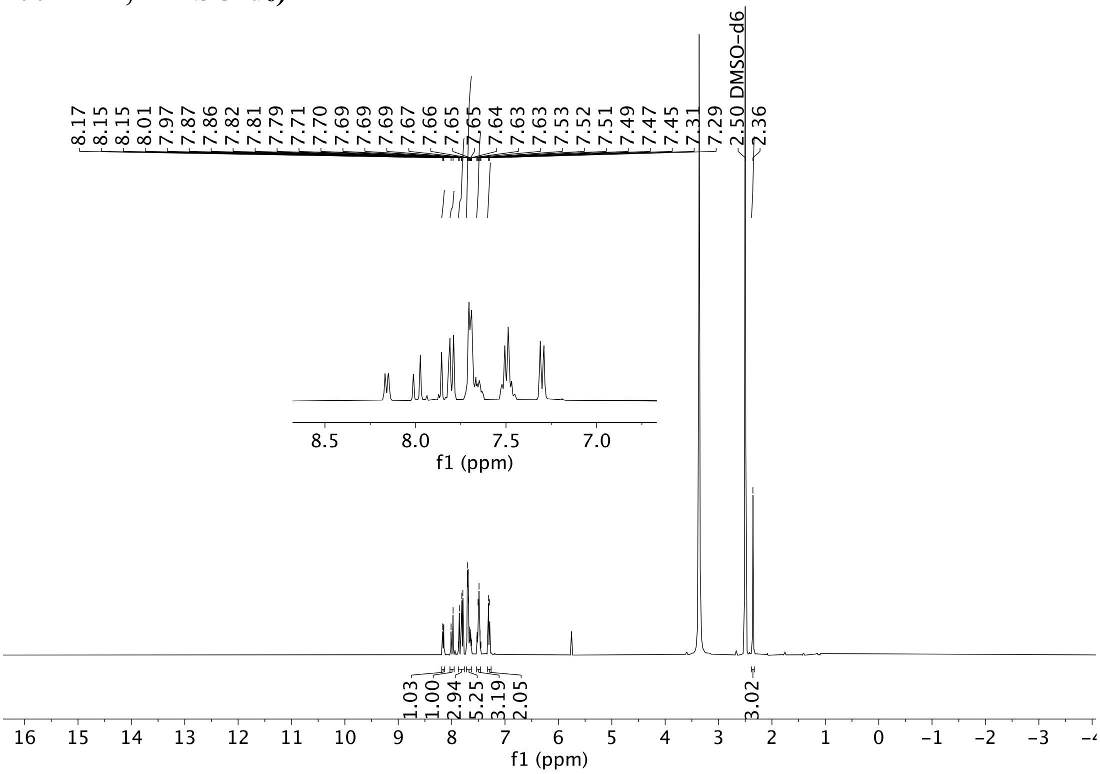

δ_C (101 MHz, DMSO-d$_6$)

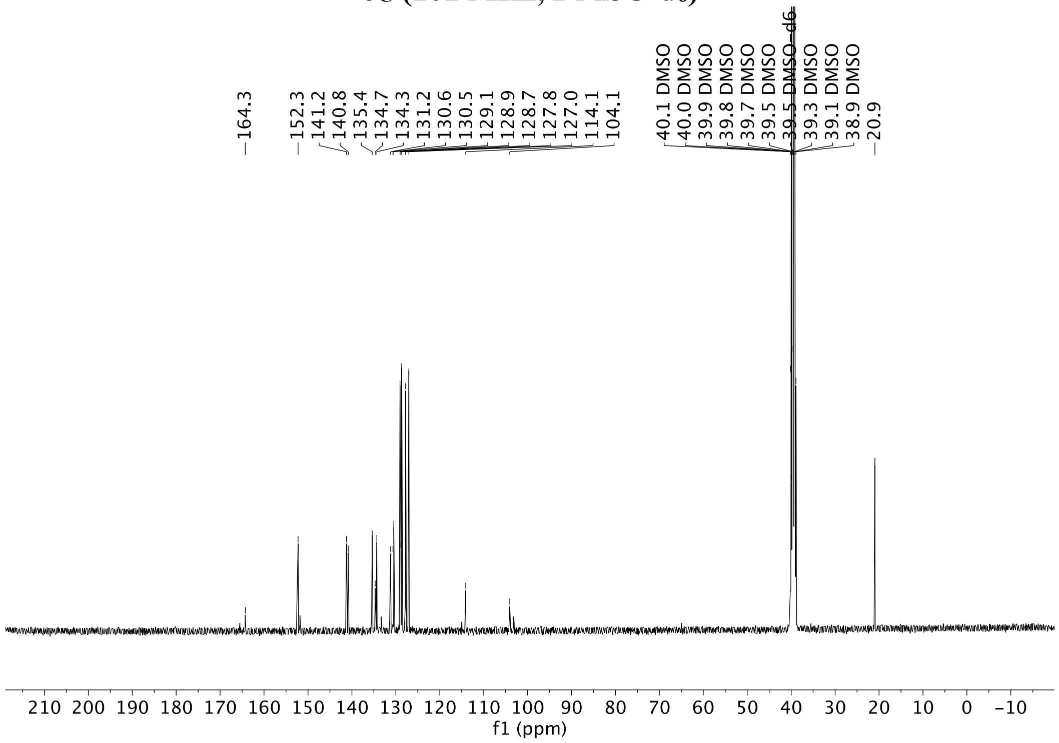

10.1.8 (*E*)-5-methyl-1-styryl-2-tosyl-1,2-dihydro-3H-1λ^3-benzo[*d*][1,2]iodazol-3-one (3b)

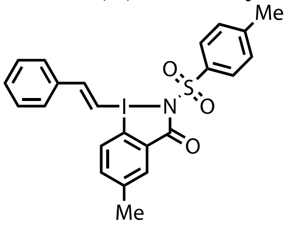

δH (400 MHz, DMSO-d6)

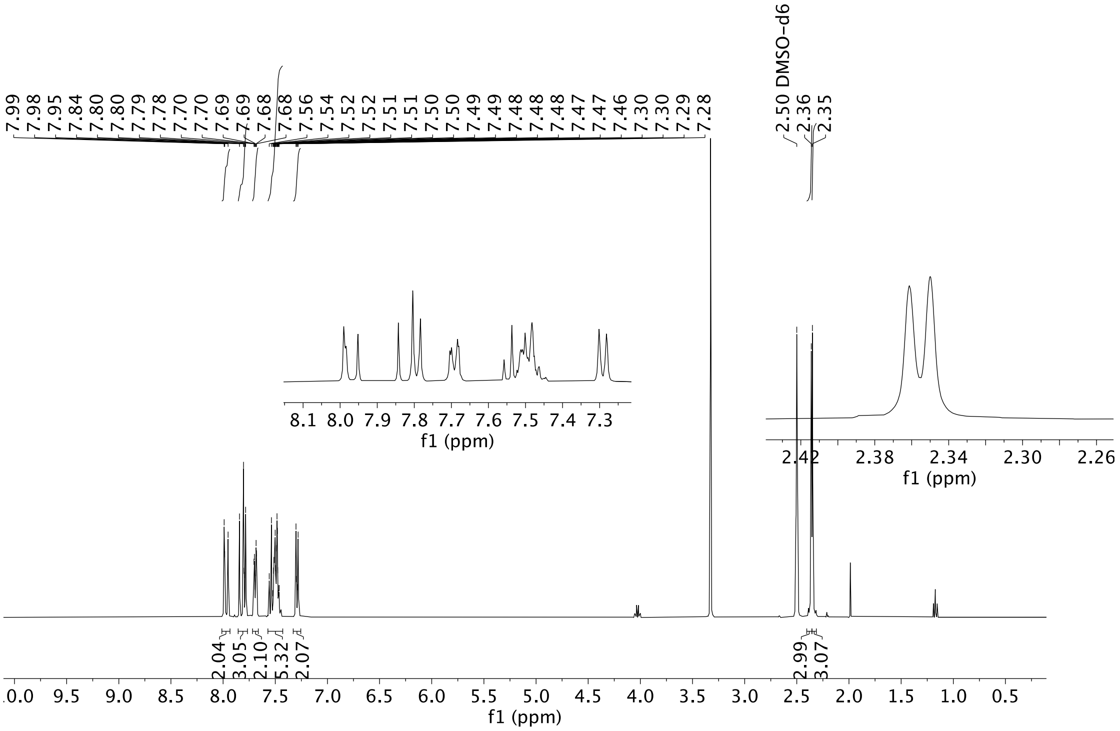

δC (101 MHz, DMSO-d6)

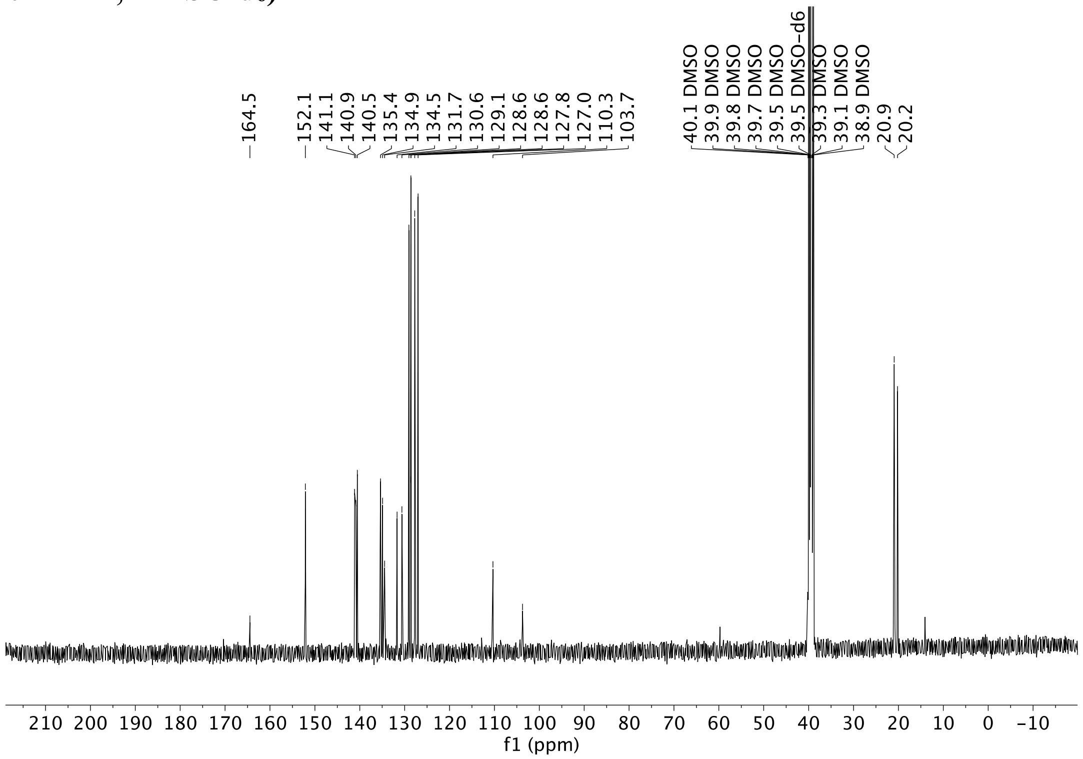

10.1.9 (*E*)-5-bromo-1-styryl-2-tosyl-1,2-dihydro-3H-1λ^3-benzo[*d*][1,2]iodazol-3-one (3c)

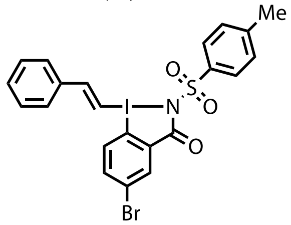

δ$_H$ (400 MHz, DMSO-d$_6$)

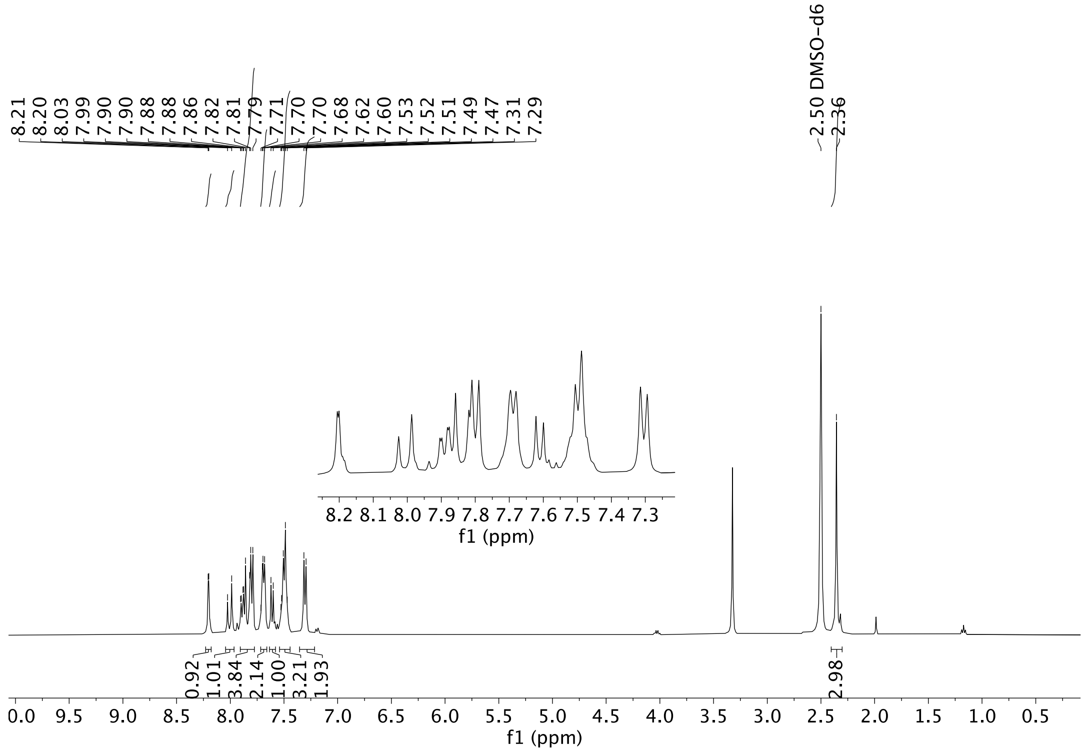

δ$_C$ (101 MHz, DMSO-d$_6$)

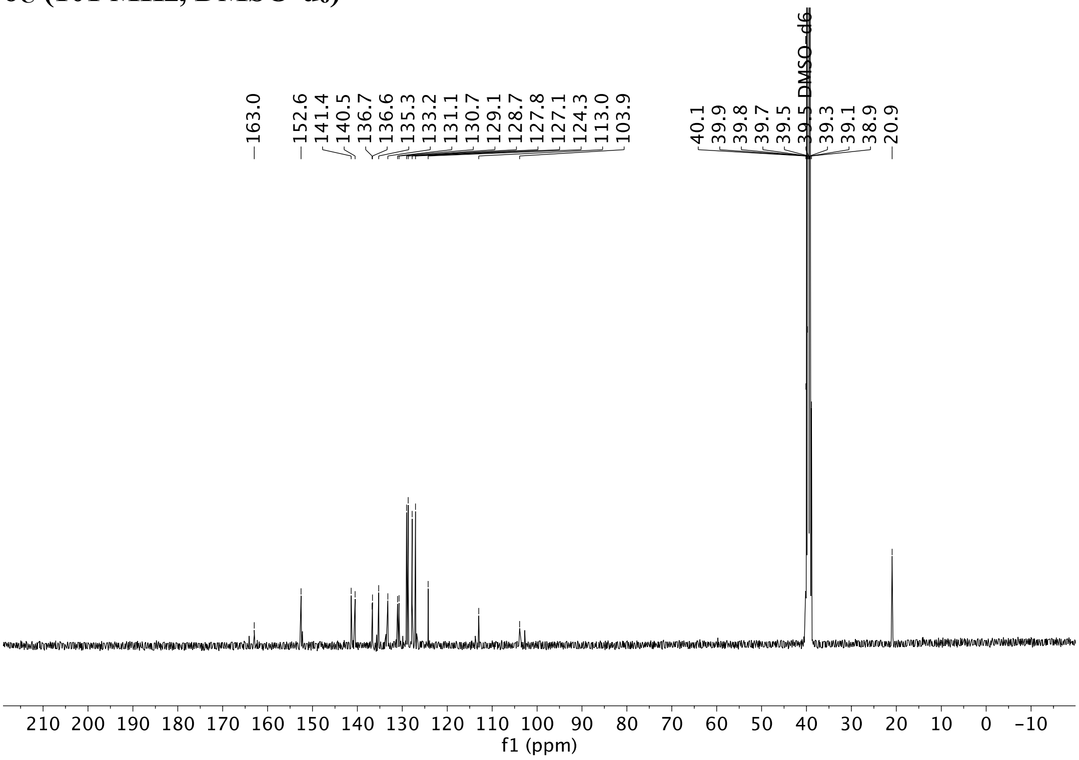

10.1.10(*E*)-4-methyl-*N*-((*Z*)-7-methyl-1-((*E*)-styryl)-1λ^3 -benzo[*d*][1,2]iodaoxol-3(1*H*)-ylidene)benzenesulfonamide (4)

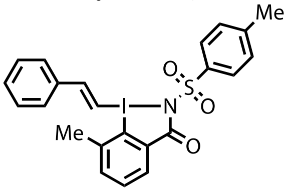

δ$_H$ (400 MHz, CDCl$_3$)

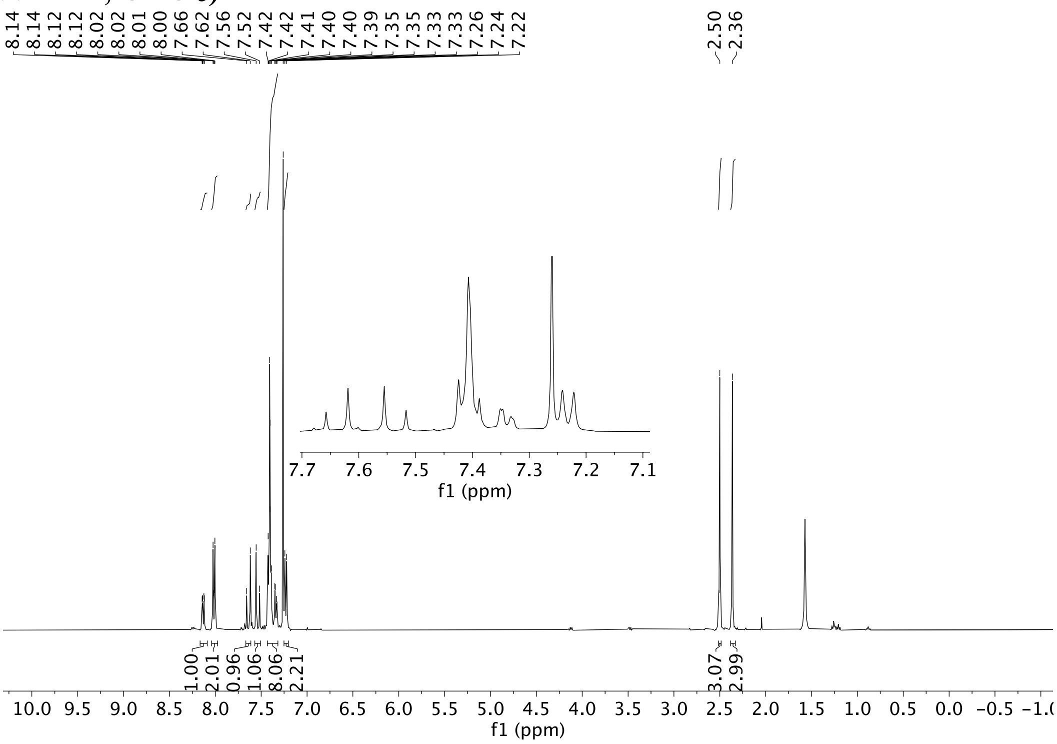

δ$_C$ (125 MHz, DMSO-d$_6$)

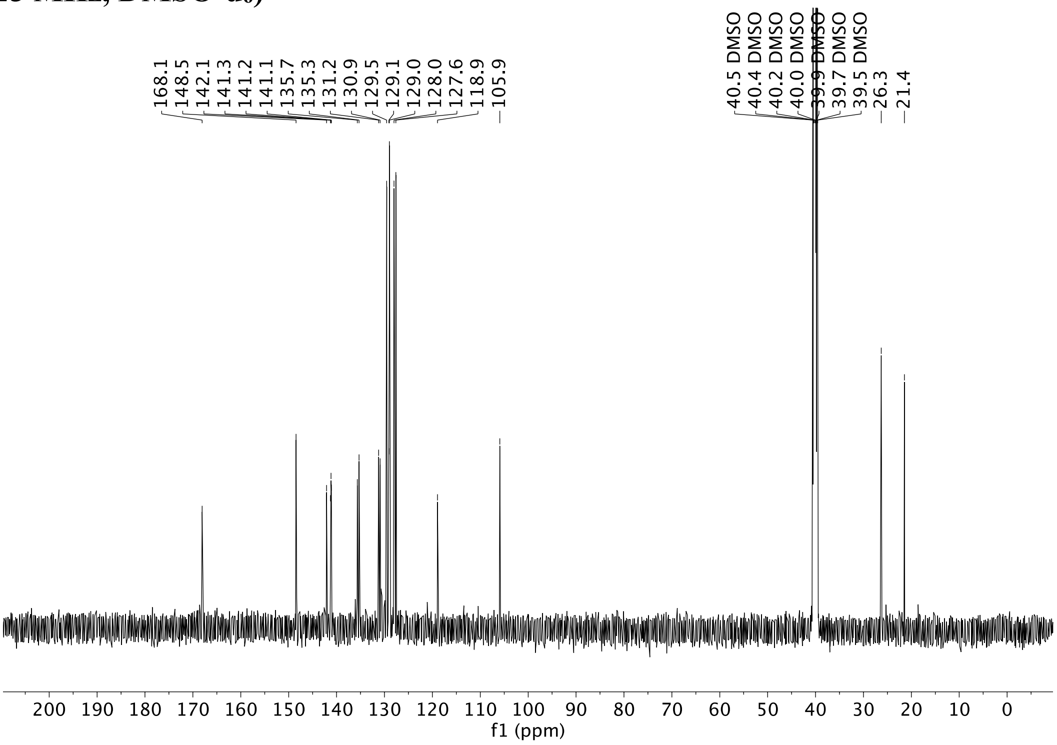

10.1.11(*E*)-1-styryl-1H-1λ^3-benzo[*d*][1,2,3]iodaoxathiole 3,3-dioxide (5a)

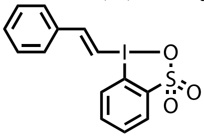

δ$_H$ (400 MHz, DMSO-d$_6$)

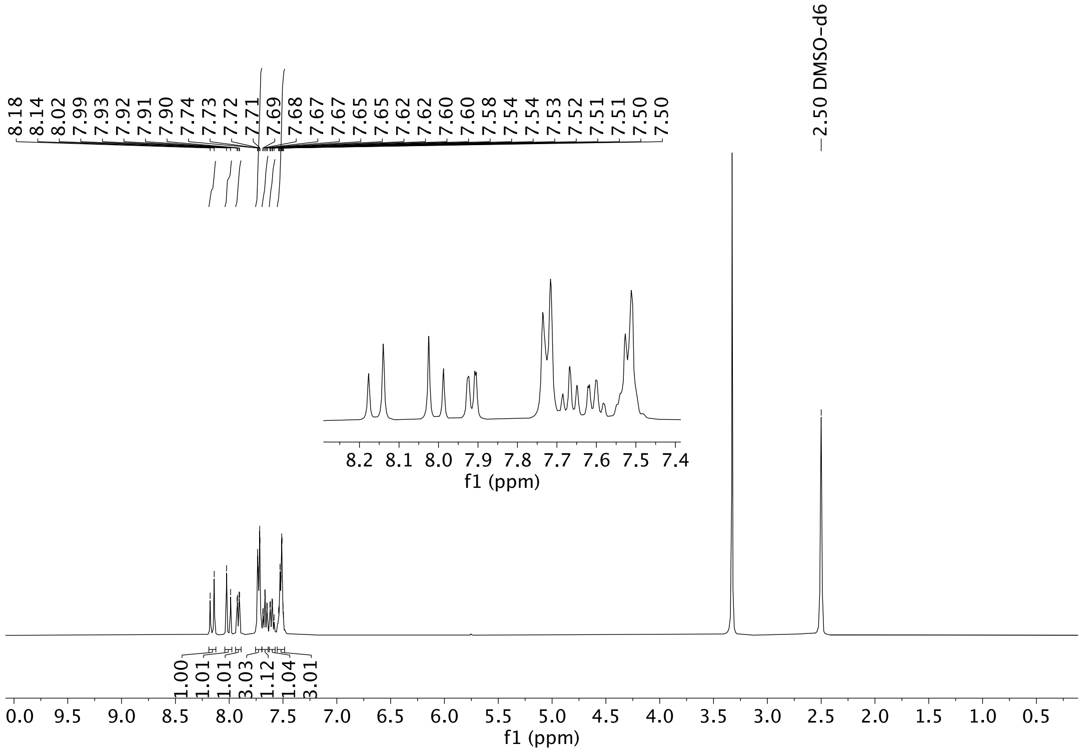

δ$_C$ (101 MHz, DMSO-d$_6$)

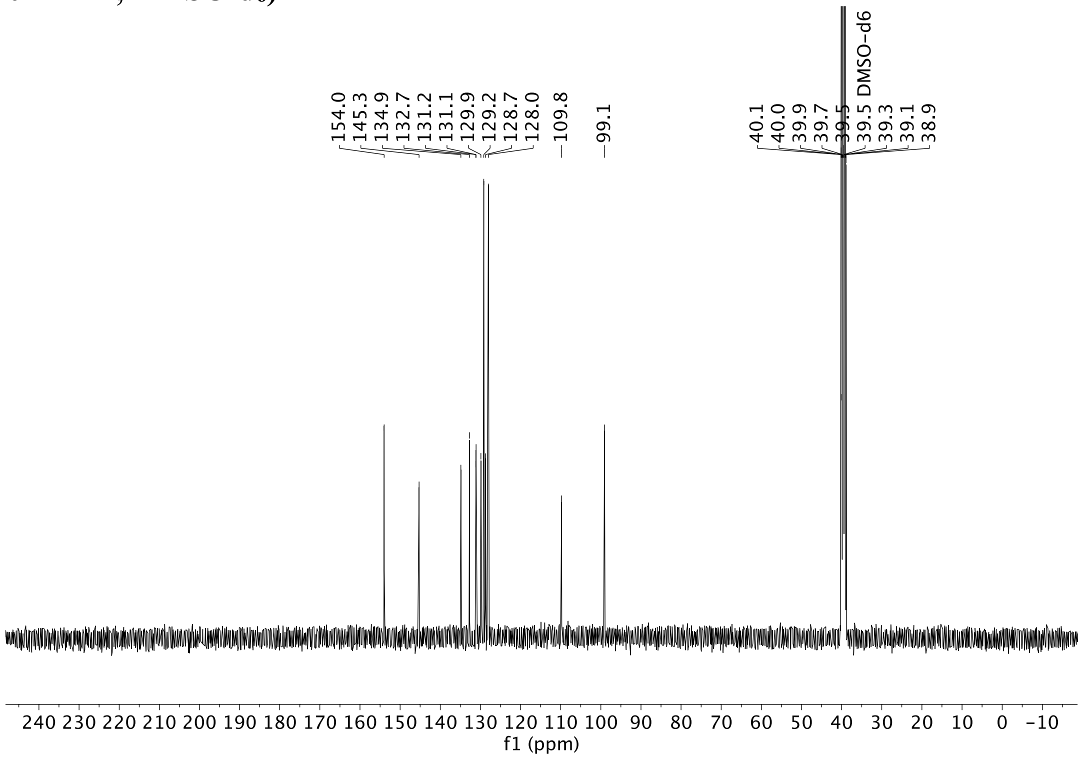

10.1.12 (*E*)-5-methyl-1-styryl-1H-1λ[3]-benzo[*d*][1,2,3]iodaoxathiole 3,3-dioxide (5b)

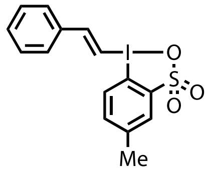

δ_H (400 MHz, DMSO-d$_6$)

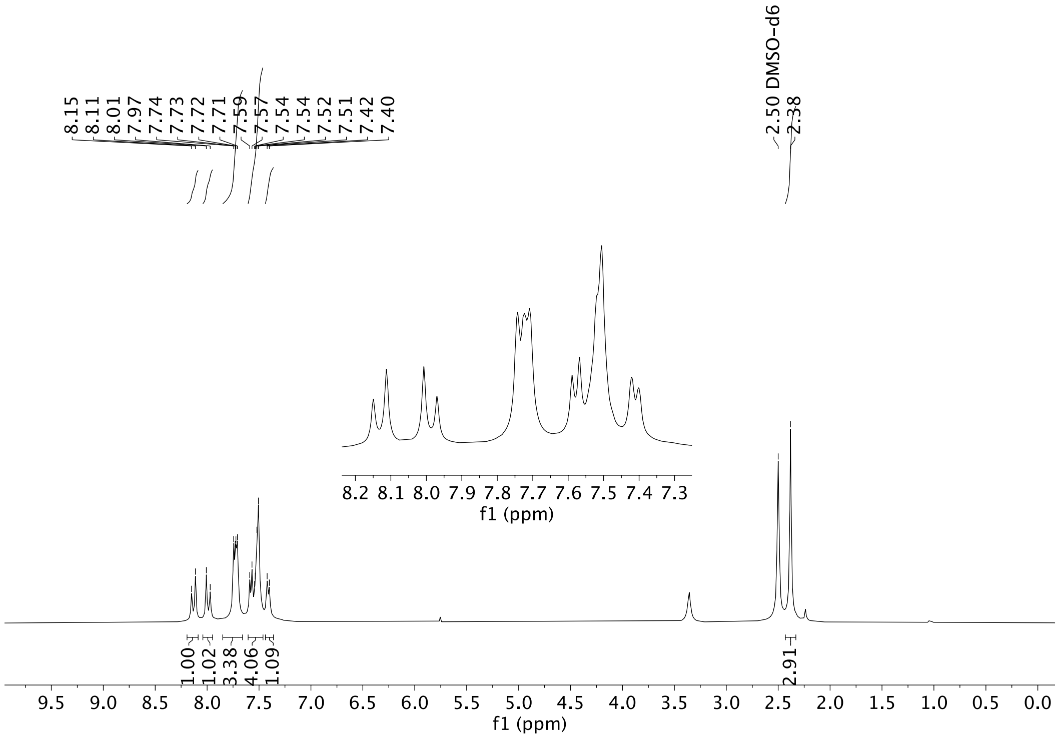

δ_C (101 MHz, DMSO-d$_6$)

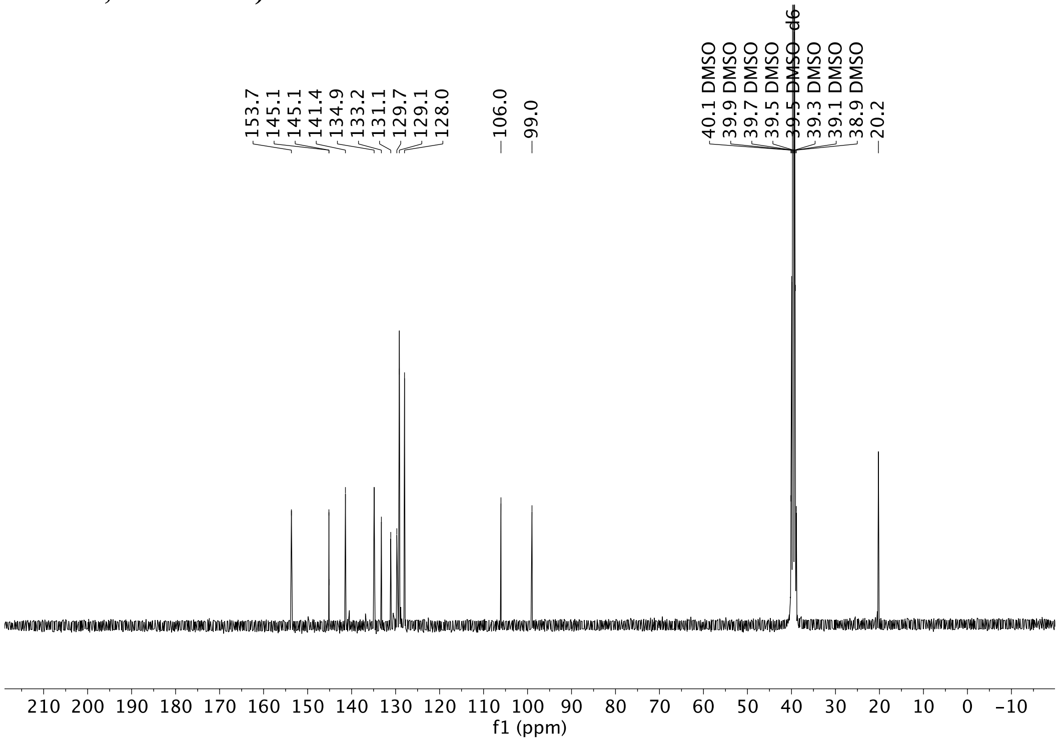

10.1.13 (*E*)-(2-(methoxycarbonyl)phenyl)(styryl)iodonium trifluoromethanesulfonate (6a)

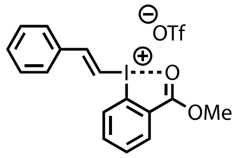

δ$_H$ (400 MHz, MeOD-d$_4$)

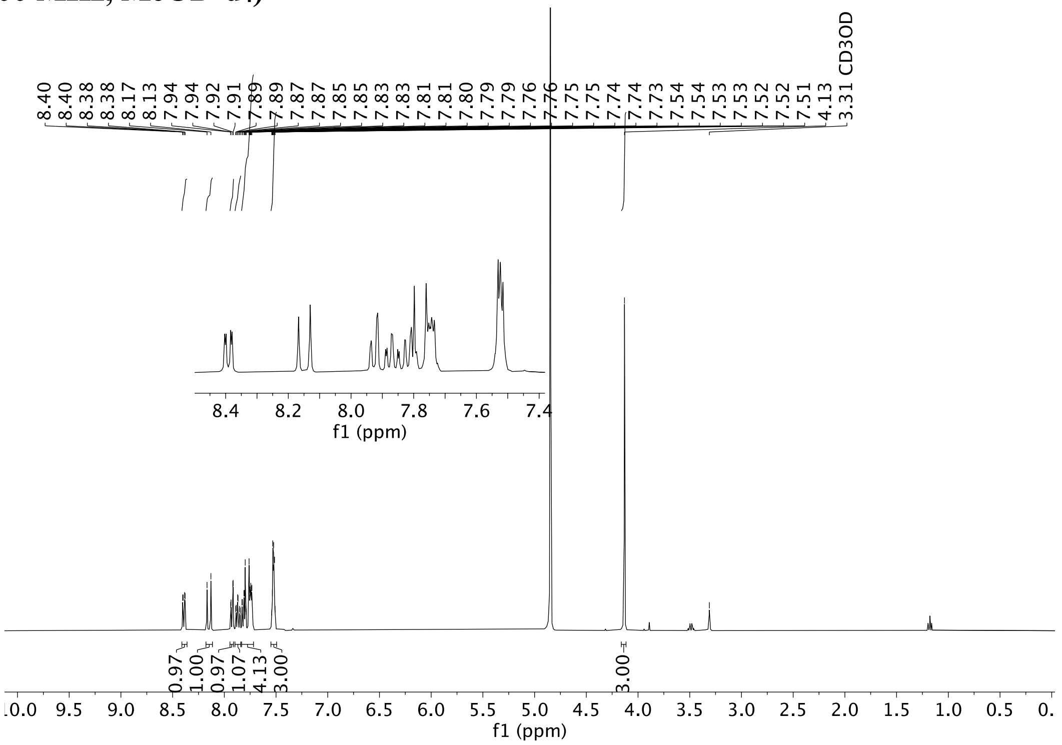

δ$_F$ (376 MHz, MeOD-d$_4$)

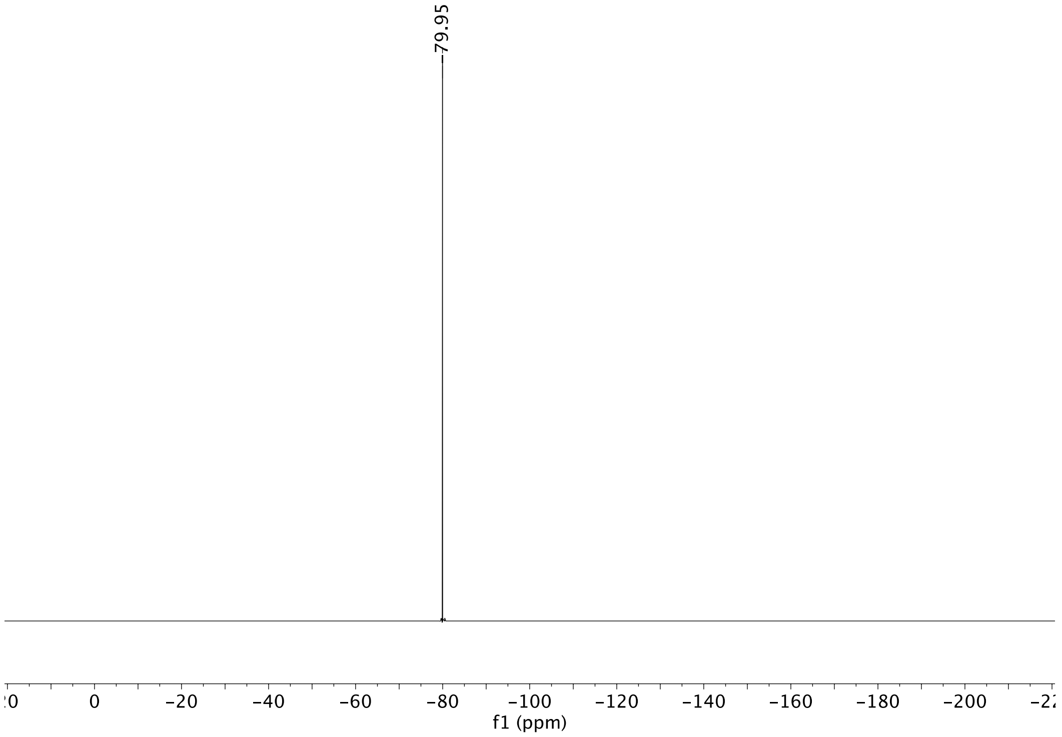

δ_C (101 MHz, MeOD-d4)

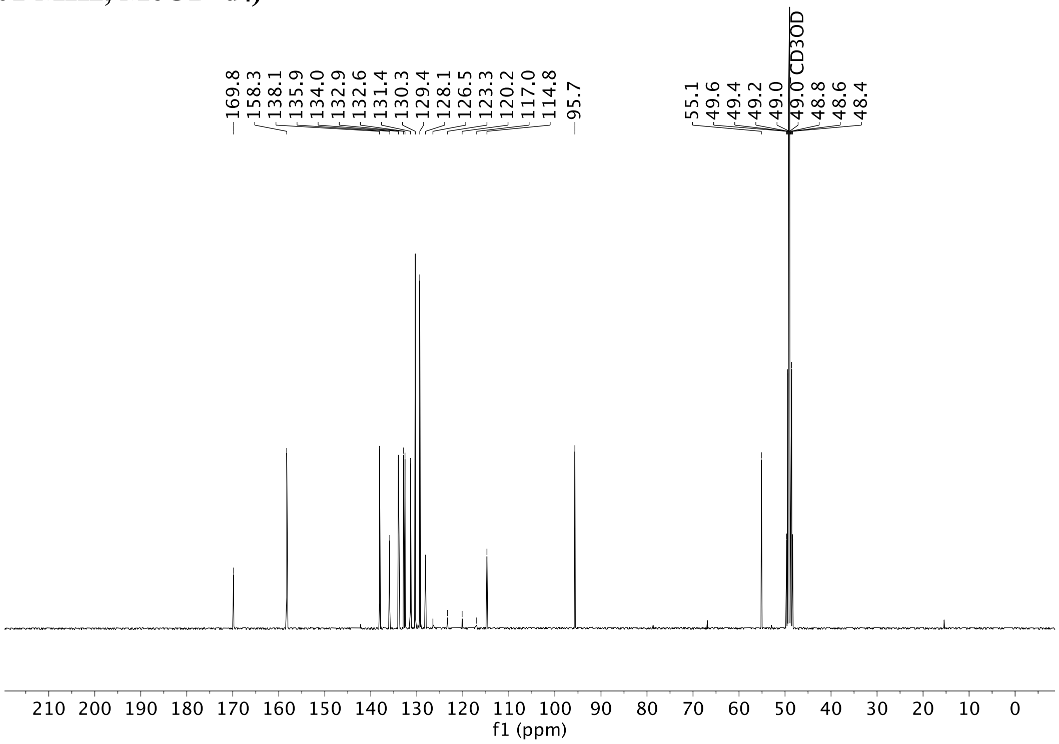

10.1.14(*E*)-(4-methyl-2-sulfophenyl)(styryl)iodonium trifluoromethanesulfonate (6b)

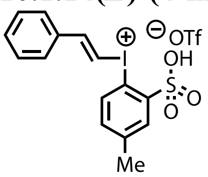

δ_H (400 MHz, DMSO-d$_6$)

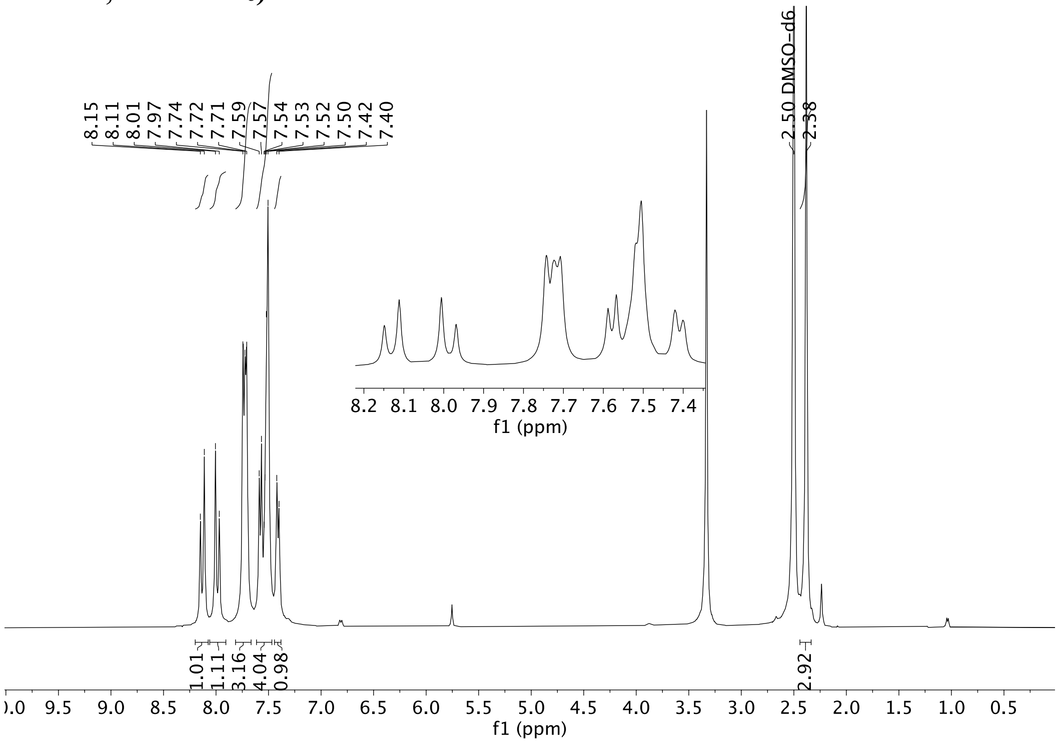

δ_F (376 MHz, DMSO-d$_6$)

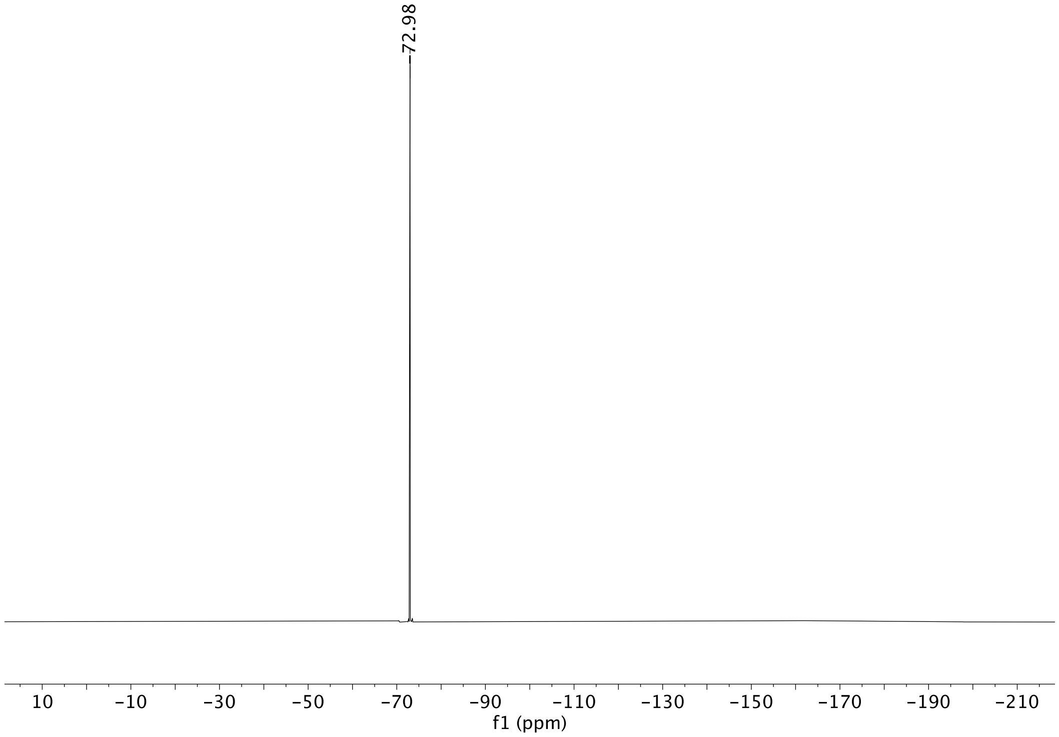

δC (101 MHz, DMSO-d6)

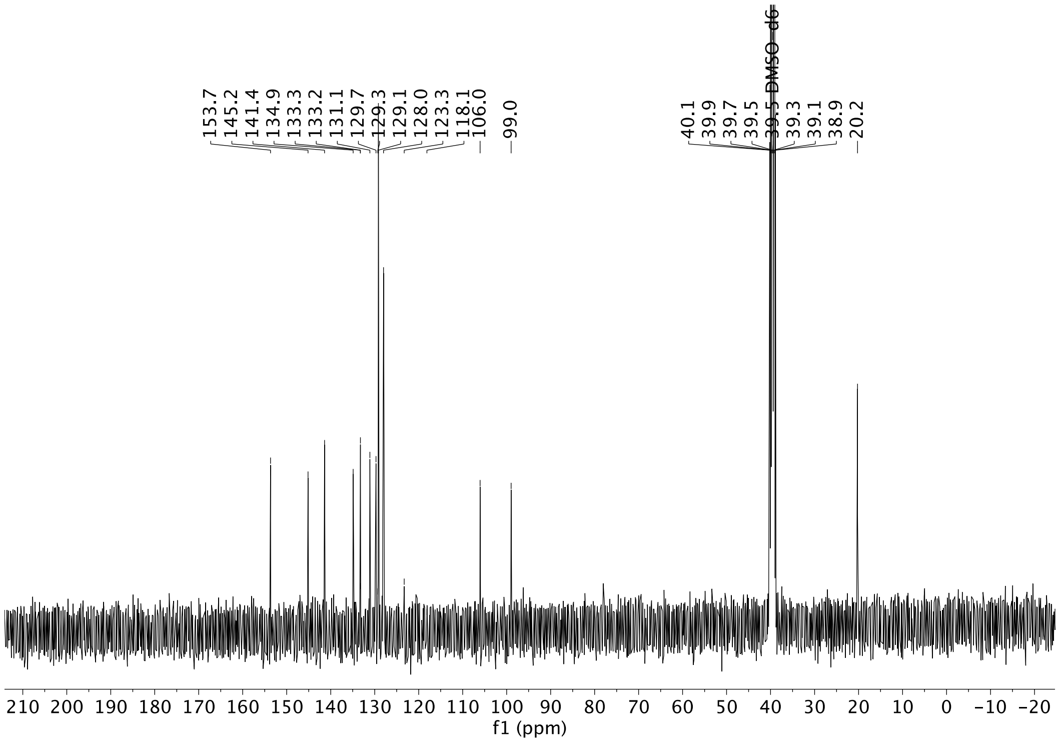

10.1.15(*E*)-(4-bromo-2-carboxyphenyl)(styryl)iodonium trifluoromethanesulfonate (6c)

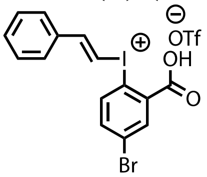

δ_H (400 MHz, MeOD-d$_4$)

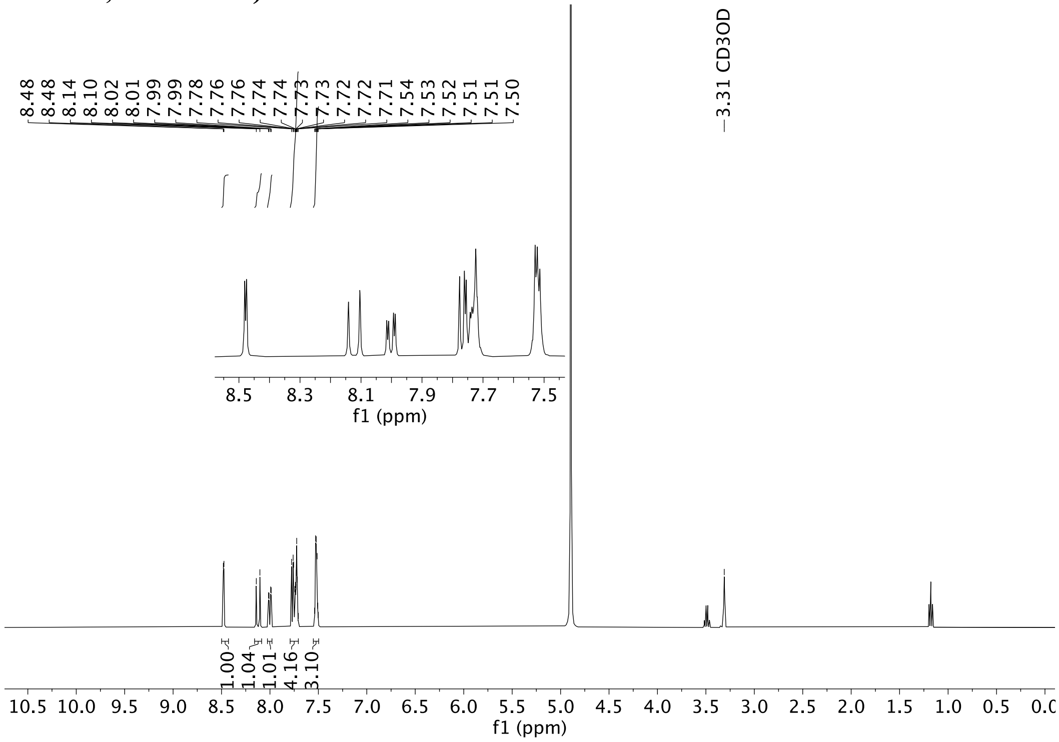

δ_F (376 MHz, MeOD-d$_4$)

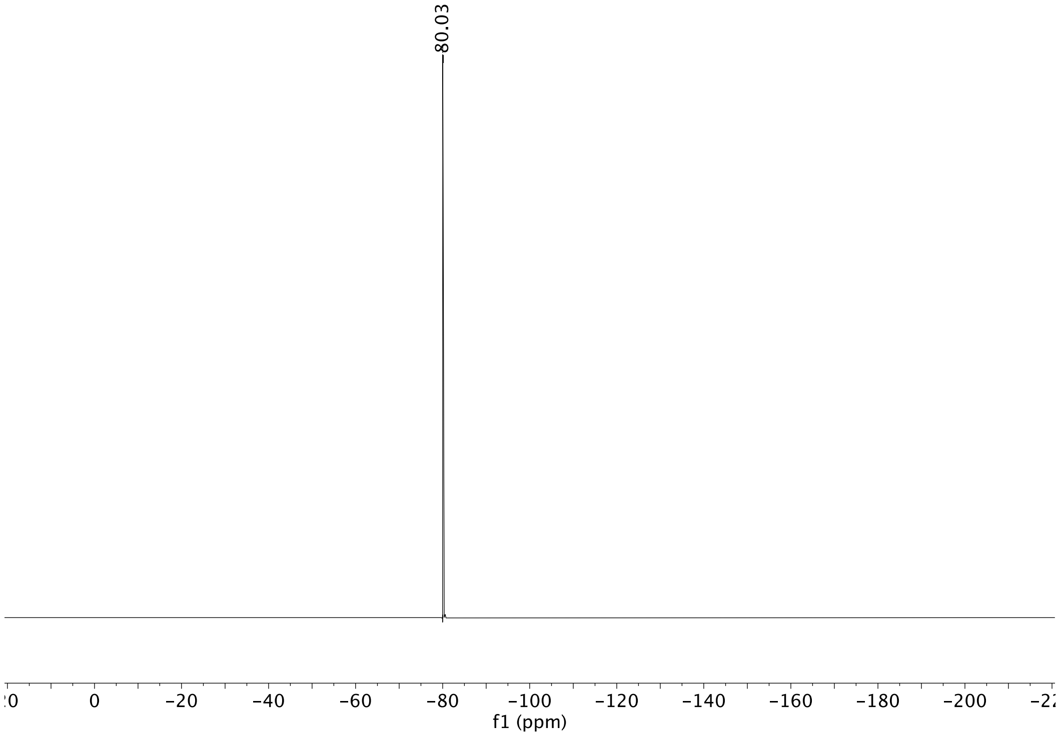

δC (101 MHz, MeOD-d4)

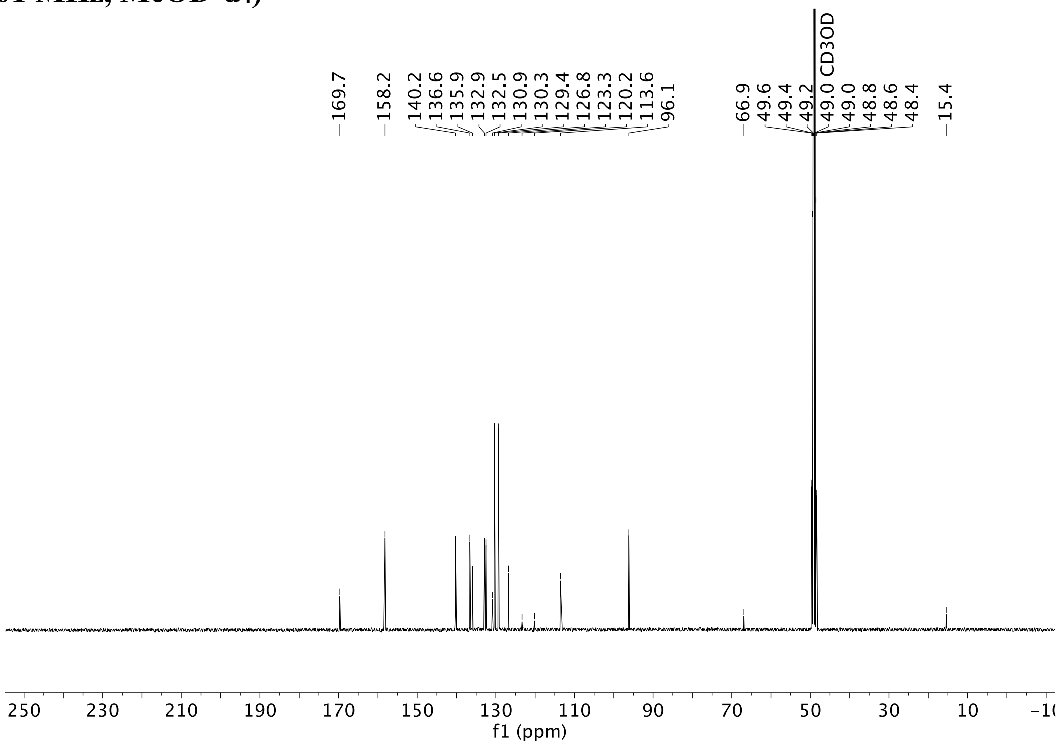

10.1.16(*E*)-3-(2-(styryl-1λ^3-iodaneyl)phenyl)propanoic acid trifluoromethanesulfonate (6d)

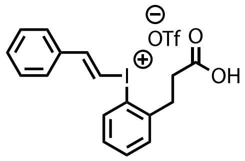

δ$_H$ (400 MHz, MeOD-d$_4$)

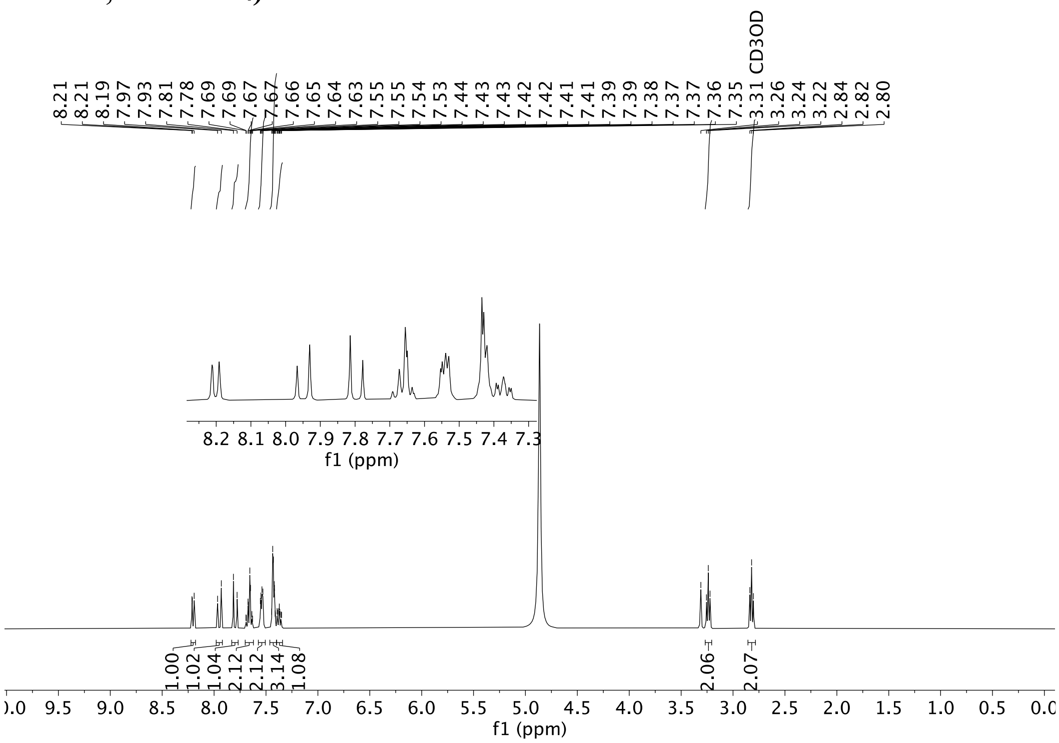

δ$_F$ (376 MHz, MeOD-d$_4$)

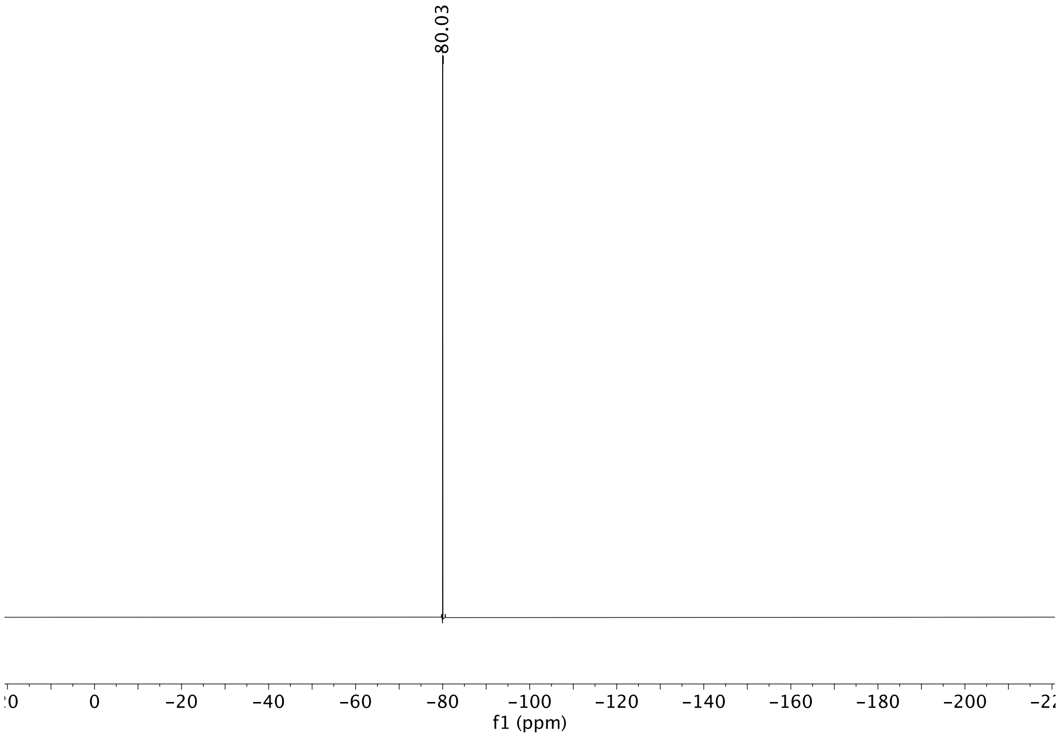

δC (101 MHz, MeOD-d4)

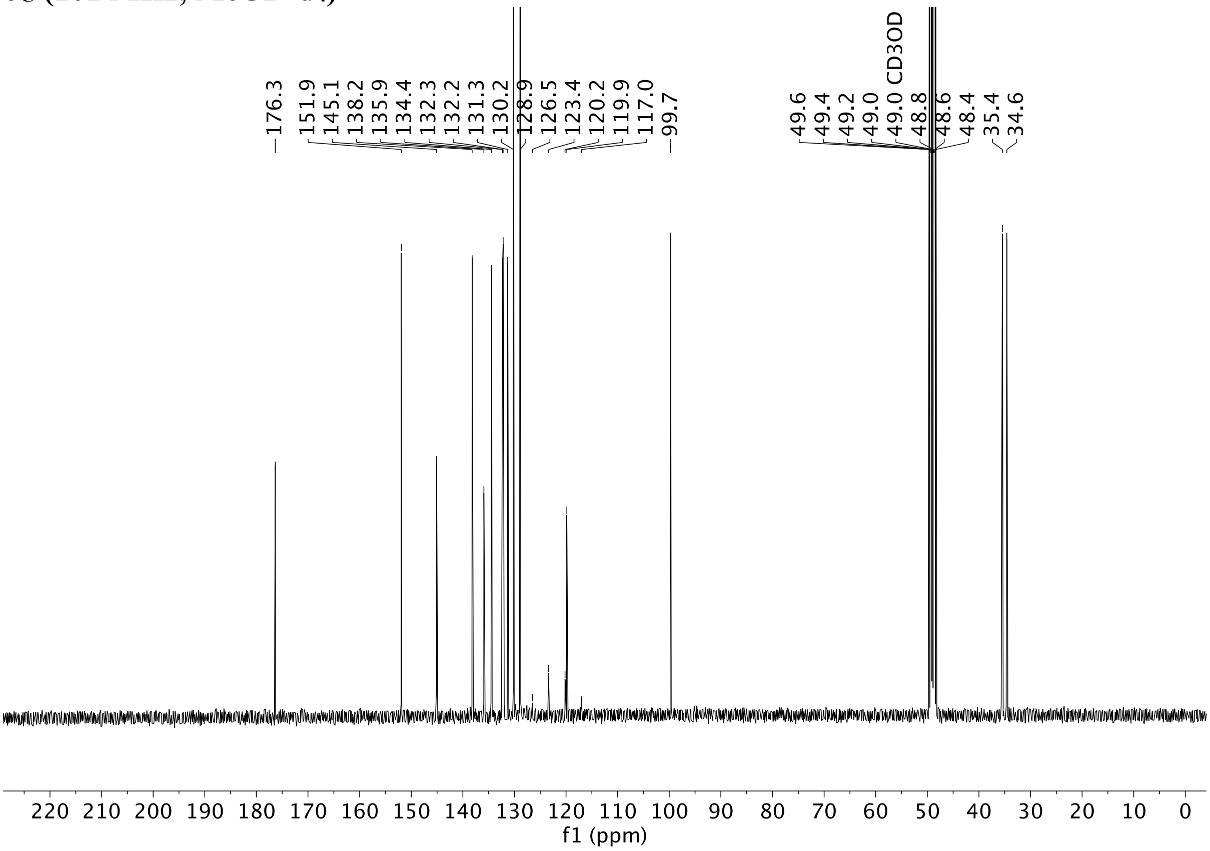

10.1.17(*E*)-(2-(carboxymethyl)phenyl)(styryl)iodonium trifluoromethanesulfonate (6e)

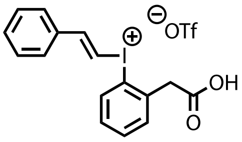

δH (400 MHz, DMSO-d6)

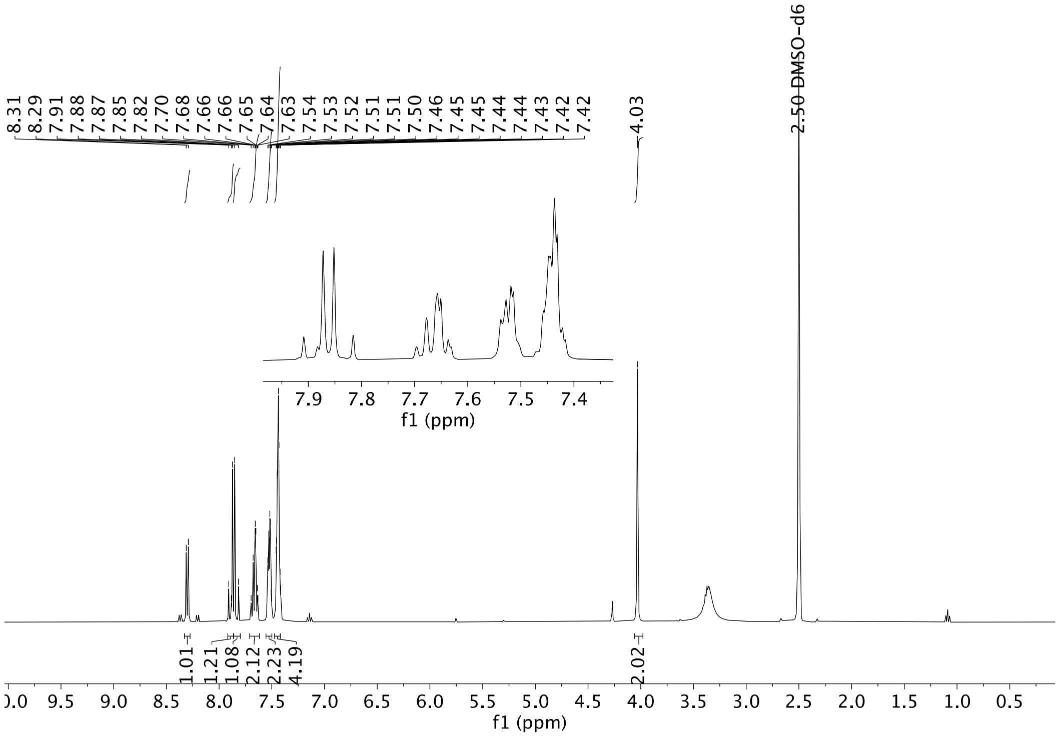

δF (376 MHz, DMSO-d6)

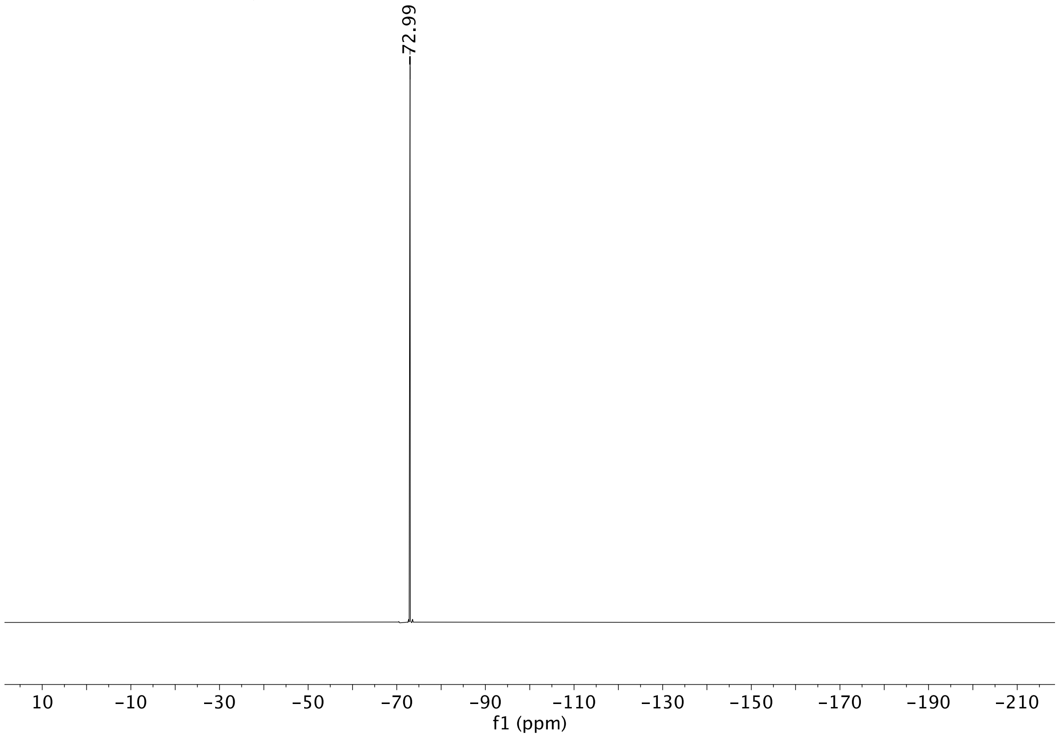

δC (101 MHz, DMSO-d6)

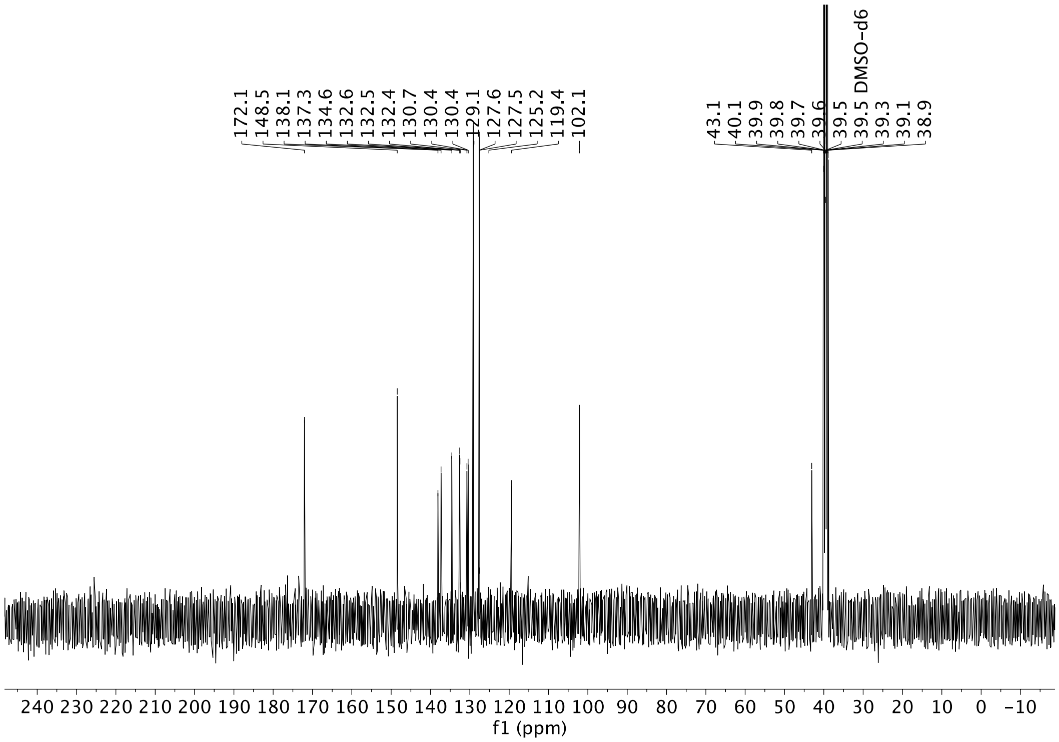

10.1.18(*E*)-1-styryl-1,4-dihydro-3H-1λ^3-benzo[*d*][1,2]iodaoxin-3-one (7)

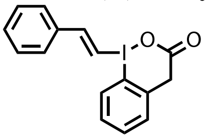

δ$_H$ (400 MHz, MeOD-d$_4$)

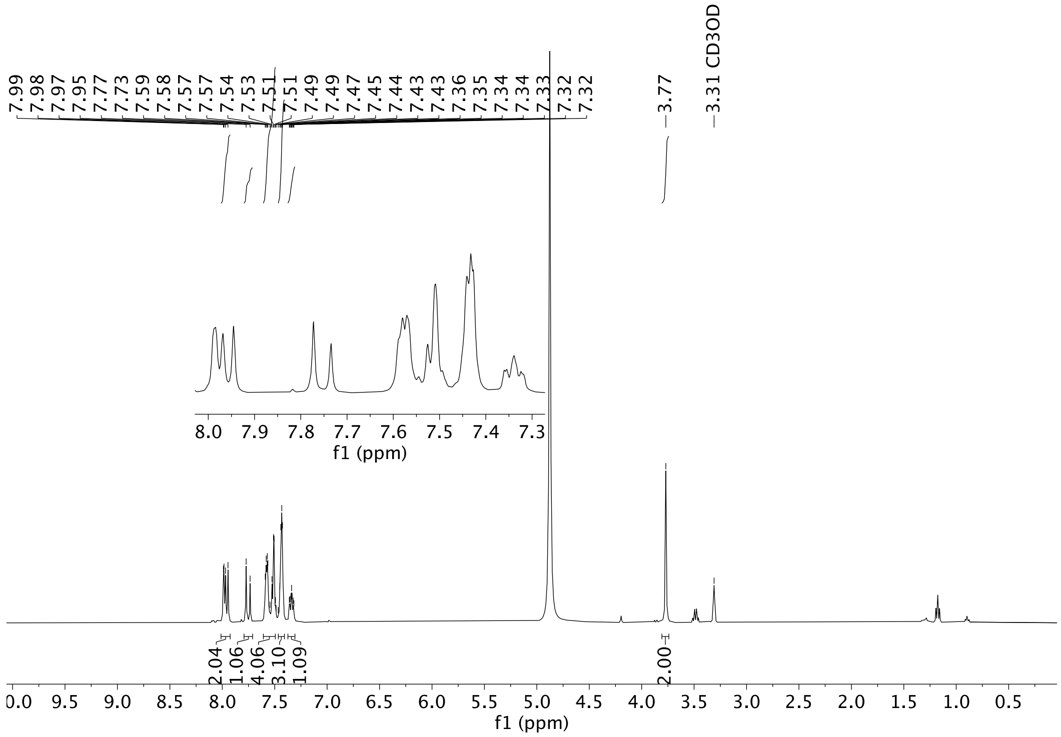

δ$_C$ (101 MHz, MeOD-d$_4$)

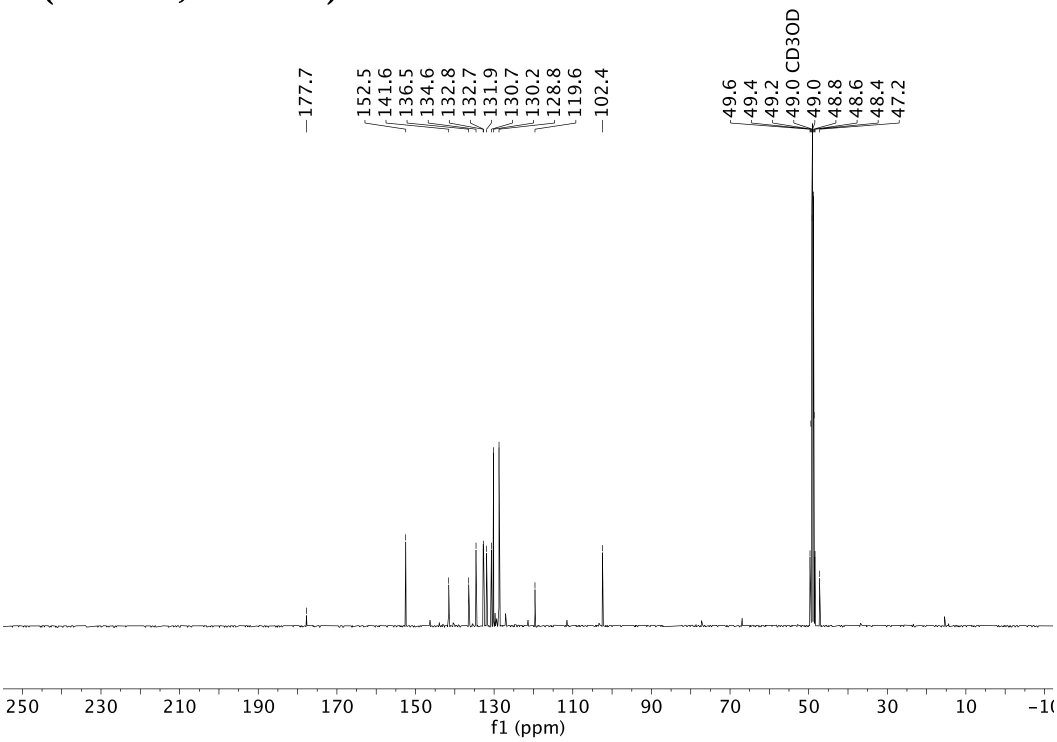

10.1.19(E)-(4-bromophenyl)(styryl)sulfane (14)

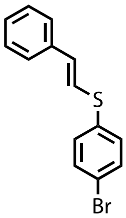

δH (400 MHz, CDCl3)

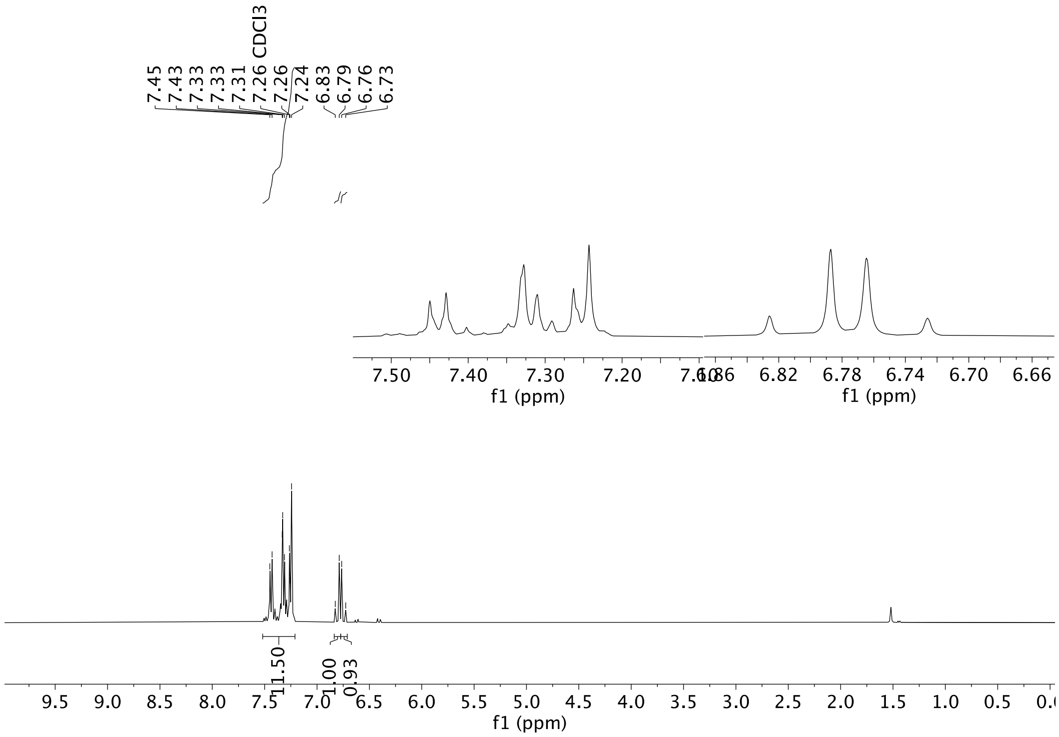

δC (101 MHz, CDCl3)

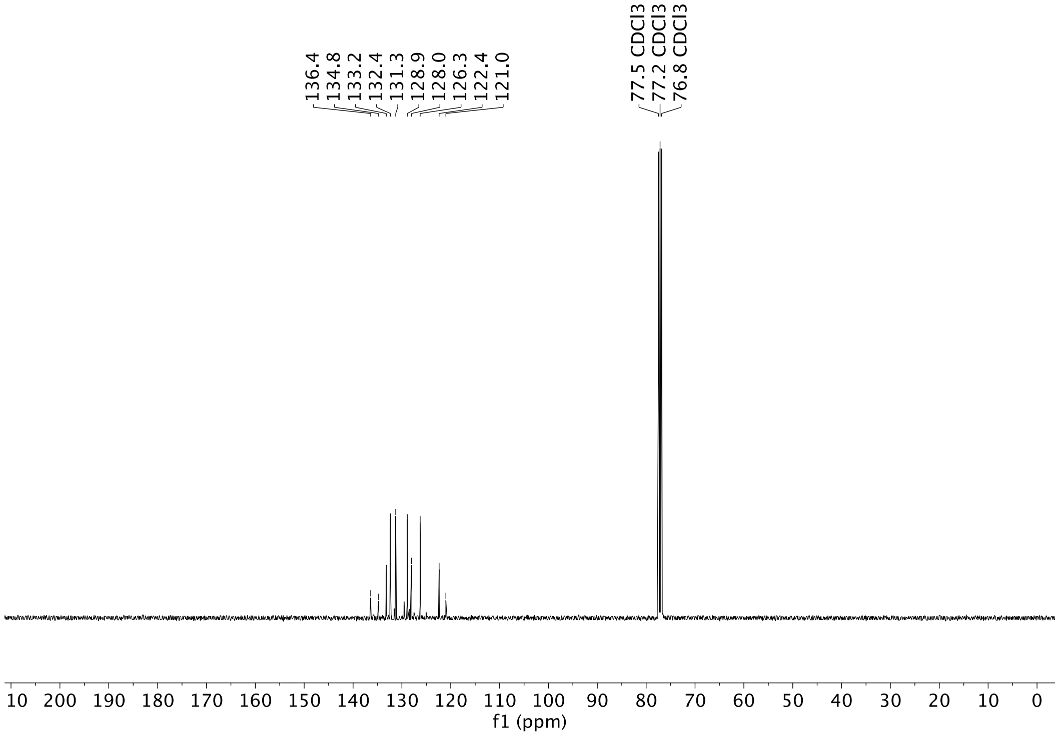

11 References

[1] A. I. Vogel, B. S. Furniss, A. J. Hannaford, V. Rogers, P. W. G. Smith, A. R. Tatchell, *Vogel's Textbook of Practical Organic Chemistry*, Prentice Hall, Harlow, **1996**.

[2] J.-F. Gestin, F. Guerard, A. Faivre-Chauvet, *WO/2011/095517*, **2011**.

[3] Q. Peng, Y. Yuan, H. Zhang, S. Bo, Y. Li, S. Chen, Z. Yang, X. Zhou, Z. X. Jiang, *Org. Biomol. Chem.* **2017**, *15*, 6441–6446.

[4] S. M. Masoud, M. A. Topchiy, A. S. Peregudov, T. Roisnel, P. H. Dixneuf, C. Bruneau, S. N. Osipov, *J. Fluor. Chem.* **2017**, *200*, 66–76.

[5] A. Whyte, M. E. Olson, M. Lautens, *Org. Lett.* **2018**, *20*, 345–348.

[6] R. L. Amey, J. C. Martin, *J. Org. Chem.* **1979**, *44*, 1779–1784.

[7] T. M. Milzarek, N. P. Ramirez, X. Y. Liu, J. Waser, *Chem. Commun.* **2023**, *59*, 12637–12640.

[8] S. Tsuzuki, S. Sakamoto, K. Maruoka, *Chem. Lett.* **2020**, *49*, 633–638.

[9] A. J. Blake, A. Novak, M. Davies, R. I. Robinson, S. Woodward, *Synth. Commun.* **2009**, *39*, 1065–1075.

[10] K. Bhaskar Pal, E. M. Di Tommaso, A. K. Inge, B. Olofsson, *Angew. Chem. Int. Ed.* **2023**, *62*, e202301368.

[11] R. A. Moss, B. Wilk, K. Krogh-Jespersen, J. T. Blair, J. D. Westbrook, *J. Am. Chem. Soc.* **1989**, *111*, 250–258.

[12] E. Dubost, V. Babin, F. Benoist, A. Hébert, P. Barbey, C. Chollet, J. P. Bouillon, A. Manrique, G. Pieters, F. Fabis, T. Cailly, *Org. Lett.* **2018**, *20*, 6302–6305.

[13] F. Péron, C. Fossey, J. Sopkova-Deoliveirasantos, T. Cailly, F. Fabis, *Chem. Eur. J.* **2014**, *20*, 7507–7513.

[14] N. Radhoff, A. Studer, *Angew. Chem. Int. Ed.* **2021**, *60*, 3561–3565.

[15] C. Morrill, J. E. Gillespie, R. J. Phipps, *Angew. Chem. Int. Ed.* **2022**, *61*, e202204025.

[16] A. Boelke, L. D. Caspers, B. J. Nachtsheim, *Org. Lett.* **2017**, *19*, 5344–5347.

[17] L. Castoldi, E. M. Di Tommaso, M. Reitti, B. Gräfen, B. Olofsson, *Angew. Chem. Int. Ed.* **2020**, *59*, 15512–15516.

[18] J. P. Brand, J. Waser, *Angew. Chem. Int. Ed.* **2010**, *49*, 7304–7307.

[19] G. Pisella, A. Gagnebin, J. Waser, *Org. Lett.* **2020**, *22*, 3884–3889.

[20] E. Stridfeldt, A. Seemann, M. J. Bouma, C. Dey, A. Ertan, B. Olofsson, *Chem. Eur. J.* **2016**, *22*, 16066–16070.

[21] M. J. Bouma, B. Olofsson, *Chem. A Eur. J.* **2012**, *18*, 14242–14245.

[22] L. Castoldi, E. M. Di Tommaso, M. Reitti, B. Gräfen, B. Olofsson, *Angew. Chem. Int. Ed.* **2020**, *59*, 15512–15516.

[23] G. M. Sheldrick, *Acta Crystallogr. Sect. A Found. Crystallogr.* **2008**, *64*, 112–122.

[24] A. L. Spek, *J. Appl. Crystallogr.* **2003**, *36*, 7–13.
